# Bayesian Inference of Spatial Organizations of Chromosomes

**DOI:** 10.1371/journal.pcbi.1002893

**Published:** 2013-01-31

**Authors:** Ming Hu, Ke Deng, Zhaohui Qin, Jesse Dixon, Siddarth Selvaraj, Jennifer Fang, Bing Ren, Jun S. Liu

**Affiliations:** 1 Department of Statistics, Harvard University, Cambridge, Massachusetts, United States of America; 2 Department of Biostatistics and Bioinformatics, Rollins School of Public Health, Emory University, Atlanta, Georgia, United States of America; 3 Ludwig Institute for Cancer Research, La Jolla, California, United States of America; 4 Medical Scientist Training Program, University of California, San Diego, La Jolla, California, United States of America; 5 Biomedical Sciences Graduate Program, University of California, San Diego, La Jolla, California, United States of America; 6 Bioinformatics and Systems Biology Graduate Program, University of California, San Diego, La Jolla, California, United States of America; 7 University of California, San Diego School of Medicine, Department of Cellular and Molecular Medicine, Institute of Genomic Medicine, UCSD Moores Cancer Center, La Jolla, California, United States of America; Weizmann Institute of Science, Israel

## Abstract

Knowledge of spatial chromosomal organizations is critical for the study of transcriptional regulation and other nuclear processes in the cell. Recently, chromosome conformation capture (3C) based technologies, such as Hi-C and TCC, have been developed to provide a genome-wide, three-dimensional (3D) view of chromatin organization. Appropriate methods for analyzing these data and fully characterizing the 3D chromosomal structure and its structural variations are still under development. Here we describe a novel Bayesian probabilistic approach, denoted as “Bayesian 3D constructor for Hi-C data” (BACH), to infer the consensus 3D chromosomal structure. In addition, we describe a variant algorithm BACH-MIX to study the structural variations of chromatin in a cell population. Applying BACH and BACH-MIX to a high resolution Hi-C dataset generated from mouse embryonic stem cells, we found that most local genomic regions exhibit homogeneous 3D chromosomal structures. We further constructed a model for the spatial arrangement of chromatin, which reveals structural properties associated with euchromatic and heterochromatic regions in the genome. We observed strong associations between structural properties and several genomic and epigenetic features of the chromosome. Using BACH-MIX, we further found that the structural variations of chromatin are correlated with these genomic and epigenetic features. Our results demonstrate that BACH and BACH-MIX have the potential to provide new insights into the chromosomal architecture of mammalian cells.

## Author Summary

Understanding how chromosomes fold provides insights into the complex relationship among chromatin structure, gene activity and the functional state of the cell. Recently, chromosome conformation capture based technologies, such as Hi-C and TCC, have been developed to provide a genome-wide, high resolution and three-dimensional (3D) view of chromatin organization. However, statistical methods for analyzing these data are still under development. Here we propose two Bayesian methods, BACH to infer the consensus 3D chromosomal structure and BACH-MIX to reveal structural variations of chromatin in a cell population. Applying BACH and BACH-MIX to a high resolution Hi-C dataset, we found that most local genomic regions exhibit homogeneous 3D chromosomal structures. Furthermore, spatial properties of 3D chromosomal structures and structural variations of chromatin are associated with several genomic and epigenetic features. Noticeably, gene rich, accessible and early replicated genomic regions tend to be more elongated and exhibit higher structural variations than gene poor, inaccessible and late replicated genomic regions.

## Introduction

The spatial organization of a genome plays an important role in gene regulation, DNA replication, epigenetic modification and maintenance of genome stability [1]–[5]. Understanding three-dimensional (3D) chromosomal structures and chromatin interactions is therefore essential for decoding and interpreting functions of the genome. Traditionally, the 3D organization of chromosomes has been studied by microscopic and cytogenic methods such as florescent in situ hybridization (FISH). Several FISH studies have shown that the global 3D chromosomal structures are highly dynamic [6]–[8]. However, due to the limitation of low throughput, low resolution FISH data, the 3D chromosomal structures at the fine scale are not fully understood. In particular, whether chromatin exhibits a consensus local 3D chromosomal structure is still under debate. More recently, higher throughput, higher resolution approaches based on chromosome conformation capture (3C) such as Hi-C [9] and TCC [10] allow genome-wide mapping of chromatin interactions. The chromatin interactions captured by Hi-C and TCC experiments, which are represented by the contact matrix in the original Hi-C study [9], provide an unprecedented opportunity for inferring 3D chromosomal structures at the fine resolution scale.

Much progress has been made in recent years to reconstruct 3D chromosomal structures from the Hi-C data by translating the observed chromatin contact frequency between two genomic loci to the population average spatial distance between them. Bau and colleagues [11] translated the read counts in the contact matrix to spatial constraints of 3D chromosomal structures and used the software Integrated Modeling Platform (IMP) [12] to solve a constrained optimization problem. Duan et al. [13] devised a set of constraints for all loci of the genome, and solved a similar constrained optimization problem using an open-source software IPOPT [14]. Similar optimization-based approaches have also been used in studies of the fission yeast genome [15]. Kalhor et al. [10] proposed another optimization-based approach which correlates contact frequencies with the presence or absence of chromatin contacts instead of average spatial distances. More recently, Rousseau et al. [16] developed a probabilistic model linking Hi-C data to spatial distances and designed a Markov-chain Monte Carlo-based method named MCMC5C. Different from the optimization-based approaches, MCMC5C models the uncertainties of spatial distances between two loci by assuming that the number of reads spanning those two loci follows a Gaussian distribution.

However, all the existing methods have several limitations. First, as pointed out by Yaffe and Tanay [17], the raw data obtained from Hi-C experiments exhibit multiple layers of systematic biases, such as restriction enzyme cutting frequencies, GC content and sequence uniqueness. None of the existing methods take these systematic biases into consideration. Second, optimization-based methods are prone to be trapped in local modes due to the ultra-high dimensionality and the prohibitively large search space. Third, MCMC5C suffers from the difficulty in estimating the Gaussian variance of each read count since the single Hi-C contact matrix does not provide enough information for variance estimation. Furthermore, except for MCMC5C, none of these existing methods comes with a stand-alone software [16].

More importantly, all of the existing methods focus on reconstructing consensus 3D chromosomal structures, but pay little attention to evaluating magnitudes of structural variations of chromatin at different resolution scales. To quantify structural variations of chromatin, the optimization-based methods usually require a large number of parallel runs, which is computationally intensive and not directly interpretable. Similarly, the Gaussian model in MCMC5C is derived from a consensus 3D chromosomal structure, which cannot be used to measure structural variations of chromatin either.

Since chromatin interactions captured by Hi-C experiments come from a cell population instead of a single cell, it is challenging to study structural variations of chromatin from the Hi-C data. When the cell population consists of multiple sub-populations, of which each corresponds to a distinct 3D chromosomal structure, the Hi-C data can only be interpreted as a measurement of the population average effect. The Hi-C data of mammalian genomes is further complicated by the fact that the pair of homologous chromosomes cannot be distinguished from each other without genotype information. Without fully characterizing structural variations of chromatin in a cell population, the consensus 3D chromosomal structure inferred from the Hi-C data is not directly interpretable or even misleading.

Although the global 3D chromosomal structure is indeed quite dynamic in a cell population, the local 3D chromosomal structure could be homogeneous. A recent study [18] on a high resolution Hi-C dataset has discovered that mammalian genomes are composed of thousands of mega-base-sized, evolutionarily conservative topological domains, which appear to serve as units of genomic organization and perhaps function. These findings motivate the hypothesis that each topological domain may share a consensus 3D chromosomal structure in order to keep its conservative functional forms. For local genomic regions where this hypothesis holds true, the mixture of cell populations and the ambiguity of homologous chromosomes will no longer be major barriers for 3D modeling based on Hi-C data.

In this work, we test the hypothesis of consensus 3D structure at the topological domain scale via rigorous statistical analysis of Hi-C data. To achieve this goal, we propose two integrated probabilistic approaches called BACH (which is the short name for “**Ba**yesian 3D **C**onstructor for **H**i-C data”) and BACH-MIX. It should be noted that our approach is closely related to inferential structure determination (ISD) [19], a Bayesian approach developed to study macromolecular structure. In the BACH algorithm, we assume that the local genomic region (i.e., a topological domain) of interest exhibits a consensus 3D chromosomal structure in a cell population, and employ efficient Markov chain Monte Carlo (MCMC) computational tools to infer the underlying consensus 3D chromosomal structure. In the BACH-MIX algorithm, we assume that the genomic region of interest consists of multiple distinct 3D chromosomal structures, and explicitly model structural variations of chromatin using a mixture component model. By comparing the goodness of fit of BACH and BACH-MIX for the same genomic region via statistical model selection principles, we provide a quantitative approach to evaluate structural variations of chromatin for any given local genomic region.

Applying BACH and BACH-MIX to a high resolution Hi-C dataset, we found that BACH, instead of BACH-MIX, is preferred in about half of the topological domains. Of the topological domains in which BACH-MIX fits the data better, most contain one dominant sub-population, whose 3D chromosomal structure can be reconstructed by the BACH algorithm. These results suggest that most topological domains exhibit homogeneous 3D chromosomal structures in a cell population. We also found that geometrical properties of these topological domains, particularly the shape and the structural variations, are associated with several genomic and epigenetic features. Furthermore, we found significantly lower structural variations at domain center regions than at domain boundary regions.

## Results

### The BACH algorithm

The BACH algorithm takes the chromosomal contact matrix generated by Hi-C or TCC experiments and local genomic features [17], [20] (restriction enzyme cutting frequencies, GC content and sequence uniqueness) as input, and produces, via MCMC computation, the posterior distribution of 3D chromosomal structures (Methods). In the BACH algorithm, we assume that there exists a consensus 3D chromosomal structure in a cell population (this assumption will be relaxed later in the BACH-MIX algorithm). Furthermore, we assume that the number of sequencing reads spanning two genomic loci follows a Poisson distribution, where the Poisson rate is negatively associated with the corresponding spatial distance between them and is also affected by a few other factors. BACH can be used to reconstruct consensus 3D chromosomal structures from the Hi-C contact matrix, and infer the uncertainties of the spatial distance between any two genomic loci from the corresponding posterior distribution. Simulation studies have shown that the BACH algorithm works well under the posited model (Text S1).

Compared to other published methods, BACH has the following advantages: (1) It explicitly models and corrects known systematic biases associated with Hi-C data, such as restriction enzyme cutting frequencies, GC content and sequence uniqueness [17], [20]; (2) It utilizes a Poisson model that better fits the count data generated from Hi-C experiments than the Gaussian model used in MCMC5C, and performs more robustly when applied to several experimental datasets (see the following RESULTS section for validation); (3) It employs advanced MCMC techniques, such as Sequential Monte Carlo and Hybrid Monte Carlo (see Text S1 for details), that significantly improve the efficiency in exploring the vast space of possible models [21].

### The BACH-MIX algorithm

In the BACH algorithm, we assume that chromosomal regions of interest exhibit a consensus 3D chromosomal structure in a cell population. However, this assumption may not be true, because chromosomal regions may exist in multiple inter-convertible configurations. To test the consensus 3D chromosomal structure assumption and study structural variations of chromatin in a cell population, we propose a variant algorithm called BACH-MIX (Methods). In BACH-MIX, we assume that the genomic region of interest is composed of two adjacent sub-regions, each with a rigid consensus 3D structure, but the spatial arrangement of the two sub-structures can vary in a cell population. BACH-MIX models the uncertainty of the spatial arrangement between the two sub-structures by a mixture component model, where each component corresponds to one specific spatial arrangement. The weight of each component represents the proportion of that component in a cell population. Clearly, BACH is a special case of BACH-MIX, in which the number of the mixture component is one. We use the statistical model selection criterion, the Akaike information criterion (AIC) [22], to determine whether BACH or BACH-MIX fit the data better, so as to infer whether the structure is homogeneous (having a consensus) or variable.

BACH-MIX contains two types of parameters: the parameters to determine the local consensus 3D chromosomal structures of the two adjacent sub-regions, and the parameters to determine the spatial arrangement of the two adjacent sub-regions. In practice, the local 3D chromosomal structures of the two adjacent sub-regions can be estimated by applying BACH twice separately, each to the contact map of one sub-region. The main computation in BACH-MIX is to estimate the parameters corresponding to each spatial arrangement of the two adjacent sub-structures.

A spatial arrangement of the two adjacent sub-structures can be represented by a rotation matrix with three Euler angles [23]. We also take into account mirror symmetry structures that cannot be explained by rotations. To simplify the computation, we discretize the range of each Euler angle into four bins of equal sizes, and approximate the collection of distinct 3D chromosomal structures in a cell population by 104 spatial arrangements of two adjacent sub-regions (Text S1). The BACH-MIX algorithm takes 3D chromosomal structures BACH predicted for two adjacent sub-regions and the corresponding local genomic features [17] (restriction enzyme cutting frequencies, GC content and sequence uniqueness) as input, and produces the posterior distribution of the spatial arrangement of the two sub-regions, quantified by the proportion of each of the 104 orientations between the two. Simulation studies have shown that the BACH-MIX algorithm works well under the posited model (Text S1).

In practice, a majority of the 104 spatial arrangements of the two adjacent sub-regions are insignificant in terms of having very low proportions. To overcome over-fitting, we adopt a two-step procedure to achieve sparsity: first, we apply the full BACH-MIX model with 104 spatial arrangements to estimate the proportion for each of them; second, we remove insignificant spatial arrangements whose proportion is less than 1%, and re-estimate the proportion for the significant spatial arrangements.

### Most topological domains exhibit homogeneous 3D chromosomal structures

We applied BACH and BACH-MIX to a dataset recently generated in our lab [18] from a mouse embryonic stem cell (mESC) line. The dataset includes 476 million reads obtained from two biological replicates processed with the use of the restriction enzyme HindIII (referred to as the HindIII sample); and 237 million reads in another biological replicate processed with the use of the restriction enzyme NcoI (referred to as the NcoI sample). To the best of our knowledge, this dataset provides the highest sequencing depth of a mammalian genome to date. Previous analysis of this dataset showed that the mouse genome is composed of 2,200 topological domains characterized by high frequencies of intra-domain interactions but infrequent inter-domain interactions [18].

We conducted a genome-wide analysis by applying BACH and BACH-MIX to this high-resolution mESC Hi-C dataset. Both BACH and BACH-MIX were applied to the 40 KB resolution Hi-C contact matrices. In the preprocessing procedure, we filtered out 300 topological domains whose length is less than 400 KB or do not contain known mouse gene (13.64% out of total 2,200 domains). We also filtered out a subset of 40 KB genomic loci within each topological domain according to restriction enzyme cutting frequencies (number of fragment end < = 5), GC content (< = 0.3) and sequence uniqueness (mappability score < = 0.8) (Figure S1), and created the 40 KB resolution Hi-C contact matrix for each topological domain. We then applied BACH to each of the remaining 1,900 topological domains to infer its 3D chromosomal structure.

To validate the spatial distances inferred by the BACH algorithm, we compared the spatial distances BACH predicted (referred to as the BACH distances) to the spatial distances measured by FISH [24] (referred to as the FISH distances). In the HindIII sample, the Pearson correlation coefficient between the BACH distances and the FISH distances is 0.88 (95% credible interval is [0.83, 0.92]). In the NcoI sample, the Pearson correlation coefficient between the BACH distances and the FISH distances is 0.83 (95% credible interval is [0.67, 0.93]). These results suggest that the spatial distances BACH predicted are consistent with the spatial distances measured by FISH (Text S1 and Figure S2). As a comparison, we applied MCMC5C and obtained the corresponding predictions of spatial distances (referred to as the MCMC5C distances). The Pearson correlation coefficients between the MCMC5C distances and the FISH distances are 0.79 and 0.11 in the HindIII sample and the NcoI sample, respectively, which are much worse than those of the BACH's results (z-test p-values <2.4e-5). In addition, we applied a modified BACH algorithm without bias correction and found it still achieved higher correlation with the FISH distances than MCMC5C (Text S1).

In the previous analysis, we obtained the 3D chromosomal structure predicted by BACH for each topological domain. Next, we divided each topological domain into two sub-regions of equal sizes, and applied BACH-MIX to infer the spatial arrangement of the two sub-regions. We evaluated the goodness of fit of the BACH model and the BACH-MIX model for each of these 1,900 topological domains in terms of AIC, which penalizes the log-likelihood of a model with the number of parameters in the model. A smaller AIC indicates a better model fitting. In the HindIII sample, BACH achieved smaller AIC than BACH-MIX in 875 out of 1,900 (46.05%) topological domains. For the rest 1,025 topological domains where BACH-MIX fits the data better than BACH, 487 topological domains have one dominant spatial arrangement of the two sub-regions with proportion greater than 80%. In 482 out of these 487 topological domains, the dominant 3D chromosomal structure can be captured by BACH. Therefore, BACH can reconstruct the consensus structure or the dominant structure in 1,357 topological domains (71.42% of 1,900 topological domains). We obtained consistent results in the NcoI sample. In the NcoI sample, BACH achieved smaller AIC than BACH-MIX in 1,156 out of 1,900 (60.84%) topological domains. For the rest 744 topological domains where BACH-MIX fits the data better than BACH, 394 topological domains have one dominant spatial arrangement of the two sub-regions with proportion greater than 80%. In 393 out of these 394 topological domains, the dominant 3D chromosomal structure can be captured by BACH. Therefore, BACH can reconstruct the consensus structure or the dominant structure in 1,549 topological domains (81.53% of 1,900 topological domains).

### Structural properties of topological domains correlate with genomic and epigenetic features

In the following analysis, we focus on 1,199 (the overlap between 1,357 topological domains in the HindIII sample and 1,549 topological domains in the NcoI sample, 63.11% out of 1,900) topological domains in which BACH can reconstruct the consensus 3D chromosomal structure or the 3D chromosomal structure of the dominant sub-population in both HindIII sample and NcoI sample. To summarize the structural properties of topological domains, we approximated each 3D chromosomal structure BACH predicted (40 KB resolution) by a cylinder, and computed the ratio between its height and diameter, abbreviated as *HD ratio* (Methods). Domains with higher HD ratios are more elongated. HD ratios of the structures inferred from the HindIII sample and the NcoI sample are highly reproducible (Pearson correlation coefficients = 0.76, p-value<2.2e-16).

**Figure 1:**
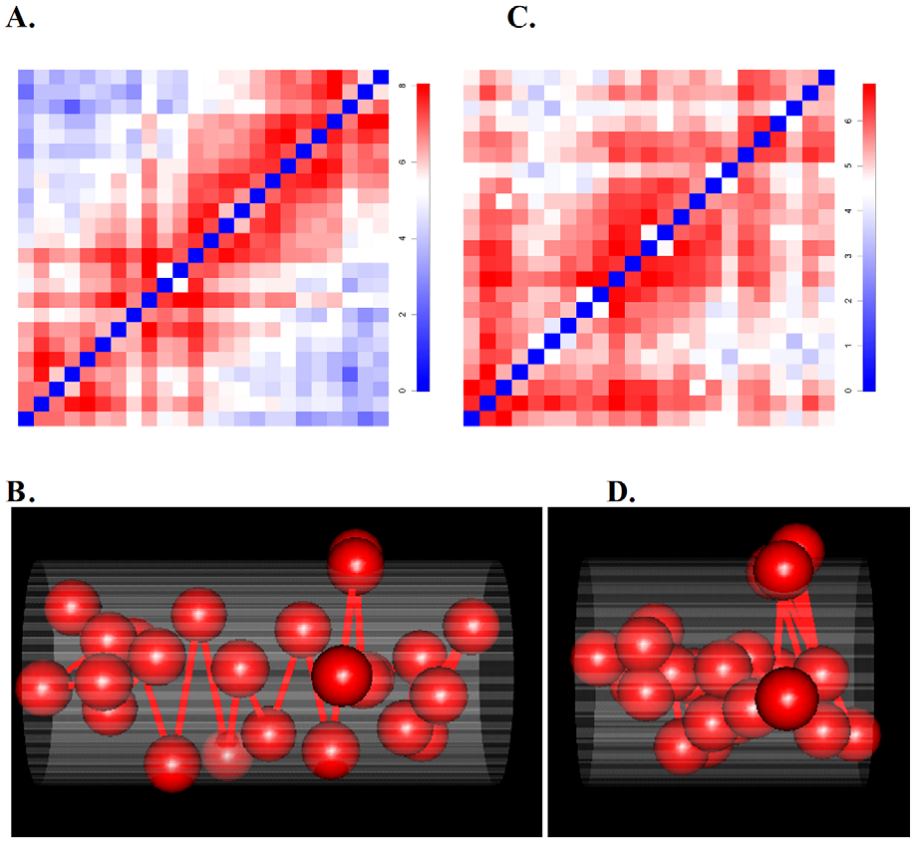
Two illustrative examples of 3D models for two topological domains using BACH. Two illustrative examples in the HindIII sample: one for a more elongated 1 MB domain (chromosome 18, 33,960,000∼34,960,000) belonging to compartment A, the other for a less elongated 1 MB domain (chromosome 7, 62,040,000∼63,040,000) belonging to compartment B. In Figure 1B and Figure 1D, each sphere represents a 40 KB genomic region. All spheres are of equal size. In Figure 1B and Figure 1D, the x axis is the direction of the first principle component. The diameters of two fitted cylinders (grey) are set to be one. The height of the fitted cylinder in Figure 1B is 1.89 times larger than that in Figure 1D. The rank in descending order among the selected 1,199 domains was used to measure the relative magnitudes of genomic and epigenetic features (Table S2). The more elongated 1 MB domain has a high gene density, high gene expression, high H3K36me3, high H3K4me3, high RNA polymerase II, high chromatin accessibility, early DNA replication time, low H3K9me3, low H4K20me3 and low genome-nuclear lamina interaction. The 3D chromosomal structure BACH predicted for this domain (Figure 1B) has a high HD ratio (HD ratio = 2.16, rank = 146). The less elongated 1 MB domain has a low gene density, low gene expression, low H3K36me3, low H3K4me3, low RNA polymerase II, low chromatin accessibility, late DNA replication time, high H3K9me3, high H4K20me3 and high genome-nuclear lamina interaction. The 3D chromosomal structure BACH predicted for this domain (Figure 1D) has a low HD ratio (HD ratio = 1.14, rank = 842). The more elongated 1 MB domain has median H3K27me3 signal, while the less elongated 1 MB domain has low H3K27me3 signal. These results can be partially explained by the weak correlation between the HD ratio and H3K27me3 (Table S1, Pearson correlation coefficients = 0.14, p-value = 4.9e-7). They are also consistent with the results in the human Hi-C study demonstrating weak enrichment of H3K27me3 in compartment A [9]. (**A**) 40 KB resolution Hi-C contact matrix of a more elongated domain belonging to compartment A. The color scheme is proportional to Log2 read counts. (**B**) The 3D chromosomal structure BACH predicted for the domain described in Figure 1A. HD ratio = 2.16. (**C**) 40 KB resolution Hi-C contact matrix of a less elongated domain belonging to compartment B. The color scheme is proportional to Log2 read counts. (**D**) The 3D chromosomal structure BACH predicted for the domain described in Figure 1C. HD ratio = 1.14.

To evaluate the relationship between structural properties of chromatin (measured by HD ratio) and its functional forms at the topological domain scale, we collected genomic and epigenetic features for each topological domain, including gene density (UCSC reference genome mm9), gene expression [25], five histone modification marks (H3K36me3 [26], H3K27me3 [27], H3K4me3 [25], H3K9me3 [28] and H4K20me3 [27]), RNA polymerase II [25], chromatin accessibility [29], genome-nuclear lamina interaction [30] and DNA replication time [31]. By computing the correlation between the HD ratio and each of the genomic and epigenetic features, we found that the HD ratio is highly significantly and positively correlated with gene density, gene expression, transcription elongation histone modification mark H3K36me3, repressive histone modification mark H3K27me3, promoter mark H3K4me3, RNA polymerase II, accessible chromatin and early replicated genomic regions, and negatively associated with heterochromatin marks H3K9me3, H4K20me3 and lamina associated domains (Table S1). These correlations are similarly computed based on either the HindIII sample or the NcoI sample. Two illustrative examples are shown in Figure 1 and Table S2. Consistent with other existing biological evidences, these results demonstrate that the gene rich, actively transcribed, accessible, and early replicated chromatin tends to be more elongated than the gene poor, lowly transcribed, inaccessible and late replicated chromatin, which is consistent with previous FISH experiments [32].

The original Hi-C study [9] has shown that chromatin interactions closely correlate with the genomic and epigenetic features. By applying the principle component analysis (PCA) method to the Hi-C data, Lieberman-Aiden et al. [9] demonstrated that two compartments (compartment A and compartment B) in the mammalian genome can be obtained, where compartment A is strongly associated with open, accessible, and actively transcribed chromatin [33]. Following their strategy, we also applied the PCA method to the mESC Hi-C dataset [18] and obtained the compartment label (A or B) for each topological domain. The compartments A and B represent a high level interpretation of the Hi-C data, but do not inform us on the details of chromatin folding. Recently, we and others showed that compartments A and B could be further partitioned into topological domains, which are mega-base-sized, self-interacting genomic regions [18], [34]. Using BACH, we generated 3D models of topological domains, and found that topological domains in compartment A are significantly more elongated than those in compartment B. In the HindIII sample, mean HD ratios for domains in compartment A and compartment B are 1.81 and 1.34, respectively (two sample t-test p-value<2.2e-16). Similarly, in the NcoI sample, mean HD ratios for domains in compartment A and compartment B are 1.76 and 1.26, respectively (p-value<2.2e-16). Two illustrative examples are shown in Figure 1 and Table S2. These results suggest that the HD ratio obtained in the BACH algorithm provides an intuitive visual interpretation of the Hi-C data.

### Structural variations of topological domains correlate with genomic and epigenetic features

We further study the structural variations of chromatin in a cell population. We first selected 562 topological domains with size larger than 1 MB, and applied BACH and BACH-MIX to the 1 MB region around the center of each selected domain center region. Additionally, we used 985 domain boundaries with size shorter than 40 KB as the control group, and applied BACH and BACH-MIX to the 1 MB region around each selected domain boundary region. We divided each 1 MB genomic region (domain center/boundary region) into two 500 KB adjacent sub-regions, predicted the 3D structure of each sub-region by BACH, and then inferred the spatial arrangements of the two sub-structures. Both BACH and BACH-MIX were applied to the 40 KB resolution Hi-C contact matrices.

Among all the possible spatial arrangements of two adjacent genomic regions, we defined the effective structures as those with their posterior mean proportions greater than 5%, and report the number of effective structures at each locus. A locus with a smaller number of effective structures exhibits lower structural variations than a locus with a larger number of effective structures. In the HindIII sample, the average number of effective structures is 2.20 for the domain center regions, and 2.82 for the domain boundary regions (Figure S3A, two sample t-test p-value<2.2e-16). Similarly, in the NcoI sample, the average number of effective structures is 2.07 for the domain center regions, and 2.54 for the domain boundary regions (Figure S3B, two sample t-test p-value = 5.2e-13). We changed the threshold for the effective structure to 10% and 1%, and observed consistent results (Figure S3 and Table S3). These results suggest that domain center regions exhibit lower structural variations than domain boundary regions.

**Figure 2:**
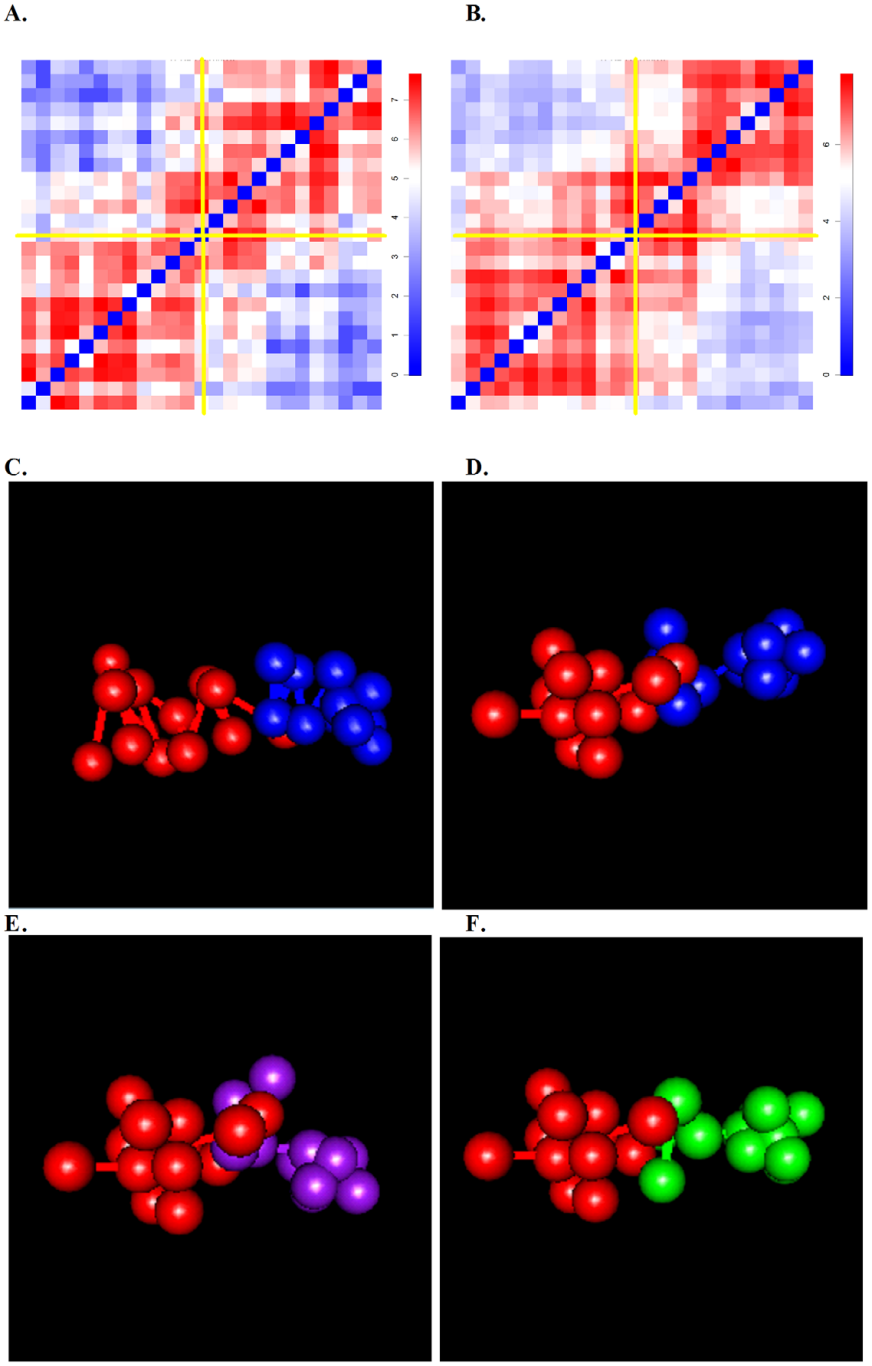
Domain center regions exhibit lower structural variations than domain boundary regions. Two illustrative examples in the HindIII sample: one for the domain center region (Chromosome 2, 117,580,000∼118,580,000) with low structural variations, and the other for the domain boundary region (Chromosome 1, 135,540,000∼136,540,000) with high structural variations. In Figure 2C∼Figure 2F, each sphere represents a 40 KB genomic region. All spheres are of equal size. (**A**) 40 KB resolution Hi-C contact map of a 1 MB domain center region in the HindIII sample. The color scheme is proportional to Log2 read counts. Two yellow lines divide the Hi-C contact map of a 1 MB region into two 500 KB adjacent sub-regions. (**B**) 40 KB resolution Hi-C contact map of a 1 MB domain boundary region in the HindIII sample. The color scheme is proportional to Log2 read counts. Two yellow lines divide the Hi-C contact map of a 1 MB region into two 500 KB adjacent sub-regions. (**C**) The effective structure BACH-MIX predicted (proportion = 0.99) for the domain center region. Red spheres and lines represent the bottom left region in Figure 2A, blue spheres and lines represent the top right region in Figure 2A. (**D**) The first effective structure BACH-MIX predicted (proportion = 0.77) for the domain boundary region. Red spheres and lines represent the bottom left region in Figure 2B, blue spheres and lines represent the top right region in Figure 2B. (**E**) The second effective structure BACH-MIX predicted (proportion = 0.14) for the domain boundary region. Red spheres and lines represent the bottom left region in Figure 2B, purple spheres and lines represent the top right region in Figure 2B. (**F**) The third effective structure BACH-MIX predicted (proportion = 0.08) for the domain boundary region. Red spheres and lines represent the bottom left region in Figure 2B, green spheres and lines represent the top right region in Figure 2B.

Figure 2 shows two illustrative examples in the HindIII sample, one for the domain center region (Chromosome 2, 117,580,000∼118,580,000, Figure 2A), and one for the domain boundary region (Chromosome 1, 135,540,000∼136,540,000, Figure 2B). Under threshold 5%, BACH-MIX identified one effective structure for the domain center region with proportion 99% (Figure 2C), and three effective structures for the domain boundary region, with proportions 77% (Figure 2D), 14% (Figure 2E) and 8% (Figure 2F), respectively.

Next, we evaluated the relationship between structural variations of topological domains and its functional forms. We divided the 562 selected domain center regions into two groups, regions with high structural variations (i.e., containing multiple effective structures, threshold = 5%) and regions with low structural variations (i.e., containing one effective structure, threshold = 5%), and compared the genomic and epigenetic features between these two groups (Table S4). We observed significant enrichment of gene density, transcription elongation histone modification mark H3K36me3, repressive histone modification mark H3K27me3, promoter mark H3K4me3, RNA polymerase II, accessible chromatin and early replicated genomic regions in regions with high structural variations, and significant enrichment of heterochromatin marks H3K9me3, H4K20me3 and genome-nuclear lamina interaction in regions with low structural variations. Noticeably, we did not observe statistically significant association between gene expression levels and structural variations. These results suggest that gene rich, accessible and early replicated chromatins are more likely to exhibit multiple structural configurations than gene poor, inaccessible and late replicated chromatins.

### A two-step procedure to quantify the structure variations of the whole chromosome

Although it is widely accepted that the chromatin structure is highly dynamic, it is unclear whether the cell population contains one dominant chromosomal structure, or multiple distinct chromosomal structures with comparable mixture proportions. To quantify structural variations of the whole chromosome in the cell population, we designed the following two-step procedure. In the first step, we applied BACH to the whole chromosome scale Hi-C contact matrix and obtained a predicted 3D chromosomal structure (the mode of the first BACH posterior distribution, referred to as 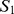). Then, we computed the expected Hi-C contact matrix based on this predicted structure 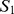. In the second step, we defined the residual matrix as the difference between the original Hi-C contact matrix and half of the expected Hi-C contact matrix, and applied BACH again to the residual matrix to obtain another predicted 3D chromosomal structure (the mode of the second BACH posterior distribution, referred to as 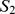). In order to avoid the possibility of algorithmic artifacts, we ran 100 parallel chains for our two-step procedure using a large variety of initial structures and chose the structures with the highest posterior probabilities.

If there exists a dominant chromosomal structure (referred to as 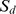) in the cell population, we will expect that 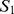 and 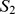 are close to each other, since 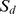 is still the dominant chromosomal structure in the residual matrix. On the other hand, if there is no such dominant chromosomal structure in the cell population, we will expect that 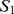 and 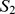 are quite different from each other since the original contact matrix and the residual matrix should have little in common. In practice, the similarity between 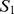 and 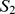 can be measured by the normalized root mean square deviations, i.e., RMSD(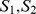) (Methods). Simulation results (Text S1, Figure S4 and Table S5) confirmed that both 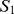 and 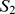 are close to 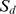 (which also means that RMSD(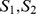) is small) if 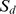 is indeed the dominant chromosomal structure.

In practice, however, we need a reference probability distribution in order to claim that the observed RMSD(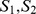) is small enough. Previous studies [35], [36] have shown that the random walk backbone model can be used to approximate the chromatin 3D structure. In this work, we use the empirical distribution of the RMSD between two 3D structures independently generated from the random walk scheme as the reference distribution to judge whether an observed RMSD(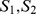) is small enough (Text S1). If the observed RMSD(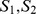) falls within the lower 5% of the reference distribution, we claim that 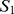 and 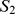 are close enough to each other.

### Long chromosomes may exhibit a dominant 3D structure in the cell population

We applied the above two-step procedure to the real Hi-C data to generate 3D chromosomal structure for each mouse chromosome by treating each topological domain as a basic unit. Figure S5 lists the alignment of two 3D chromosomal structures BACH predicted in the two stages, 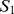 and 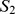, from 20 mouse chromosomes in both HindIII sample and NcoI sample. Tail probabilities of RMSD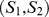 for each chromosome are reported in Table S6. Figure S6 displays the box plots of the twenty RMSD empirical distributions, each corresponding to that between two independently generated random walks of the same length as each mouse chromosome. We found that in long chromosomes (chr 1 to chr 14 and chr X), 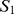 and 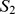 are similar (i.e., RMSD(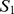,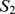) is small, within the tail probability<0.05), suggesting the existence of a dominant 3D chromosomal structure in the cell population. It is worth noting that all these long chromosomes adopt helical structures (Figure S7A), which is unlikely to be coincidental. For short chromosomes, however, RMSD(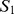,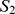) is comparable to that of two independently simulated random walks (tail probability ≥0.05). We conducted similar analysis at different resolution scales by treating two domains or half of a domain as a basic unit, for both the HindIII sample and the NcoI sample. The results were almost identical to the original analysis (Text S1). These results suggest that the whole chromosome scale 3D modeling could be meaningful, especially for long chromosomes (chr 1 to chr 14 and chr X). We did not obtain consistent overall structures in the two-step procedure for short chromosomes. It is likely that such inconsistencies are caused by a lack of “leveraging” information of the Hi-C data when a chromosome is short. By further examining the differences between the two structures obtained by our two-step procedure for these short chromosomes, we observed that the large RMSD is caused by the existence of a few mirror reflections of local structures, implying that, although the local structures can be determined rather well in these chromosomes, there is not enough information to pin down the orientation of these local parts.

To further understand why shorter chromosomes appeared variable in our two-step procedure at the whole chromosome level, we also conducted a local-level structural comparison. In detail, we used a sliding window of ten domains to scan along each chromosome. For each local region of a chromosome covered by the sliding window, we evaluated the structural similarity between 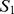 and 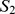 locally (Figure S8), resulting in K - 9 RMSDs for each chromosome, where K is the number of domains of the corresponding chromosome. Now, for all the 20 chromosomes, we found that the local structures in 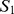 and 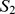 are significantly more similar than those generated from the random walk scheme. More precisely, the distribution of the K - 9 RMSDs for each chromosome is significantly and stochastically smaller than that generated from the random walk scheme (Figure S8), supporting the existence of a dominant structure in the cell population for all chromosomes, at least at a relatively local level (about 10 MB).

A competing method, MCMC5C, has been proposed to generate whole chromosome level 3D models for the human chromosomes [16]. This method, however, does not correct the systematic biases in the Hi-C data. Here we compared whole chromosome level 3D models produced by BACH and MCMC5C for the mouse chromosomes. We used BACH and MCMC5C to generate spatial models of each long chromosome (chr 1 to chr 14 and chr X) by treating each topological domain as a basic unit (Figure S7). The 3D chromosomal structures predicted by BACH from the HindIII sample and NcoI sample are significantly more consistent (measured by RMSD) than those predicted by MCMC5C (paired t-test p-value = 1.4e-7). A modified BACH algorithm without bias correction also outperformed MCMC5C (Text S1). We also conducted the same analysis using a published human Hi-C dataset [9] and found that BACH consistently outperformed MCMC5C (data not shown). The significant improvement of BACH over MCMC5C is likely due to the fact that BACH explicitly integrates the correction of known systematic biases [17], and the Poisson model used in BACH fits the count data of the Hi-C experiment better than the Gaussian model used in MCMC5C. Since other published 3D reconstruction methods do not provide stand-alone software, we were not able to conduct similar comparative studies for them.

### Structural properties of long chromosomes correlate with genomic and epigenetic features

**Figure 3:**
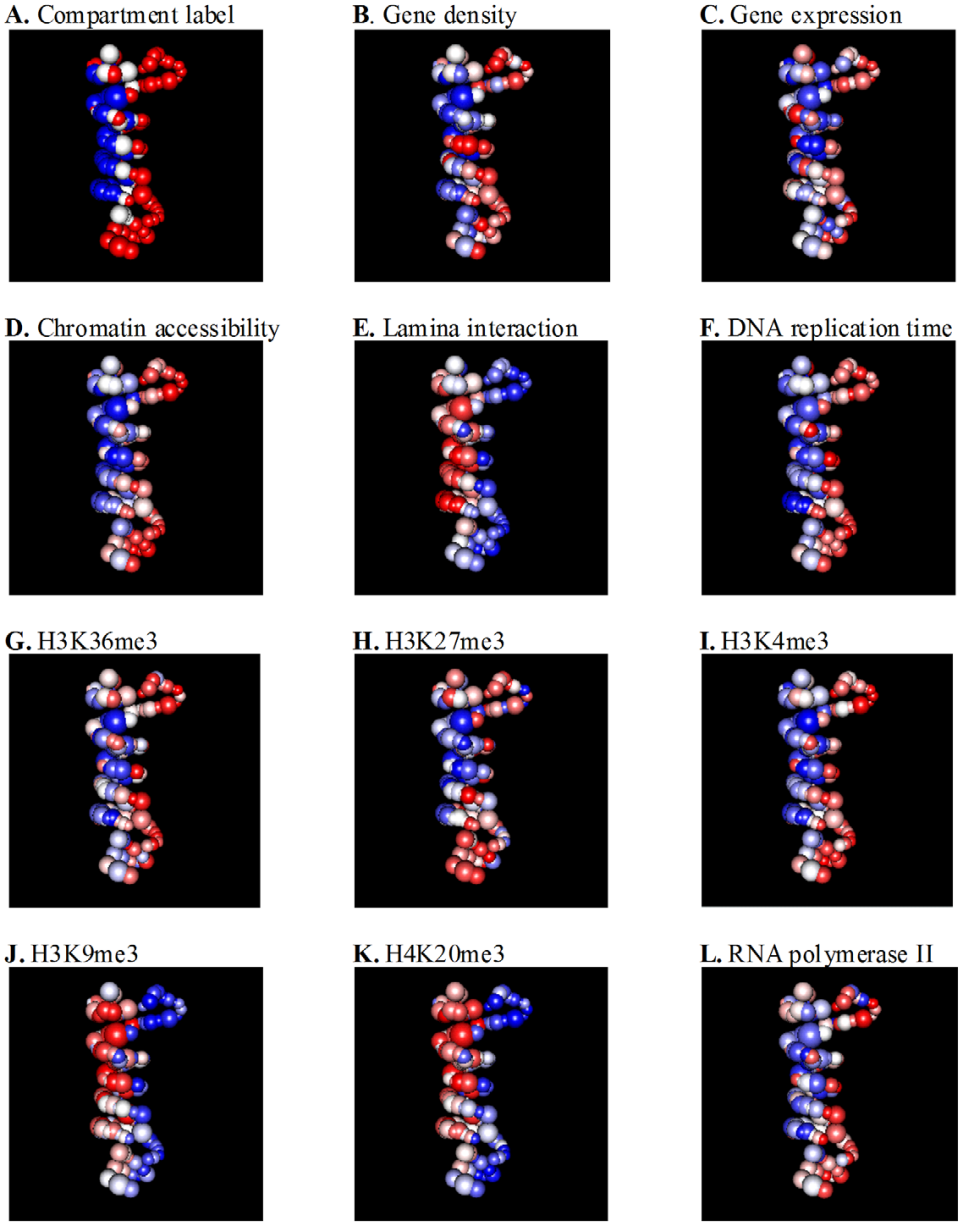
Spatial organization of genomic and epigenetic features. We used the 3D chromosomal structure BACH predicted for chromosome 2 in the HindIII sample as an illustrative example. In Figure 3A∼Figure 3L, each sphere represent a topological domain. The volume of each sphere is proportional to the genomic size of the corresponding topological domain. In Figure 3A, the red, white and blue colors represent topological domains belonging to compartment A, straddle region and compartment B, respectively. Topological domains with the same compartment label tend to locate on the same side of the structure. In Figure 3B∼Figure 3L, the red, white and blue colors represent topological domains with high value of features, median value of features and low value of features, respectively. The color scheme is proportional to the magnitude of the continuous measurement of genetic and epigenetic features. We also report the odds ratio (OR) of the two by two contingency table and the p-value of Fisher's exact test. (**A**) Spatial organization of compartment label. OR = 39.20, p-value = 4.4e-16. (**B**) Spatial organization of gene density. OR = 13.21, p-value = 2.2e-8. (**C**) Spatial organization of gene expression. OR = 4.00, p-value = 0.0012. (**D**) Spatial organization of chromatin accessibility. OR = 26.88, p-value = 5.9e-12. (**E**) Spatial organization of genome-nuclear lamina interaction. OR = 40.00, p-value = 4.9e-13. (**F**) Spatial organization of DNA replication time. OR = 32.00, p-value = 1.1e-10. (**G**) Spatial organization of H3K36me3. OR = 10.91, p-value = 1.0e-7. (**H**) Spatial organization of H3K27me3. OR = 2.17, p-value = 0.0706. (**I**) Spatial organization of H3K4me3. OR = 24.43, p-value = 2.1e-11. (**J**) Spatial organization of H3K9me3. OR = 15.71, p-value = 6.7e-8. (**K**) Spatial organization of H4K20me3. OR = 45.10, p-value = 1.0e-13. (**L**) Spatial organization of RNA polymerase II. OR = 5.47, p-value = 0.0001.

We applied the BACH algorithm to the whole chromosome Hi-C contact matrix, and obtained the predicted 3D chromosomal structures for the 15 long chromosomes (chr 1 to chr 14 and chr X). We first investigated how compartments labeled “A” versus those labeled “B” are distributed spatially in the whole chromosome model. Among all the 1,835 topological domains in chr 1 to chr 14 and chr X, 848 belong to compartment A, 633 belong to compartment B, and the remaining 354 *straddle domains* contain genomic regions from both compartment A and compartment B. For each 3D chromosomal model that BACH predicted, we fitted a plane through the straddle domains using the least square method, and then counted the numbers of topological domains belonging to compartment A and compartment B, respectively, at each side of the fitted plane. The results can be represented by a two-by-two contingency table. Fisher's exact test was then used to measure the magnitude of spatial separations between two types of compartments. Among the 15 selected mouse chromosomes (chr 1 to chr 14 and chr X), we found that the compartment label (A or B) of topological domains is significantly correlated with the spatial location of these domains relative to the fitted plane (on the left side or on the right side) in 14 chromosomes in both HindIII sample and NcoI sample (Table S7). As shown in Figure 3A, topological domains with the same compartment label tend to locate on the same side of the structure, consistent with their interaction frequencies, and the observation that compartment B tends to be associated with nuclear membrane [37], [38].

We further study how genomic and epigenetic features are distributed spatially in the whole chromosome model. Similar to the previous analysis for compartment labels (A or B), we conducted the same analysis for each of the eleven genomic and epigenetic features in consideration (Table S7). We used 33rd and 67th percentiles as the thresholds and divided all the 1,835 topological domains in chr 1 to chr 14 and chr X into three groups: domains with low value, with median value, and with high value of a particular feature. For each 3D chromosomal structure BACH predicted, we fitted a plane through domains with median value of the feature using the least square method. Next, we used the Fisher's exact test p-value to measure the magnitude of association between the group label (low value group or high value group) and spatial location of topological domains relative to the fitted plane (on the left side or on the right side). Table S7 lists the number of chromosomes with significant spatial separation patterns for each genomic and epigenetic feature in both HindIII sample and NcoI sample (threshold for Fisher's exact test p-value is 0.05). We observed that the gene density, transcription elongation histone modification mark H3K36me3, repressive histone modification mark H3K27me3, promoter mark H3K4me3, RNA polymerase II, chromatin accessibility, DNA replication time, heterochromatin marks H3K9me3 and H4K20me3 and genome-nuclear lamina interaction of topological domains are significantly associated with the spatial location of topological domains relative to the fitted plane (on the left side or on the right side) among more than nine chromosomes (Table S7 and Figure 3B∼Figure 3L).

## Discussion

We have described BACH and BACH-MIX, two Bayesian statistical models, to study 3D chromosomal structures and structural variations of chromatins from the Hi-C data. The benefits of using a probabilistic approach are two-folds: first, rigorous statistical inference can be carried out to properly remove systematic biases and account for observational noise sources; second, sequencing depth variations can be explicitly modeled by Poisson distributions. Our results demonstrate that BACH is significantly more reproducible and achieves higher consistency with the FISH data than an existing algorithm (MCMC5C). Application of BACH to a recently published Hi-C dataset from the mouse ES cells reveals interesting structural properties of mammalian chromosomes. Specifically, we found that geometric shapes of topological domains are strongly correlated with several genomic and epigenetic features. For example, gene rich, actively transcribed, accessible and early replicated chromatins tend to be more elongated than gene poor, lowly transcribed, inaccessible and late replicated chromatins. Furthermore, by using a variant BACH-MIX algorithm, we found that structural variations of a chromatin are also correlated with several genomic and epigenetic features.

There are several issues that we have not addressed in this paper, such as biophysical properties of chromatin fiber [39], [40] and the low sequencing depth of inter-chromosomal chromatin interactions. In principle, biophysical properties can be accommodated directly in our Bayesian model as spatial constraints through an informative prior on spatial distances. With more experimental work and additional data, the BACH and BACH-MIX algorithms can be applied to study the spatial arrangement of multiple chromosomes simultaneously. With the rapid accumulation of high throughput genome-wide chromatin interaction data, the BACH and BACH-MIX algorithms could be valuable tools for understanding higher order chromatin architecture of mammalian cells.

## Methods

### The BACH algorithm

To reconstruct the underlying consensus 3D chromosomal structure, we develop the following probabilistic model, similar to the “beads-on-a-string” model (Figure S9) that has been used extensively in chemistry. The genomic region of interest is divided into 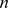 consecutive, disjoint loci of equal size 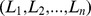, and each locus 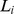 is represented by a bead in the 3D space, whose location is given by the Cartesian coordinates 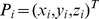. The Euclidean distance 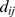 between loci 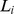 and 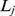 represents the average spatial distance between these two loci 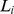 and 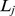:
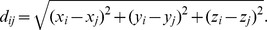
Under this representation, reconstructing the 3D chromosomal structure is equivalent to placing these beads in the 3D space, i.e., specifying the Cartesian coordinates 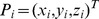 of these loci.

Let 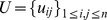 be the 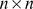 symmetric contact matrix generated by the Hi-C experiment, where each entry 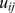 represents the number of paired-end reads spanning two loci 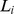 and 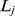. The variations of 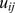 can be explained by several factors. Lieberman-Aiden et al. [9] first reported the negative association between the number of paired-end reads spanning two loci (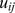) and the corresponding spatial distance (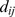). Recently, Yaffe and Tanay [17] identified some systematic biases, including restriction enzyme cutting frequencies, GC content and sequence uniqueness of fragment ends, which substantially affect Hi-C data. Taking all these unique features into consideration, we propose the following Poisson model.

Let 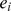, 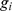 and 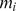 represent the number of fragment ends within locus 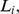 the mean GC content of fragment ends within locus 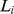, and the mean mappability score of fragment ends within locus 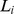, respectively [17]. We assume that the off-diagonal count 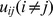 in the contact matrix 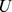 follows a Poisson distribution with rate 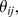 where:
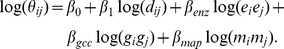
In this model, 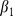 measures the magnitude of negative association 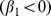 between 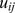 and 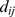. 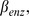 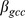 and 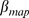 are the coefficients for the enzyme effect, GC content effect and mappability effect, respectively. The link function in this Poisson model provides the relationship between the linear predictors (i.e., the spatial distance, the number of fragment ends, the mean GC content of fragment ends and the mean mappability score of fragment ends) and the mean of Poisson distribution, which can be used to translate the number of paired-end reads spanning two loci into the average spatial distance between them.

Let 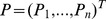 (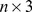 matrix) represent the Cartesian coordinates of the 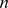 loci of interest, and let 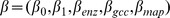 be the collection of all nuisance parameters. The joint likelihood is of the form:
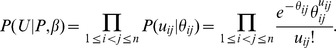
We adopt a fully Bayesian approach with non-informative priors for all model parameters, and obtain the following joint posterior distribution:
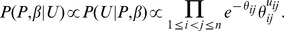
Due to the high dimensionality of the parameter space, designing an efficient computational tool to draw samples from 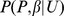 is essential for the statistical inference of our model. To achieve this goal, we propose a three-stage statistical inference procedure (Figure S10). First we assign initial values for the nuisance parameters using a Poisson regression approach [41]. We then use sequential importance sampling (SIS) [42] to generate an initial 3D chromosomal structure. At the end, we apply Gibbs sampler [43] with hybrid Monte Carlo [21], [44] and adaptive rejection sampling (ARS) [45] to further refine the 3D chromosomal structure and the nuisance parameters. More details of three-stage statistical inference procedure can be found in Text S1.

### HD ratio

Let 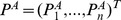 represent the Cartesian coordinates of the genomic region 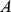 with 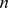 loci, where 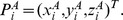 First we shift the genomic region 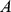 such that its weight center is at the original point 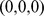. We then conduct the principle component analysis on the 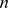 by 3 matrix 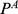, and rotate matrix 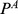 to matrix 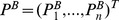, 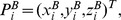 such that the x-axis is the direction of the first principle component (the one explains most variability) and the y-axis and the z-axis are the directions of the second and the third principle components, respectively. We use a cylinder to approximate the 3D chromosomal structure of the genomic region 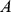. The height of the cylinder is defined as the difference between the 90% quantile of 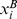 and the 10% quantile of 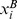. The radius of the cylinder is defined as two times the median of 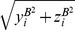 We further define HD ratio of the genomic region 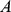 as the ratio between the height of the cylinder and the diameter of the cylinder, and then normalized by the size of genomic region 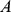. By the definition, genomic regions with higher HD ratios are more elongated.

### The BACH-MIX algorithm

We propose the BACH-MIX algorithm to study the spatial arrangement of two adjacent genomic regions. Here we assume that each genomic region exhibits a unique consensus 3D chromosomal structure, but the spatial arrangement of two adjacent genomic regions has certain level of flexibility, and varies according to a probabilistic distribution. More precisely, let 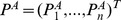 and 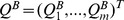 represent the 3D chromosomal structures of two adjacent genomic region 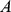 and 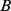, respectively, where 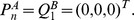 The spatial arrangement of the genomic region 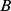 with respect to the genomic region 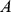 is determined by three Euler angles [23] 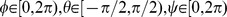 and an index 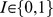 for mirror symmetry. Let 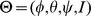 be the collection of these four parameters, and define the rotation matrix 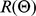 and the mirror symmetry matrix 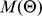 as:
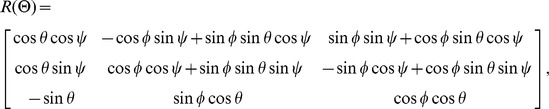


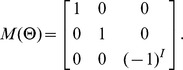
The spatial arrangement of the genomic region 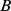 with respect to the genomic region 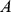, denoted 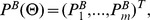 can be calculated by:
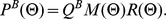
Therefore, each 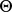 corresponds to a 3D chromosomal structure of two adjacent genomic regions 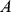 and 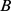, and a probabilistic distribution 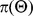 defines a mixture of distinct spatial arrangements between the two adjacent genomic regions 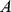 and 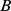. To further simplify the statistical inference problem on 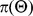, we discretize the four dimensional space of 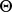, and use a multinomial distribution 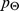 to approximate 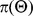.

Let 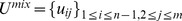 be the 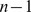 by 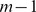 dimensional contact matrix for inter-region chromatin interactions, where 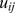 represent the number of reads spanning the 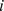 th locus in the genomic region 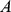 and the 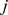 th locus in the genomic region 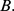 We assume that 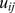 follow Poisson distribution with rate 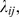 where
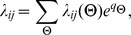


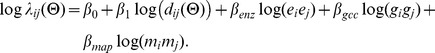
Here 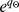 is the Poisson offset for the spatial arrangement 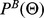, which is proportional to 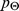. The statistical inference problem on the multinomial distribution 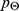 is equivalent to infer 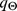. 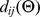 is the spatial distance between the 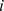 th locus in the genomic region 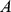 and the 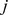 th locus in the genomic region 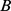 with rotation matrix 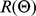 and mirror symmetry matrix 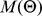. 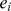, 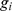 and 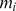 are local genomic features which follow the previous definitions. The joint likelihood is of form:
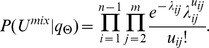
We adopt a fully Bayesian approach with non-informative priors for all model parameters, and obtain the following joint posterior distribution:
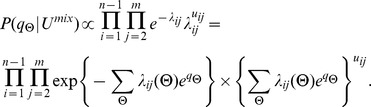
We use hybrid Monte Carlo to jointly update the parameters 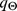 (Figure S10). The first order partial derivatives with respect to 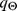 is of the form:
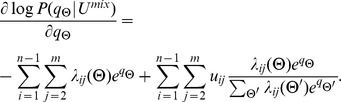



### Normalized Root Mean Square Deviation (RMSD)

Assuming 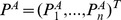 and 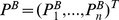 are the Cartesian coordinates of two genomic regions 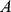 and 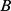, respectively, where 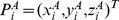 and 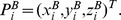 We first remove the scaling effect by a regression procedure. Let 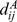 and 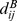 be the Euclidean distance between loci 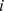 and 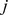 in 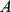 and 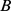, respectively. We regress 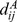 against 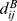 and obtain the slope 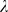. Define 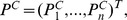 where 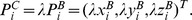 Assume 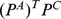 has the singular value decomposition 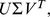 and then the optimal rotation matrix 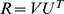 can minimize the sum of square error 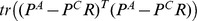 [46]. The normalized RMSD is defined as:
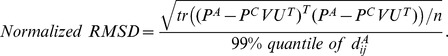
Empirically, normalized RMSD less than 0.1 indicates high similarity, normalized RMSD between 0.1 and 0.2 indicates moderate similarity, while normalized RMSD larger than 0.2 indicates low similarity.

### Model implementation

Under the default setting of BACH, we draw 100 3D chromosomal structures at each step of sequential importance sampling. We further enrich each 3D chromosomal structure ten times when we implement the rejection control technique. In the Gibbs sampler of BACH and BACH-MIX, we run three parallel chains with 5,000 MCMC iterations in each chain. The first 1,000 samples are dropped as the burn-in stage, and then every 50^th^ sample in the last 4,000 samples are used for the posterior inference. We use the Gelman-Rubin statistic [43] to measure the mixing of three parallel chains. Empirically, the Gelman-Rubin statistics less than 1.1 indicates that three parallel chains converge to the same posterior distribution.

### Computation time

The computation time of BACH and BACH-MIX depends on the number of MCMC iterations and the number of loci in the genomic region of interest. All MCMC calculations are conducted on computing nodes in Harvard Linux cluster “Odyssey”, each with dual Xeon E5410 2.3 GHz quad core processors and 32 GB RAM. Under the default setting, BACH takes 81 seconds to predict a 3D chromosomal structure with 25 loci; BACH-MIX takes 8 minutes to predict the proportion of 104 distinct 3D chromosomal structures for two 13 loci adjacent genomic regions. The computation time increases almost quadratically with the number of loci in the genomic region of interest.

### URL

BACH and BACH-MIX can be freely downloaded at http://www.fas.harvard.edu/~junliu/BACH/.

## Supporting Information

Figure S1 **Local genomic features of the mouse genome at 40 KB resolution.** (**A**) Distribution of the number of fragment end within each 40 KB locus in the HindIII sample. (**B**) Distribution of the GC content within each 40 KB locus in the HindIII sample. (**C**) Distribution of the mappability score within each 40 KB locus in the HindIII sample. (**D**) Distribution of the number of fragment end within each 40 KB locus in the NcoI sample. (**E**) Distribution of the GC content within each 40 KB locus in the NcoI sample. (**F**) Distribution of the mappability score within each 40 KB locus in the NcoI sample.
(DOCX)

Figure S2 **Comparison between the spatial distances BACH predicted with the FISH distances using the high resolution Hi-C dataset on mouse embryonic stem cells.** (**A**) 40 KB resolution Hi-C contact matrices of four domains in the HindIII sample and the NcoI sample. (**B**) The 3D chromosomal structures BACH predicted. In domain 1, red, blue, green and purple dots represent gene GCR, gene Lnp, gene Evx2 and gene Hoxd3, respectively. In domain 2, red and blue dots represent gene Rcn1 and gene 1550J22, respectively. In domain 3, red and blue dots represent gene Il9r and gene Hbq1, respectively. In domain 4, red, blue and green dots represent gene Calcoco2, gene Hoxb9 and gene Hoxb1, respectively. (**C**) Comparison between the spatial distances BACH predicted in the HindIII sample with FISH distances. Each dot represents the posterior mean, and each bar represents the 95% credible interval. We treat the FISH distances as the gold standard, and use a linear regression procedure to adjust the scale parameter. (**D**) Comparison between the spatial distances BACH predicted in the NcoI sample with FISH distances. Each dot represents the posterior mean, and each bar represents the 95% credible interval. We treat the FISH distances as the gold standard, and use a linear regression procedure to adjust the scale parameter.
(DOCX)

Figure S3 **The structural variations of chromatin at the domain center region and at the domain boundary region.** (**A**) The number of 3D chromosomal structures with proportion larger than certain threshold (10%, 5% and 1%) in the HindIII sample. (**B**) The number of 3D chromosomal structures with proportion larger than certain threshold (10%, 5% and 1%) in the NcoI sample.
(DOCX)

Figure S4 **Simulation studies for the BACH algorithm when the input Hi-C contact matrix is simulated from a mixture population.** Black: RMSD(A, B), red: RMSD(S1, A), blue: RMSD(S1, B), green: RMSD(S2, A), yellow: RMSD(S2, B), purple: RMSD(S1, S2). (**A**) Distribution of six RMSDs across 100 simulated datasets, when the mixture proportion of the dominant sub-population is 50%. Black line represents the 5% quantile of RMSD calculated from the empirical distribution RMSD(A, B). (**B**) Distribution of six RMSDs across 100 simulated datasets, when the mixture proportion of the dominant sub-population is 60%. Black line represents the 5% quantile of RMSD calculated from the empirical distribution RMSD(A, B). (**C**) Distribution of six RMSDs across 100 simulated datasets, when the mixture proportion of the dominant sub-population is 70%. Black line represents the 5% quantile of RMSD calculated from the empirical distribution RMSD(A, B). (**D**) Distribution of six RMSDs across 100 simulated datasets, when the mixture proportion of the dominant sub-population is 80%. Black line represents the 5% quantile of RMSD calculated from the empirical distribution RMSD(A, B). (**E**) Distribution of six RMSDs across 100 simulated datasets, when the mixture proportion of the dominant sub-population is 90%. Black line represents the 5% quantile of RMSD calculated from the empirical distribution RMSD(A, B).
(DOCX)

Figure S5
**The alignment of two 3D chromosomal structures BACH predicted in the two stages,** 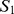 **and** 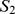 **, from 20 mouse chromosomes in both HindIII sample and NcoI sample.** Red lines represent the first BACH prediction 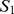. Blue lines represent the second BACH prediction 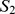. (**A**) The HindIII sample (**B**) The NcoI sample.(DOCX)

Figure S6 **The empirical distributions of RMSD for 20 mouse chromosomes with different lengths.** We generated two structures with the same size of each chromosome from the random walk scheme, and calculate the RMSD between them. We repeated this procedure 1,000 times for each chromosome to get the empirical distribution of RMSD, which is represented by a boxplot in Figure S6. The empirical distributions of RMSD for different chromosomes are similar, which are independent of chromosome size.
(DOCX)

Figure S7 **Comparison of reproducibility between BACH, BACH-SUB (a modified BACH algorithm without bias correction) and MCMC5C using the high resolution Hi-C dataset on mouse embryonic stem cells.** We focus on long chromosomes (chr 1 to chr 14 and chr X). (**A**) 3D chromosomal structures predicted by BACH using the mouse Hi-C data. Red lines and blue lines represent the HindIII sample and the NcoI sample, respectively. (**B**) 3D chromosomal structures predicted by BACH-SUB using the mouse Hi-C data. Red lines and blue lines represent the HindIII sample and the NcoI sample, respectively. (**C**) 3D chromosomal structures predicted by MCMC5C using the mouse Hi-C data. Red lines and blue lines represent the HindIII sample and the NcoI sample, respectively. (**D**) The normalized RMSDs of 3D chromosomal structures predicted from the HindIII sample and the NcoI sample, using BACH, BACH-SUB and MCMC5C. BACH achieved significantly higher reproducibility than MCMC5C (paired t-test p-value = 1.4e-7). BACH-SUB also achieved significantly higher reproducibility than MCMC5C (paired t-test p-value = 0.0465).
(DOCX)

Figure S8
**The local alignment of two 3D chromosomal structures BACH predicted in the two stages,** 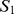 **and** 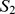 **, from 20 mouse chromosomes in both HindIII sample and NcoI sample.** (**A**) The local alignment results in the HindIII sample. (**B**) The local alignment results in the NcoI sample. We used a sliding window of ten domains to scan along each chromosome. For each possible position of the window, we aligned the two local structures from 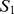 and 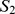 and calculated the RMSD between them. Thus, a series of RMSDs were obtained for a chromosome, each for one possible position of the sliding window. We summarized these RMSDs generated from each chromosome into a boxplot. We used the empirical distribution of the RMSD between two structures of ten loci generated from the random walk scheme as the reference for similarity evaluation. The red line represents the 5% lower quantile of the reference distribution. We observed that the median of RMSDs between 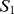 and 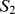 (black line in the middle of each box) have tail probabilities less than 0.05 in all 20 chromosomes. Therefore, 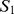 and 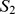 align well locally at the window size of ten domains. (**C**) The local alignment results measured by the median of the RMSDs in the HindIII sample. (**D**) The local alignment results measured by the median of the RMSDs in the NcoI sample. To be conservative, we used a different reference distribution. Instead of using two structures of ten loci, we generated two structures with the same size of each chromosome from the random walk scheme, conducted local alignment for them via the same sliding window strategy (window size is ten), and reported the median of the series of RMSDs obtained from this way. We repeated this procedure 1,000 times for each chromosome to get the empirical distribution of the median of the RMSDs, which is represented by a boxplot in Figure S8C and Figure S8D. The red dots represent the median of RMSDs obtained from 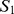 and 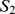 for different chromosomes. We observed that all red dots are located below the boxplots, indicating that 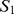 and 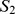 still align well locally measured by the median of the RMSDs.(DOCX)

Figure S9 **The “beads-on-a-string” model: an illustration of the 3D chromosomal structure with five loci.** The lengths of solid lines and dashed lines represent the spatial distances between two adjacent loci and two non-adjacent loci, respectively.
(DOCX)

Figure S10 **The flow chart of the BACH and BACH-MIX algorithm.**
(DOCX)

Figure S11 **Simulation study for the BACH algorithm.** (**A**) The hypothetical 3D chromosomal structure generated from a random walk scheme (red lines) and the posterior mode of the BACH predicted 3D chromosomal structure (white lines). (**B**) The trace plot of log likelihood of three parallel chains in 5,000 MCMC iterations. Chain 3 achieves the highest log likelihood among three parallel chains. (**C**) ACF plot of the log likelihood of the chain 3.
(DOCX)

Figure S12
**Simulation study for the BACH-MIX algorithm.** (**A**) The BACH predicted 3D chromosomal structure for the human chromosome 22 in a human lymphoblastic cell line with restriction enzyme HindIII. We divide the whole chromosome into two genomic regions: genomic region 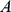 (red dots and lines) and genomic region 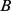 (white dots and lines). (**B**) The trace plot of log likelihood of three parallel chains in 5,000 MCMC iterations. Chain 3 achieves the highest log likelihood among three parallel chains. (**C**) ACF plot of the log likelihood of the chain 3. (**D**) The posterior distribution of 12 3D chromosomal structures.(DOCX)

Table S1 **Pearson correlation coefficients between HD ratios and genomic and epigenetic features.**
(DOCX)

Table S2 **The value and the rank of genomic and epigenetic features for a more elongated domain (chromosome 18, 33,960,000∼34,960,000, in the HindIII sample) and a less elongated domain (chromosome 7, 62,040,000∼63,040,000, in the HindIII sample).**
(DOCX)

Table S3 **The number of structures with proportion larger than 10% and 1%.**
(DOCX)

Table S4 **The structural variations of chromatin correlate with genetic and epigenetic features.** (**A**) In the HindIII sample, the structural variations correlate with genetic and epigenetic features. (**B**) In the NcoI sample, the structural variations correlate with genetic and epigenetic features.
(DOCX)

Table S5 **The mean of six RMSDs across 100 simulated datasets and the number of RMSDs below 5% quantile with different mixture proportions.** The 5% quantile of RMSD is calculated from the empirical distribution of RMSD, which is the empirical distribution of 100 RMSD(A, B).
(DOCX)

Table S6
**Applying the two-step procedure to the real Hi-C data, treat each topological domain as an individual unit.** The RMSD between two 3D chromosomal structures BACH predicted in the two stages, 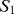 and 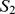, from 20 mouse chromosomes in both HindIII sample and NcoI sample. The tail probabilities less than 0.05 are highlighted in bold font.(DOCX)

Table S7 **Fisher's exact test to quantify the magnitude of spatial separations of genomic and epigenetic features.** We focus on long chromosomes (chr 1 to chr 14 and chr X). Each number represents the number of chromosome with significant spatial separation pattern. The p-value threshold is 0.05.
(DOCX)

Table S8 **Eleven FISH probes used in a study of the mESC (supplementary reference [10] in Text S1** **).**
(DOCX)

Table S9 **The normalized FISH distances between six probe pairs.**
(DOCX)

Table S10 **The annotations of four topological domains containing eleven FISH probes.**
(DOCX)

Table S11
**Posterior mean and 95% credible interval for parameters in the simulation study with single consensus 3D chromosomal structure.** We use the posterior samples in chain 3 (after burn-in and thin) for statistical inference. The true values for 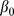, 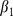, 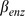, 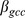 and 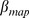 are 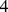, 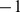, 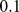, 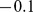 and 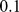, respectively.(DOCX)

Table S12
**The true value, posterior mean and 95% credible interval for the 12 dimensional multinomial distribution** 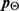 **used in the simulation study with multiple distinct 3D chromosomal structures.**
(DOCX)

Table S13
**Applying the two-step procedure to the zoomed-in real Hi-C data (equally split one topological domain into two sub-domains), treat each sub-domain as an individual unit.** The RMSD between two 3D chromosomal structures BACH predicted in the two stages, 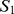 and 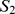, from 20 mouse chromosomes in both HindIII sample and NcoI sample. The tail probabilities < = 0.05 are highlighted in bold font.(DOCX)

Table S14
**Applying the two-step procedure to the zoomed-out real Hi-C data (combine two adjacent topological domains into one super-domain), treat each super-domain as an individual unit.** The RMSD between two 3D chromosomal structures BACH predicted in the two stages, 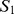 and 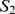, from 20 mouse chromosomes in both HindIII sample and NcoI sample. The tail probabilities < = 0.05 are highlighted in bold font.(DOCX)

Table S15 **The RMSD between the 3D chromosomal structure inferred from the zoomed-in Hi-C contact matrices and the 3D chromosomal structure inferred from the original Hi-C contact matrices.** The tail probabilities < = 0.05 are highlighted in bold font.
(DOCX)

Table S16 **The RMSD between the 3D chromosomal structures inferred from the zoomed-out Hi-C contact matrices and the 3D chromosomal structures inferred from the original Hi-C contact matrices.** The tail probabilities < = 0.05 are highlighted in bold font.
(DOCX)

Table S17
**Applying the two-step procedure to the subset of real Hi-C data (equally split one chromosome into two halves), treat each topological domain as an individual unit.** The RMSD between two 3D chromosomal structures BACH predicted in the two stages, 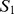 and 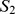, from 20 mouse chromosomes in both HindIII sample and NcoI sample. The tail probabilities < = 0.05 are highlighted in bold font.(DOCX)

Table S18 **The RMSD between the 3D chromosomal structures inferred from the subset of Hi-C contact matrices (equally split one chromosome into two halves) and the 3D chromosomal structures inferred from the original Hi-C contact matrices.** The tail probabilities < = 0.05 are highlighted in bold font.
(DOCX)

Text S1 **Description of the computational protocol [**10**].**
(DOCX)
