# Supplementary figures and images for: Bayesian Inference of Spatial Organizations of Chromosomes
Source: PLoS Comput Biol. 2013 Jan 31;9(1):e1002893. doi: 10.1371/journal.pcbi.1002893 (PMC3561073; doi:10.1371/journal.pcbi.1002893)

**A. B. C.**


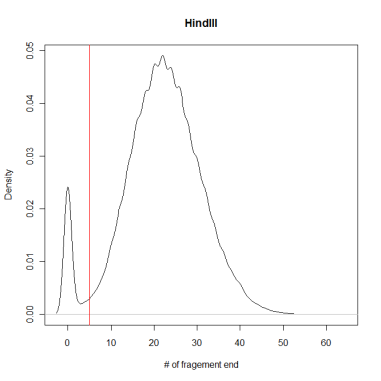

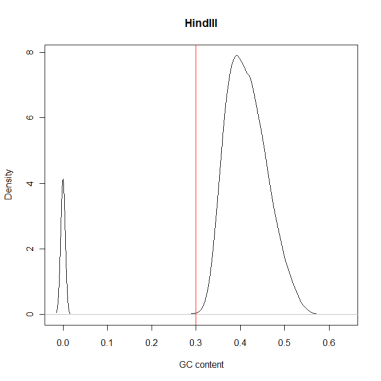

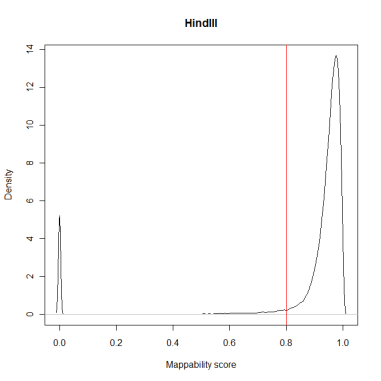


**D. E. F.**


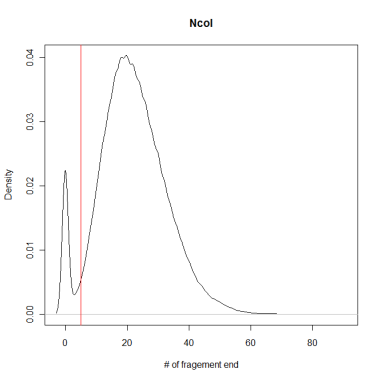

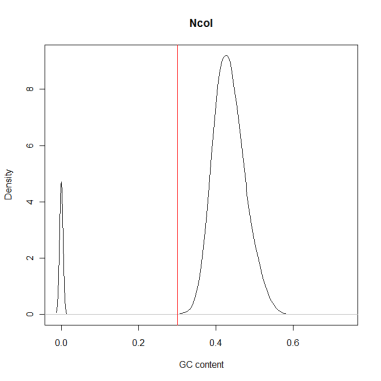

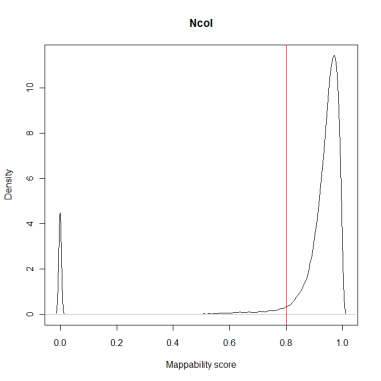

Supplement: Figure S1 — Local genomic features of the mouse genome at 40 KB resolution. (A) Distribution of the number of fragment end within each 40 KB locus in the HindIII sample. (B) Distribution of the GC content within each 40 KB locus in the HindIII sample. (C) Distribution of the mappability score within each 40 KB locus in the HindIII sample. (D) Distribution of the number of fragment end within each 40 KB locus in the NcoI sample. (E) Distribution of the GC content within each 40 KB locus in the NcoI sample. (F) Distribution of the mappability score within each 40 KB locus in the NcoI sample. (DOCX) [file pcbi.1002893.s001.docx]

**A. B.**

**
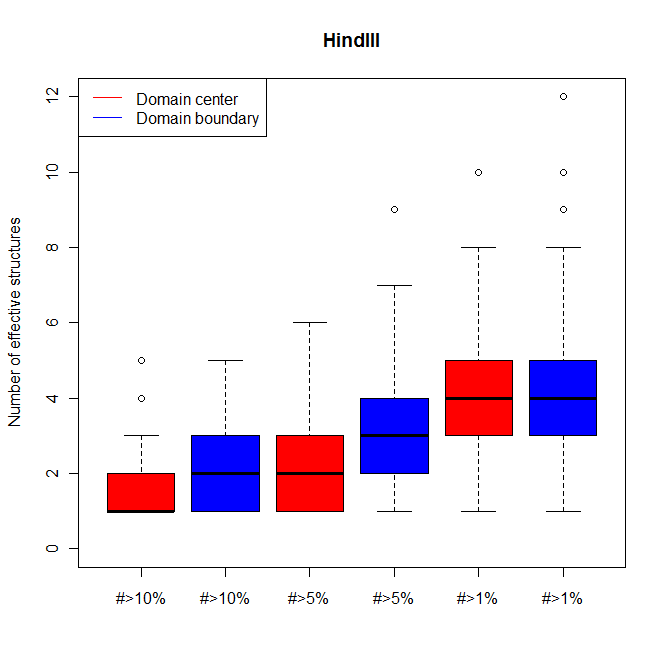

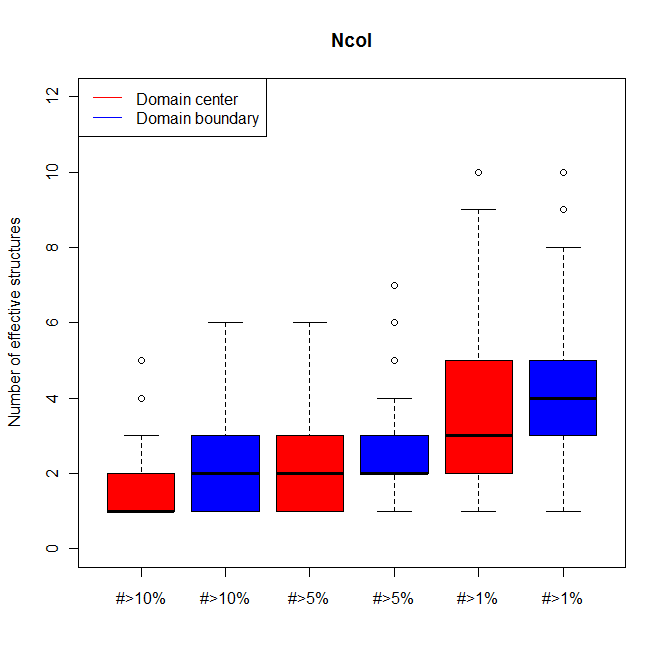
**

Supplement: Figure S3 — The structural variations of chromatin at the domain center region and at the domain boundary region. (A) The number of 3D chromosomal structures with proportion larger than certain threshold (10%, 5% and 1%) in the HindIII sample. (B) The number of 3D chromosomal structures with proportion larger than certain threshold (10%, 5% and 1%) in the NcoI sample. (DOCX) [file pcbi.1002893.s003.docx]

**A. B. C.**

**
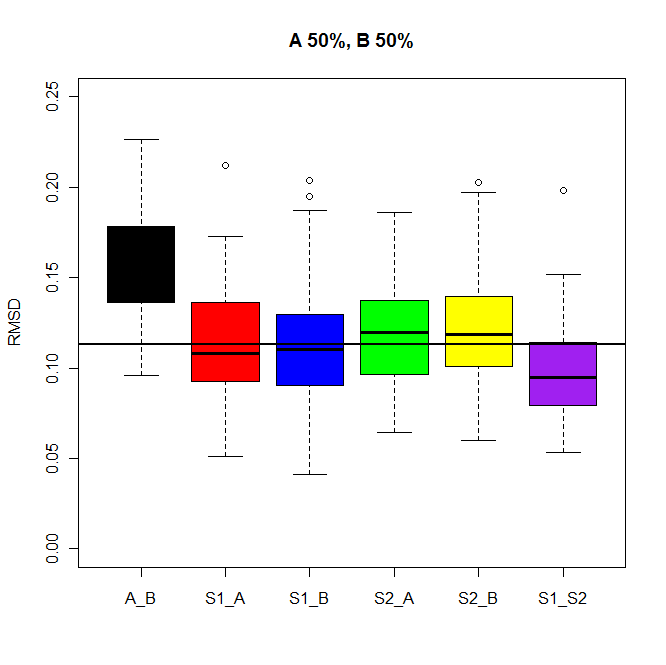

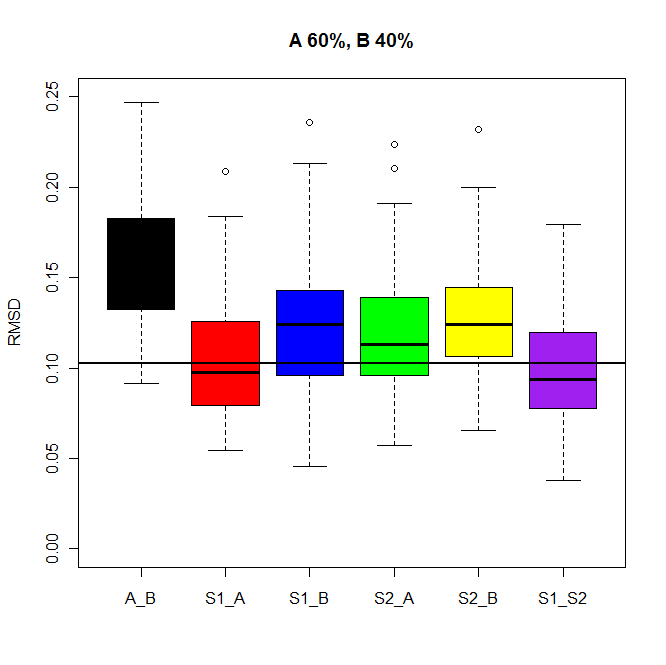

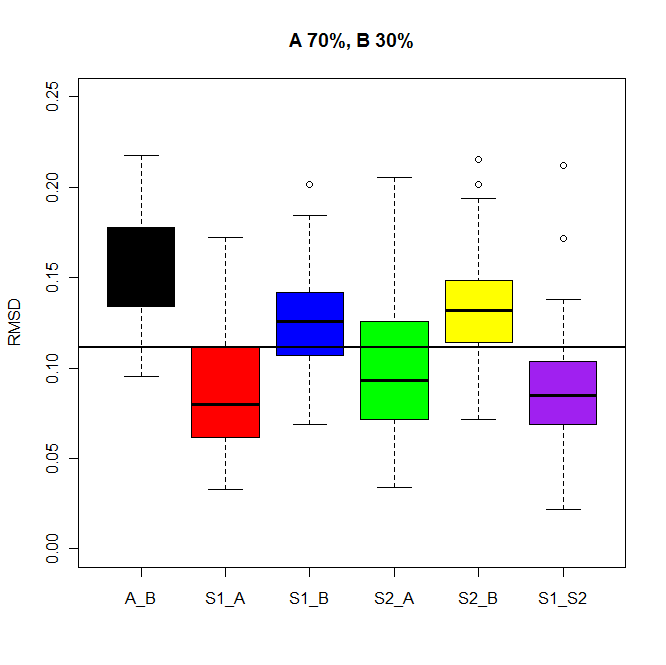
**

**D. E.**

**
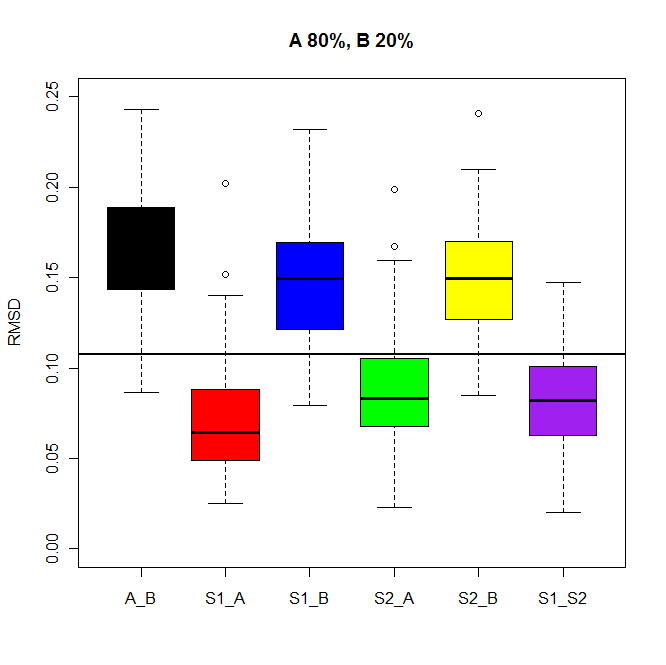

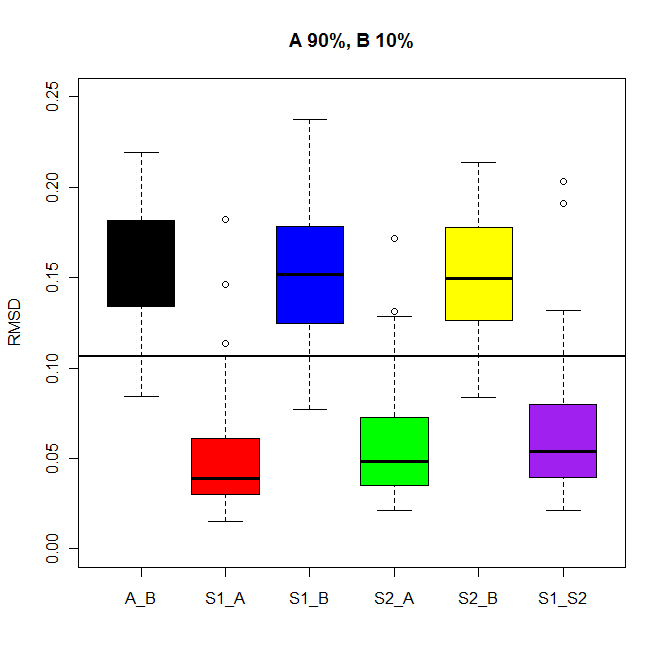
**

Supplement: Figure S4 — Simulation studies for the BACH algorithm when the input Hi-C contact matrix is simulated from a mixture population. Black: RMSD(A, B), red: RMSD(S1, A), blue: RMSD(S1, B), green: RMSD(S2, A), yellow: RMSD(S2, B), purple: RMSD(S1, S2). (A) Distribution of six RMSDs across 100 simulated datasets, when the mixture proportion of the dominant sub-population is 50%. Black line represents the 5% quantile of RMSD calculated from the empirical distribution RMSD(A, B). (B) Distribution of six RMSDs across 100 simulated datasets, when the mixture proportion of the dominant sub-population is 60%. Black line represents the 5% quantile of RMSD calculated from the empirical distribution RMSD(A, B). (C) Distribution of six RMSDs across 100 simulated datasets, when the mixture proportion of the dominant sub-population is 70%. Black line represents the 5% quantile of RMSD calculated from the empirical distribution RMSD(A, B). (D) Distribution of six RMSDs across 100 simulated datasets, when the mixture proportion of the dominant sub-population is 80%. Black line represents the 5% quantile of RMSD calculated from the empirical distribution RMSD(A, B). (E) Distribution of six RMSDs across 100 simulated datasets, when the mixture proportion of the dominant sub-population is 90%. Black line represents the 5% quantile of RMSD calculated from the empirical distribution RMSD(A, B). (DOCX) [file pcbi.1002893.s004.docx]

**
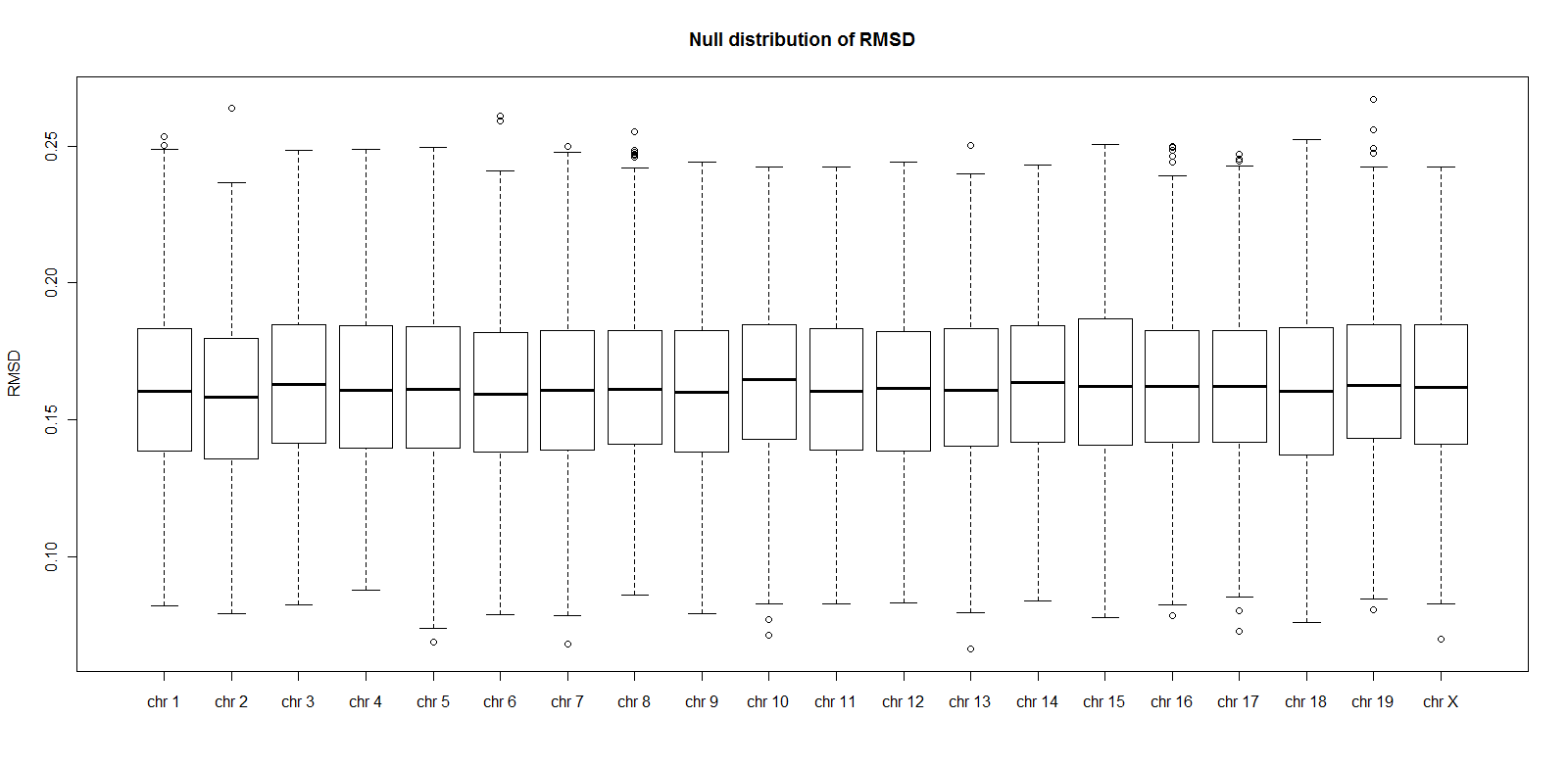
**

Supplement: Figure S6 — The empirical distributions of RMSD for 20 mouse chromosomes with different lengths. We generated two structures with the same size of each chromosome from the random walk scheme, and calculate the RMSD between them. We repeated this procedure 1,000 times for each chromosome to get the empirical distribution of RMSD, which is represented by a boxplot in Figure S6. The empirical distributions of RMSD for different chromosomes are similar, which are independent of chromosome size. (DOCX) [file pcbi.1002893.s006.docx]

**A.**

**
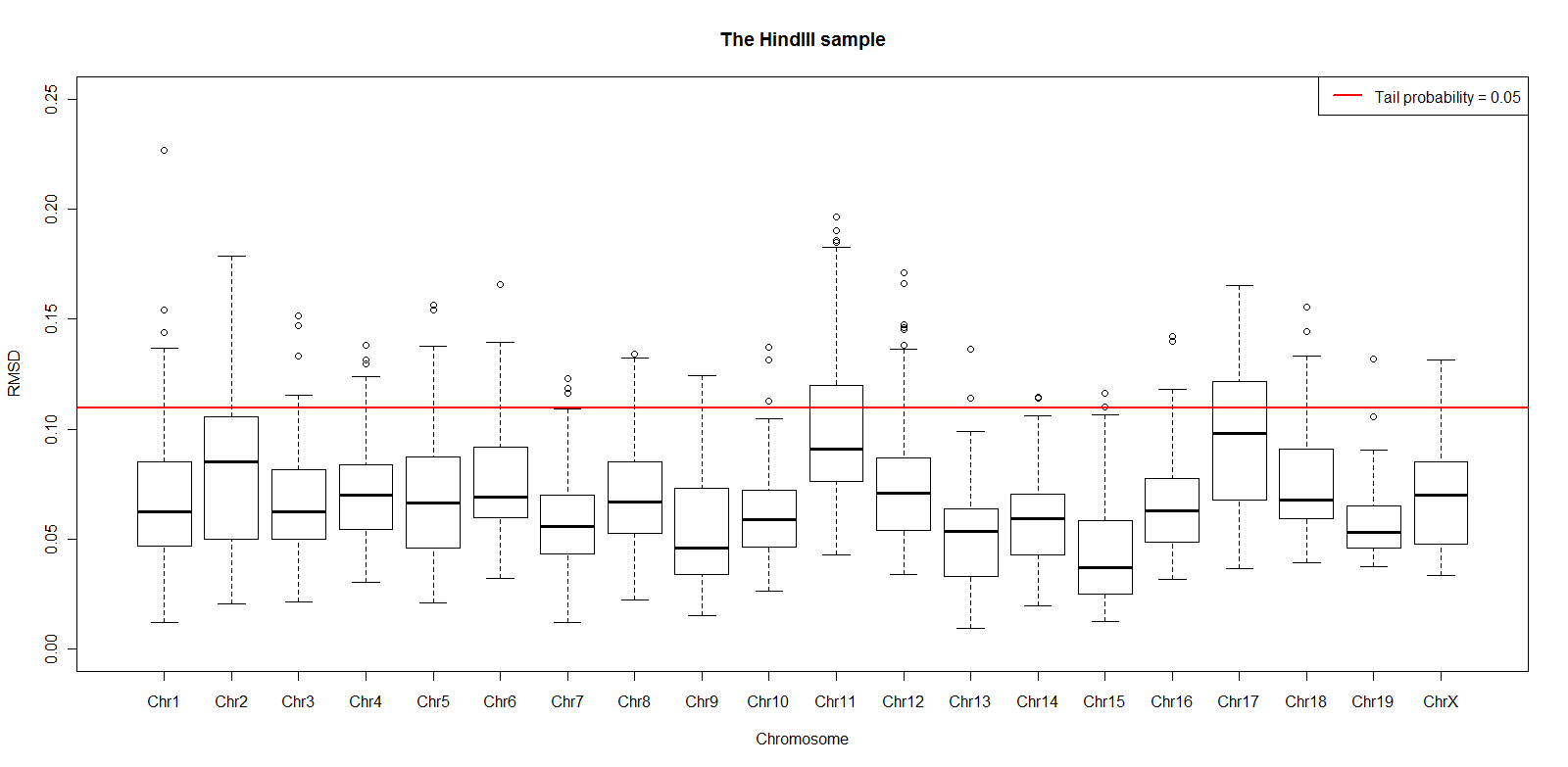
**

**B.**

**
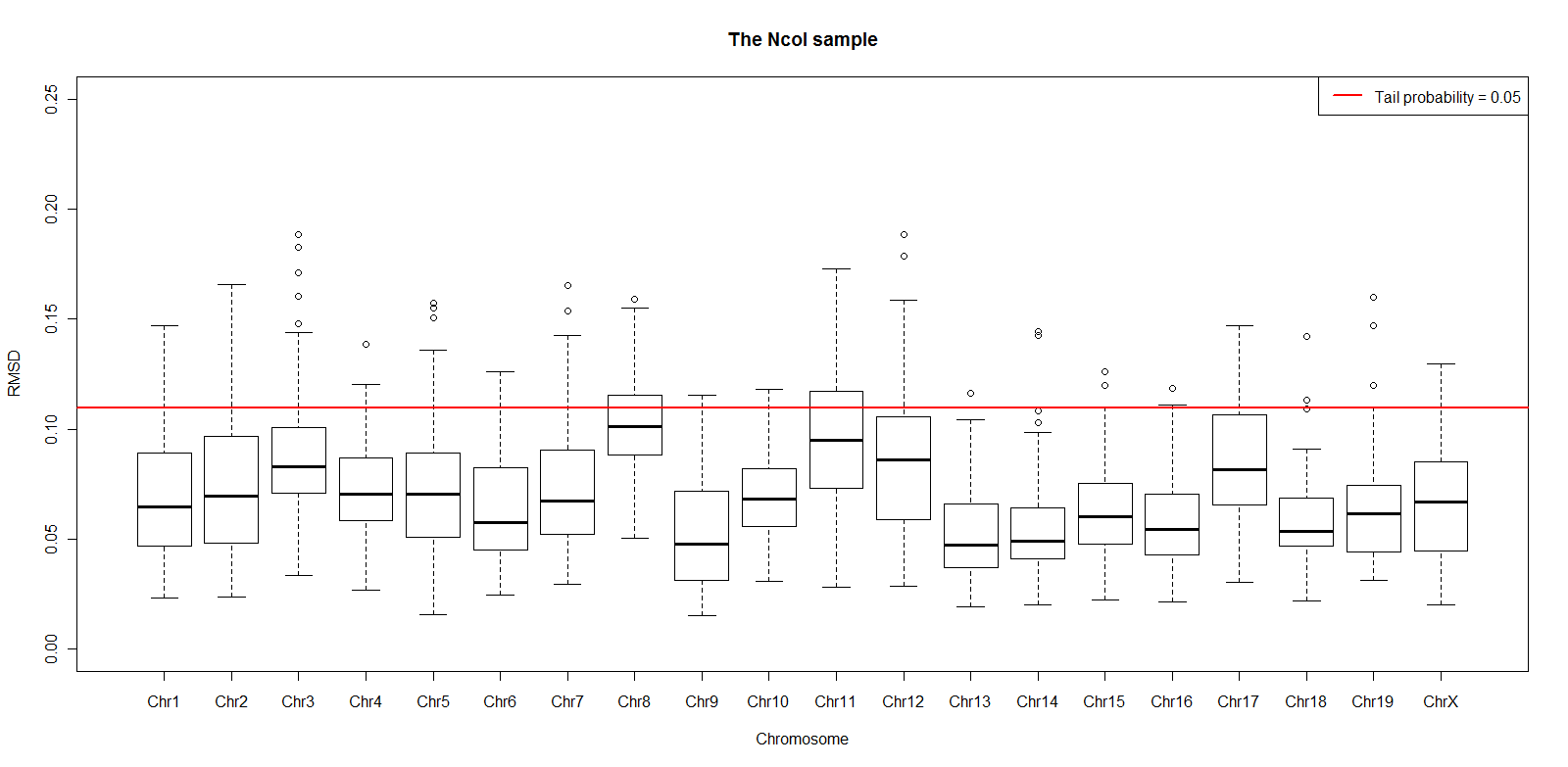
**

**C.**

**
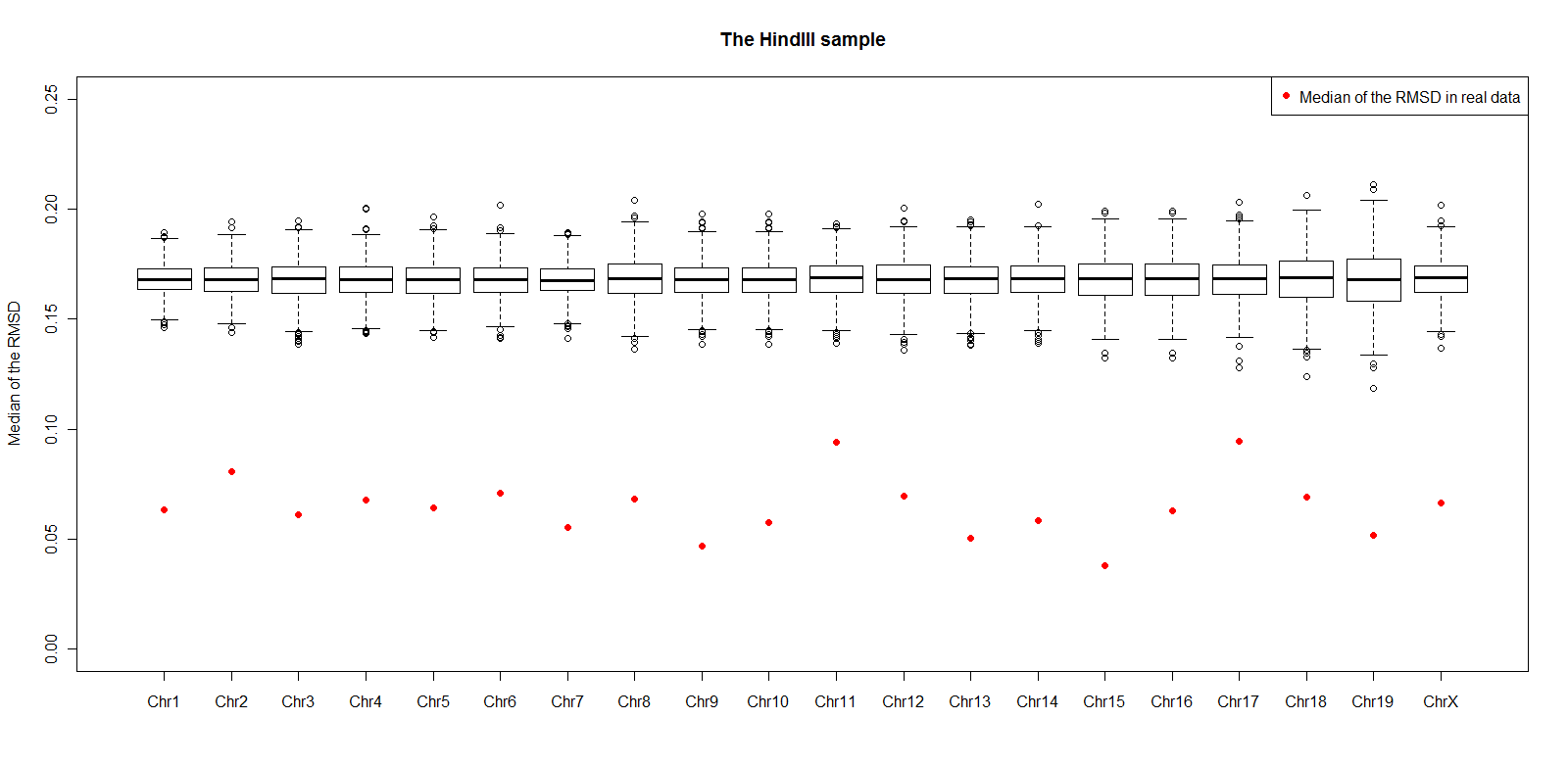
**

**D.**

**
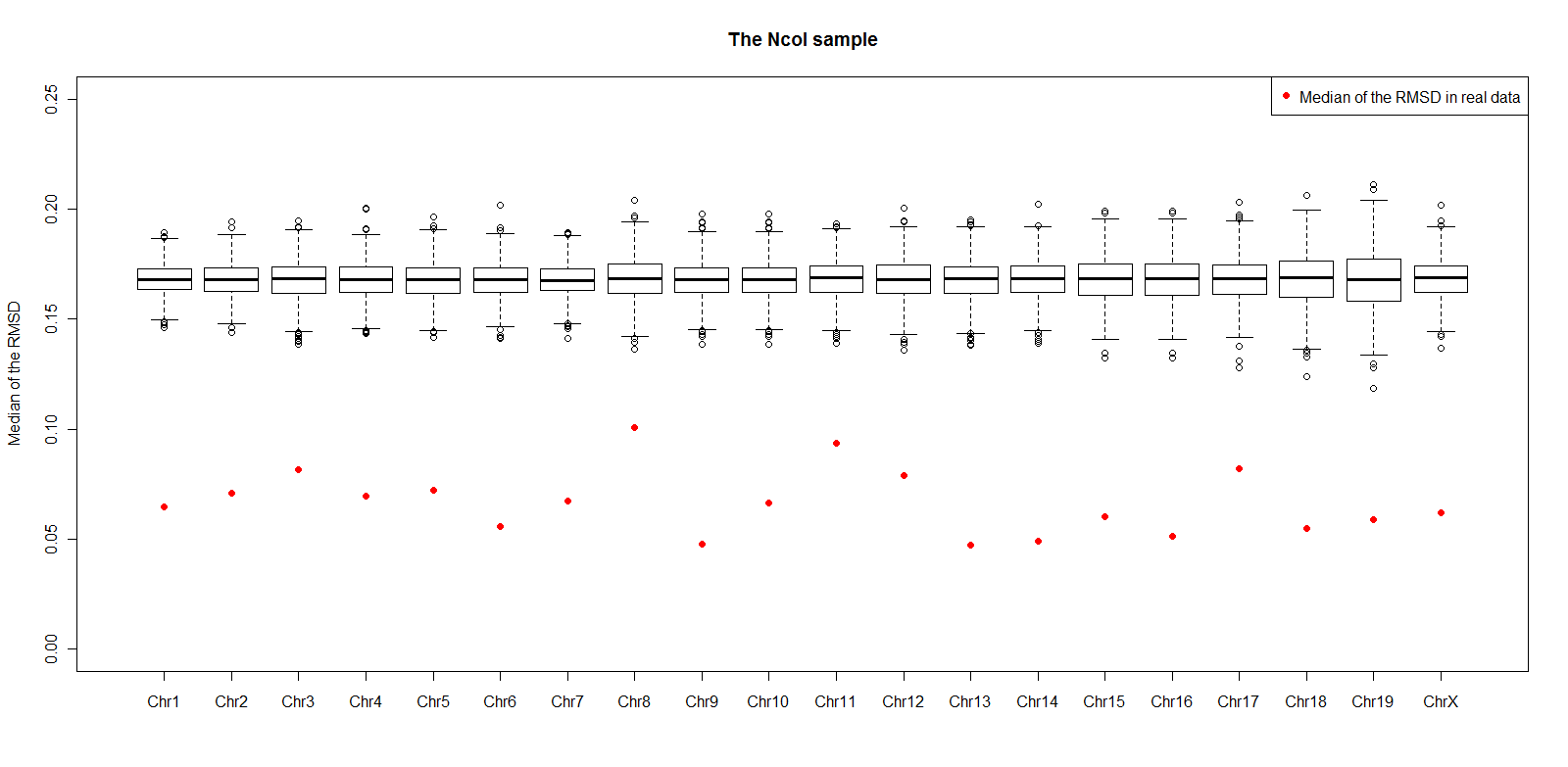
**

Supplement: Figure S8 — The local alignment of two 3D chromosomal structures BACH predicted in the two stages, and , from 20 mouse chromosomes in both HindIII sample and NcoI sample. (A) The local alignment results in the HindIII sample. (B) The local alignment results in the NcoI sample. We used a sliding window of ten domains to scan along each chromosome. For each possible position of the window, we aligned the two local structures from and and calculated the RMSD between them. Thus, a series of RMSDs were obtained for a chromosome, each for one possible position of the sliding window. We summarized these RMSDs generated from each chromosome into a boxplot. We used the empirical distribution of the RMSD between two structures of ten loci generated from the random walk scheme as the reference for similarity evaluation. The red line represents the 5% lower quantile of the reference distribution. We observed that the median of RMSDs between and (black line in the middle of each box) have tail probabilities less than 0.05 in all 20 chromosomes. Therefore, and align well locally at the window size of ten domains. (C) The local alignment results measured by the median of the RMSDs in the HindIII sample. (D) The local alignment results measured by the median of the RMSDs in the NcoI sample. To be conservative, we used a different reference distribution. Instead of using two structures of ten loci, we generated two structures with the same size of each chromosome from the random walk scheme, conducted local alignment for them via the same sliding window strategy (window size is ten), and reported the median of the series of RMSDs obtained from this way. We repeated this procedure 1,000 times for each chromosome to get the empirical distribution of the median of the RMSDs, which is represented by a boxplot in Figure S8C and Figure S8D. The red dots represent the median of RMSDs obtained from and for different chromosomes. We observed that all red dots are located below the boxplots, indicating t [file pcbi.1002893.s008.docx]

**
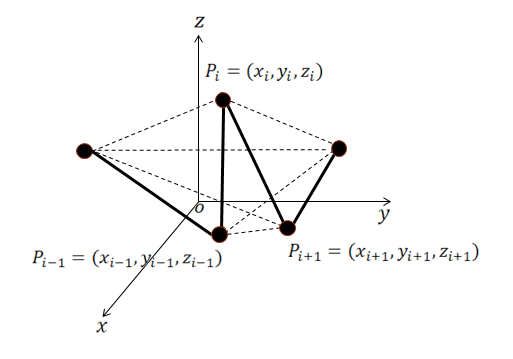
**

Supplement: Figure S9 — The “beads-on-a-string” model: an illustration of the 3D chromosomal structure with five loci. The lengths of solid lines and dashed lines represent the spatial distances between two adjacent loci and two non-adjacent loci, respectively. (DOCX) [file pcbi.1002893.s009.docx]

**A. B. C.**

**
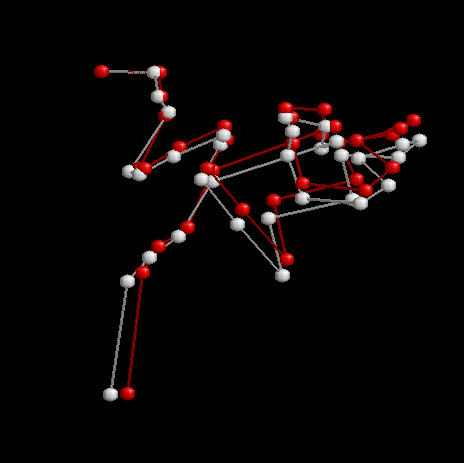

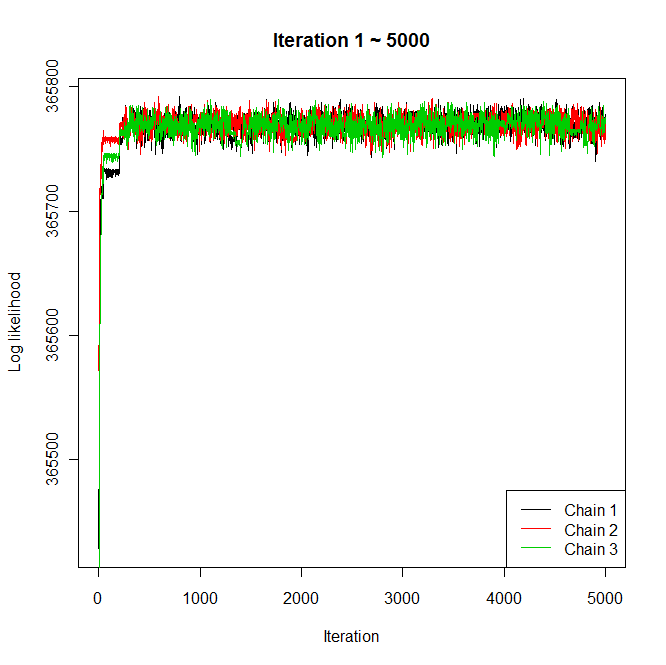

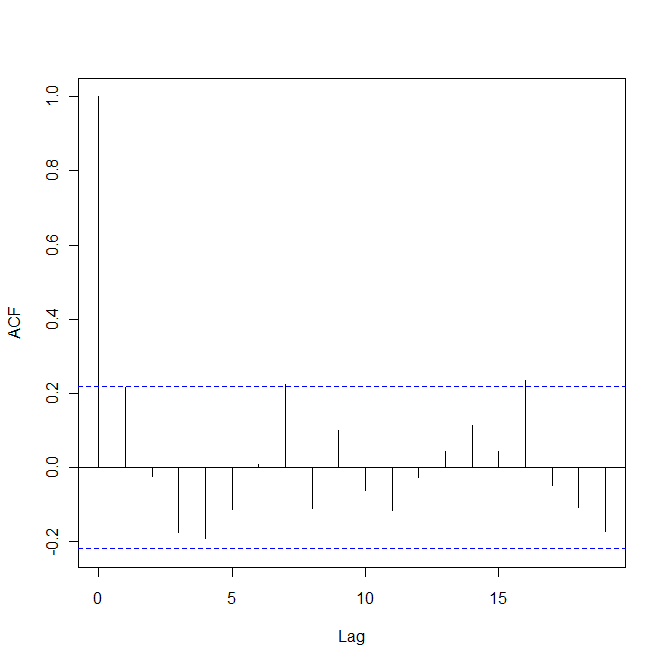
**

Supplement: Figure S11 — Simulation study for the BACH algorithm. (A) The hypothetical 3D chromosomal structure generated from a random walk scheme (red lines) and the posterior mode of the BACH predicted 3D chromosomal structure (white lines). (B) The trace plot of log likelihood of three parallel chains in 5,000 MCMC iterations. Chain 3 achieves the highest log likelihood among three parallel chains. (C) ACF plot of the log likelihood of the chain 3. (DOCX) [file pcbi.1002893.s011.docx]

**A. B. C.**

**
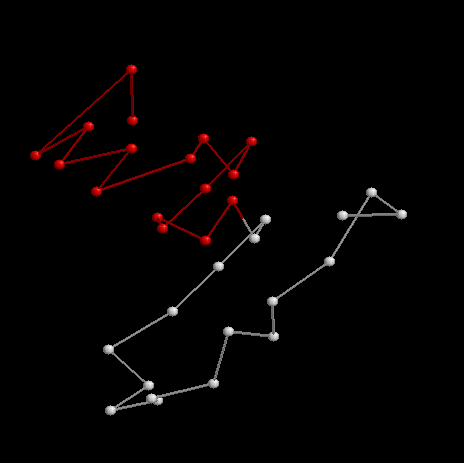

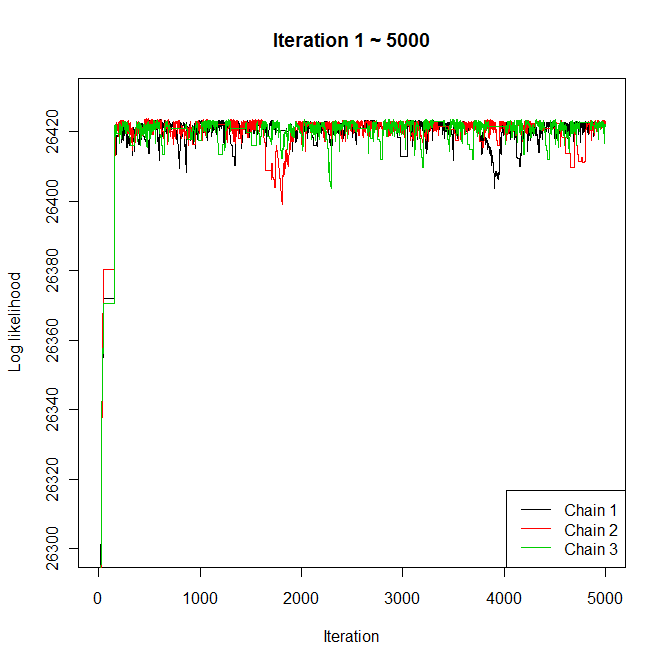

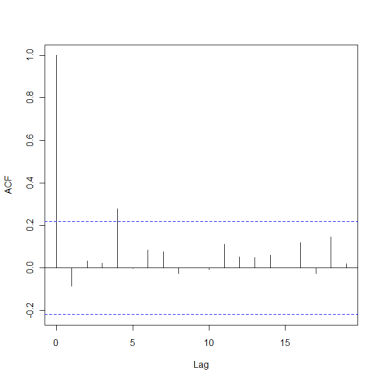
**

**D.**

**
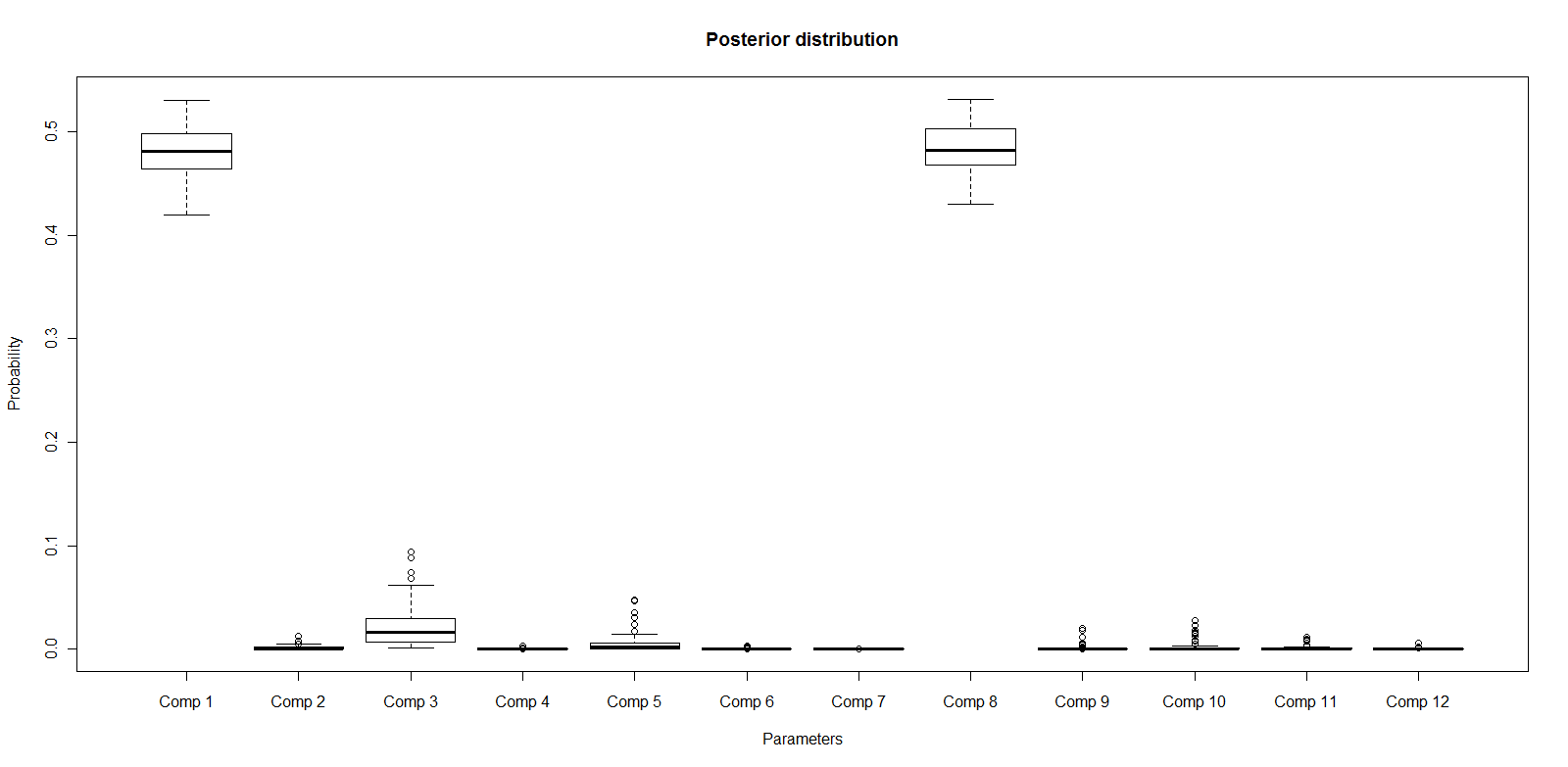
**

Supplement: Figure S12 — Simulation study for the BACH-MIX algorithm. (A) The BACH predicted 3D chromosomal structure for the human chromosome 22 in a human lymphoblastic cell line with restriction enzyme HindIII. We divide the whole chromosome into two genomic regions: genomic region (red dots and lines) and genomic region (white dots and lines). (B) The trace plot of log likelihood of three parallel chains in 5,000 MCMC iterations. Chain 3 achieves the highest log likelihood among three parallel chains. (C) ACF plot of the log likelihood of the chain 3. (D) The posterior distribution of 12 3D chromosomal structures. (DOCX) [file pcbi.1002893.s012.docx]
