# Supplementary material for: Bayesian Inference of Spatial Organizations of Chromosomes
Source: PLoS Comput Biol. 2013 Jan 31;9(1):e1002893. doi: 10.1371/journal.pcbi.1002893 (PMC3561073; doi:10.1371/journal.pcbi.1002893)
Supplement: Figure S2 — Comparison between the spatial distances BACH predicted with the FISH distances using the high resolution Hi-C dataset on mouse embryonic stem cells. (A) 40 KB resolution Hi-C contact matrices of four domains in the HindIII sample and the NcoI sample. (B) The 3D chromosomal structures BACH predicted. In domain 1, red, blue, green and purple dots represent gene GCR, gene Lnp, gene Evx2 and gene Hoxd3, respectively. In domain 2, red and blue dots represent gene Rcn1 and gene 1550J22, respectively. In domain 3, red and blue dots represent gene Il9r and gene Hbq1, respectively. In domain 4, red, blue and green dots represent gene Calcoco2, gene Hoxb9 and gene Hoxb1, respectively. (C) Comparison between the spatial distances BACH predicted in the HindIII sample with FISH distances. Each dot represents the posterior mean, and each bar represents the 95% credible interval. We treat the FISH distances as the gold standard, and use a linear regression procedure to adjust the scale parameter. (D) Comparison between the spatial distances BACH predicted in the NcoI sample with FISH distances. Each dot represents the posterior mean, and each bar represents the 95% credible interval. We treat the FISH distances as the gold standard, and use a linear regression procedure to adjust the scale parameter. (DOCX) [file pcbi.1002893.s002.docx]

**A.**

| Domain 1, HindIII  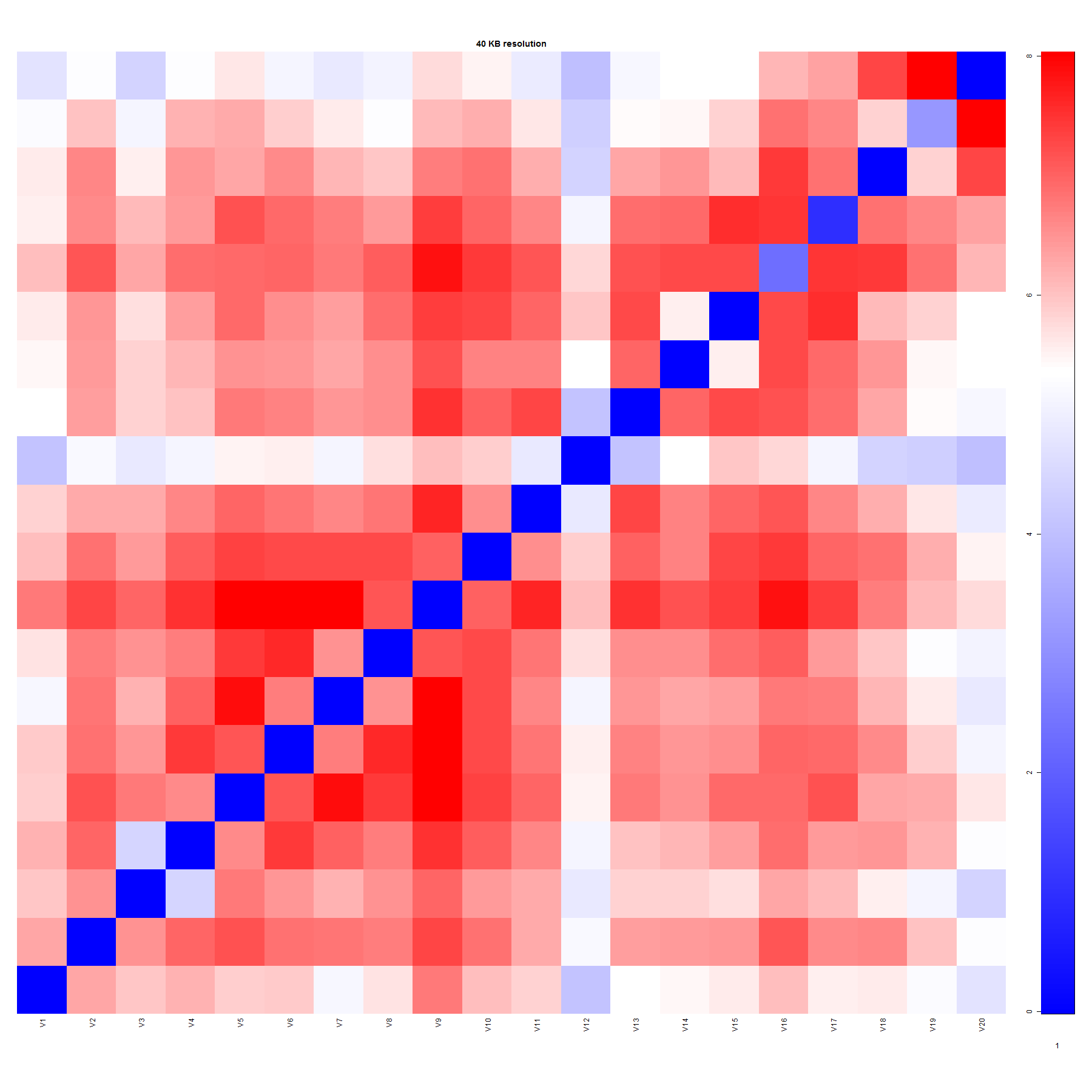 | Domain 2, HindIII  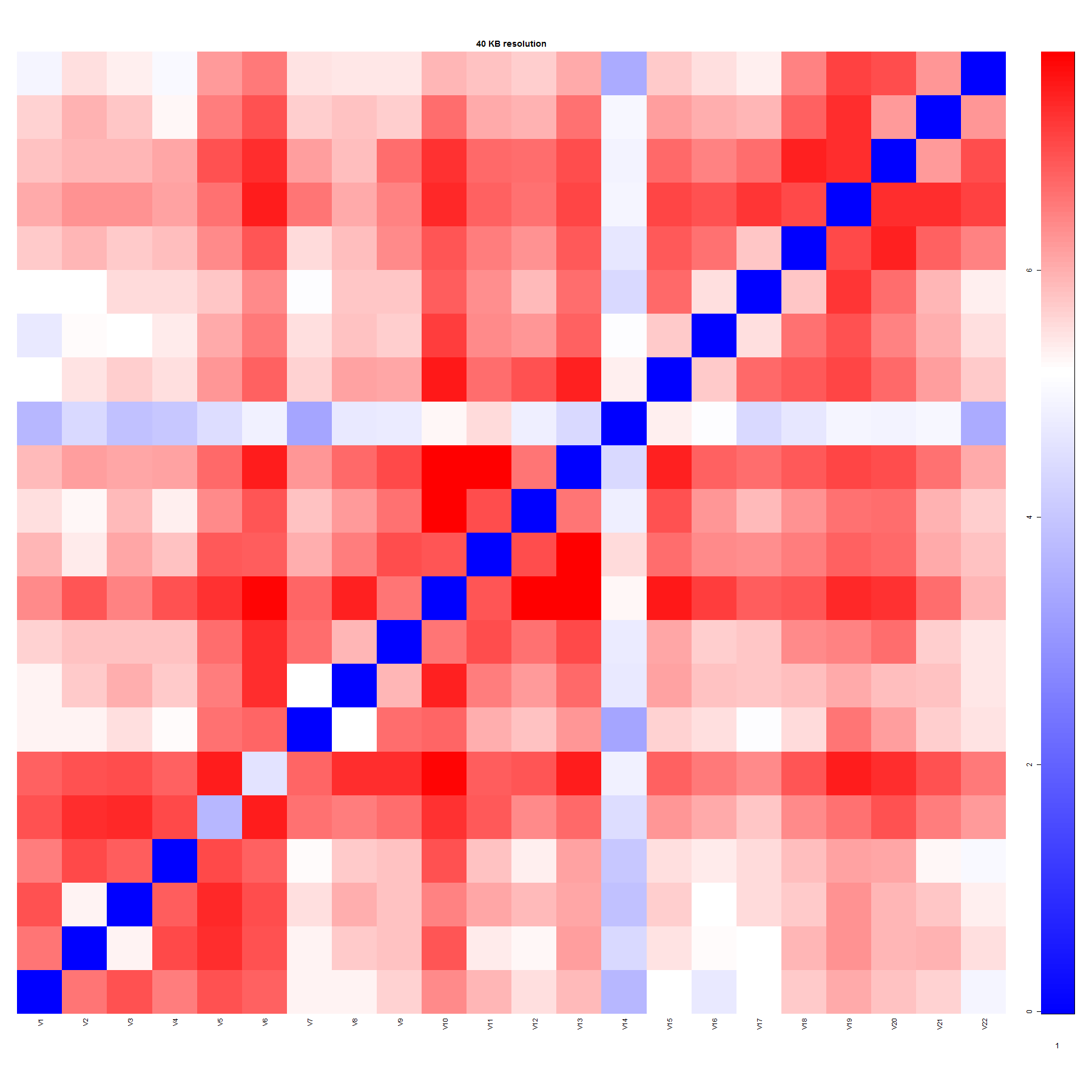 | Domain 3, HindIII  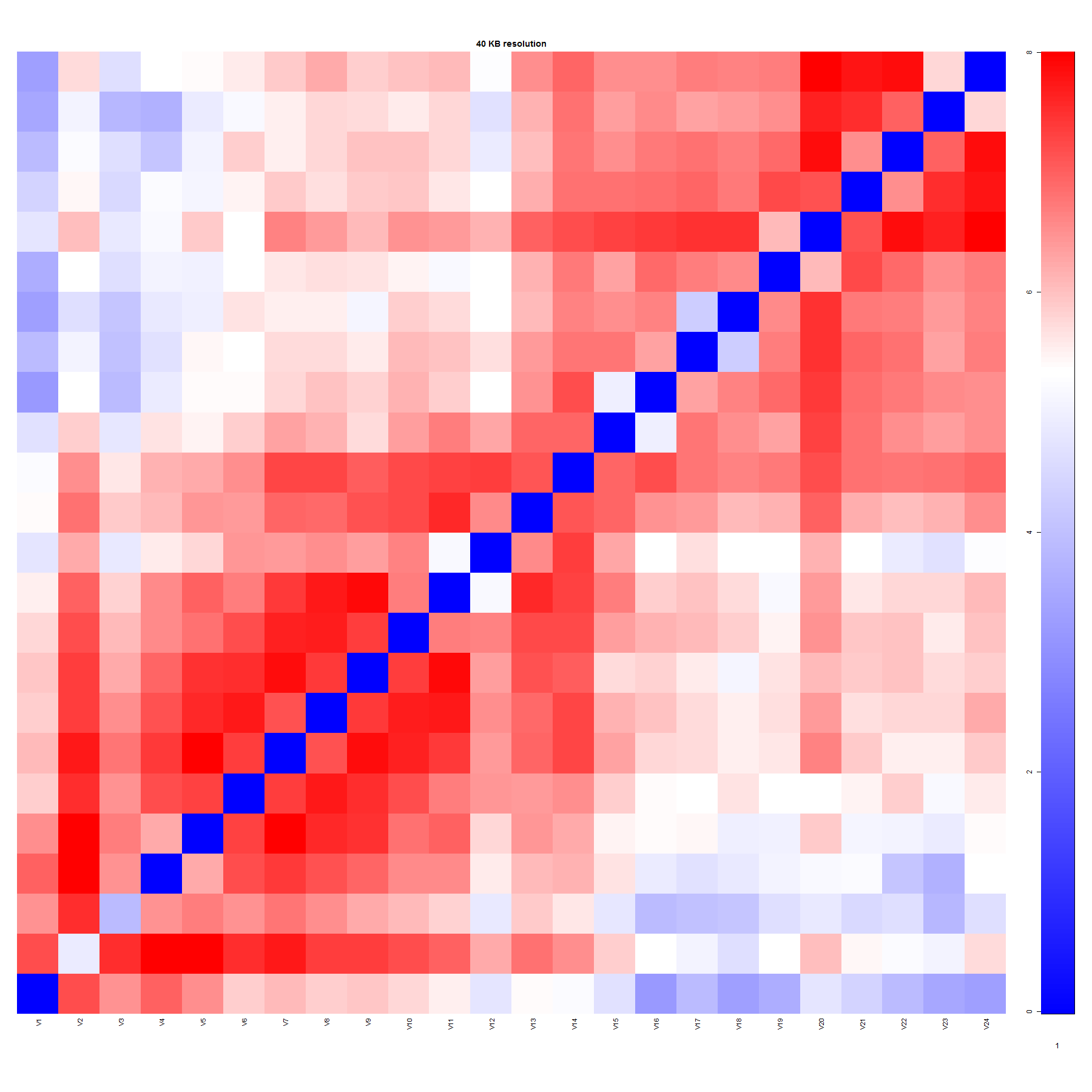 | Domain 4, HindIII  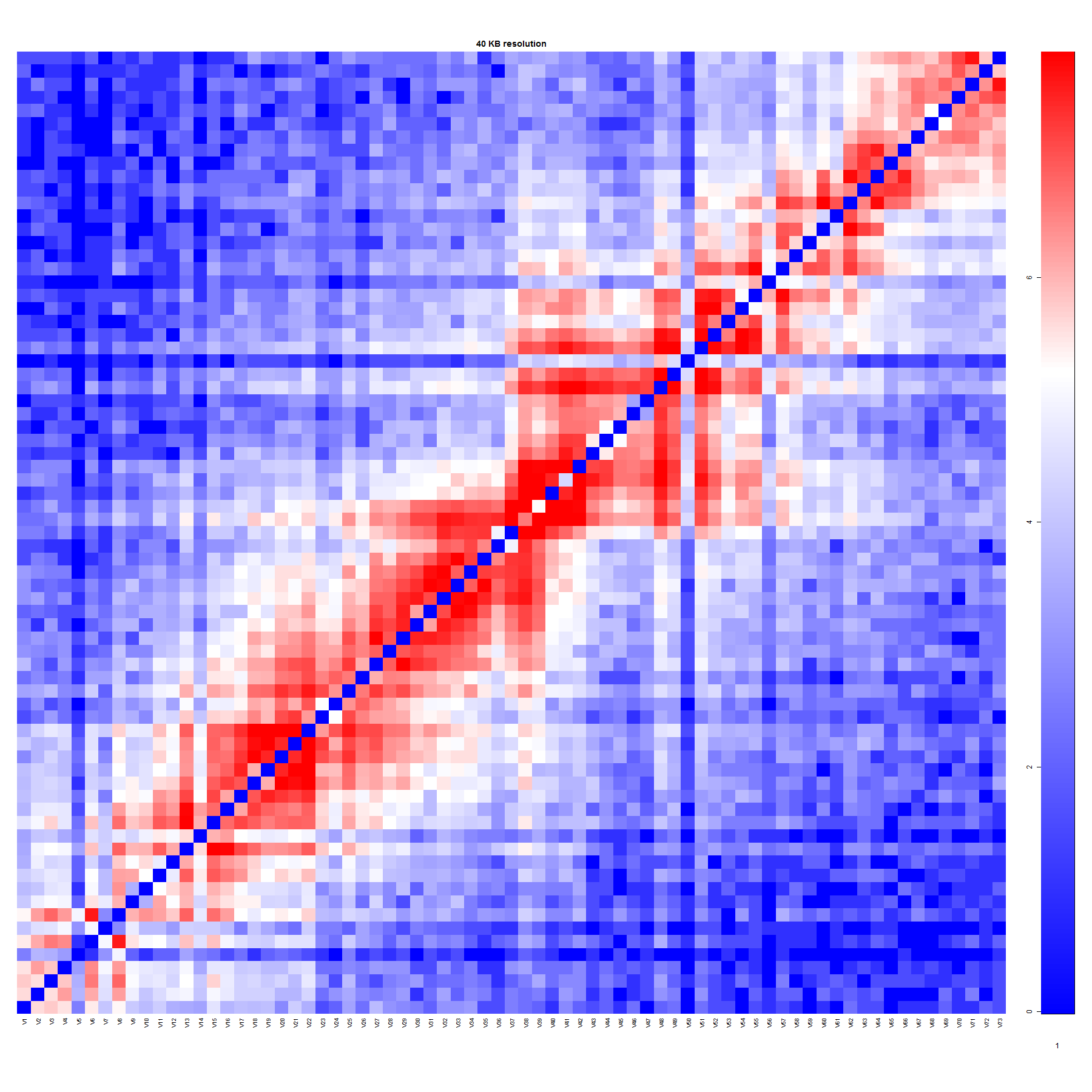 |
| --- | --- | --- | --- |
| Domain 1, NcoI  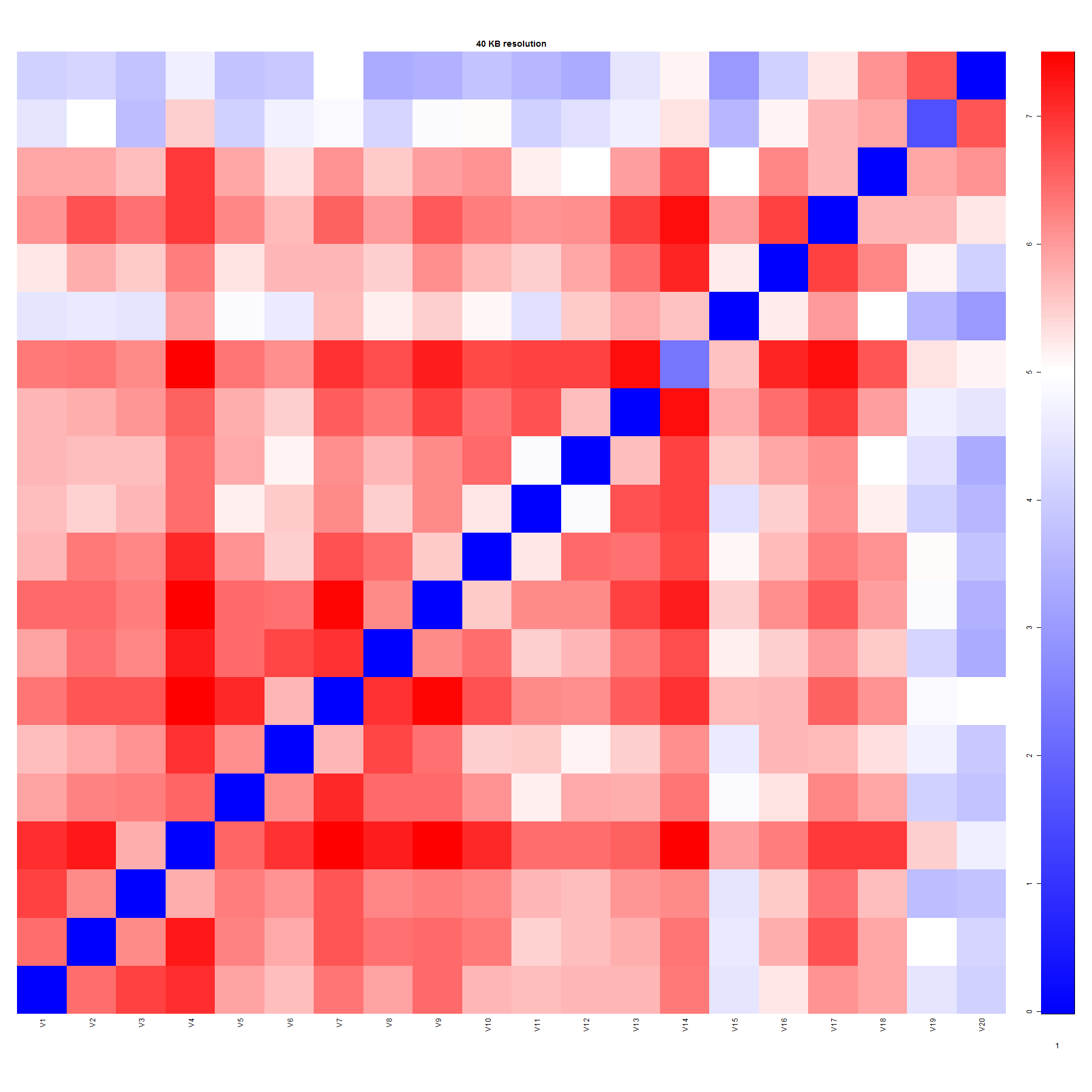 | Domain 2, NcoI  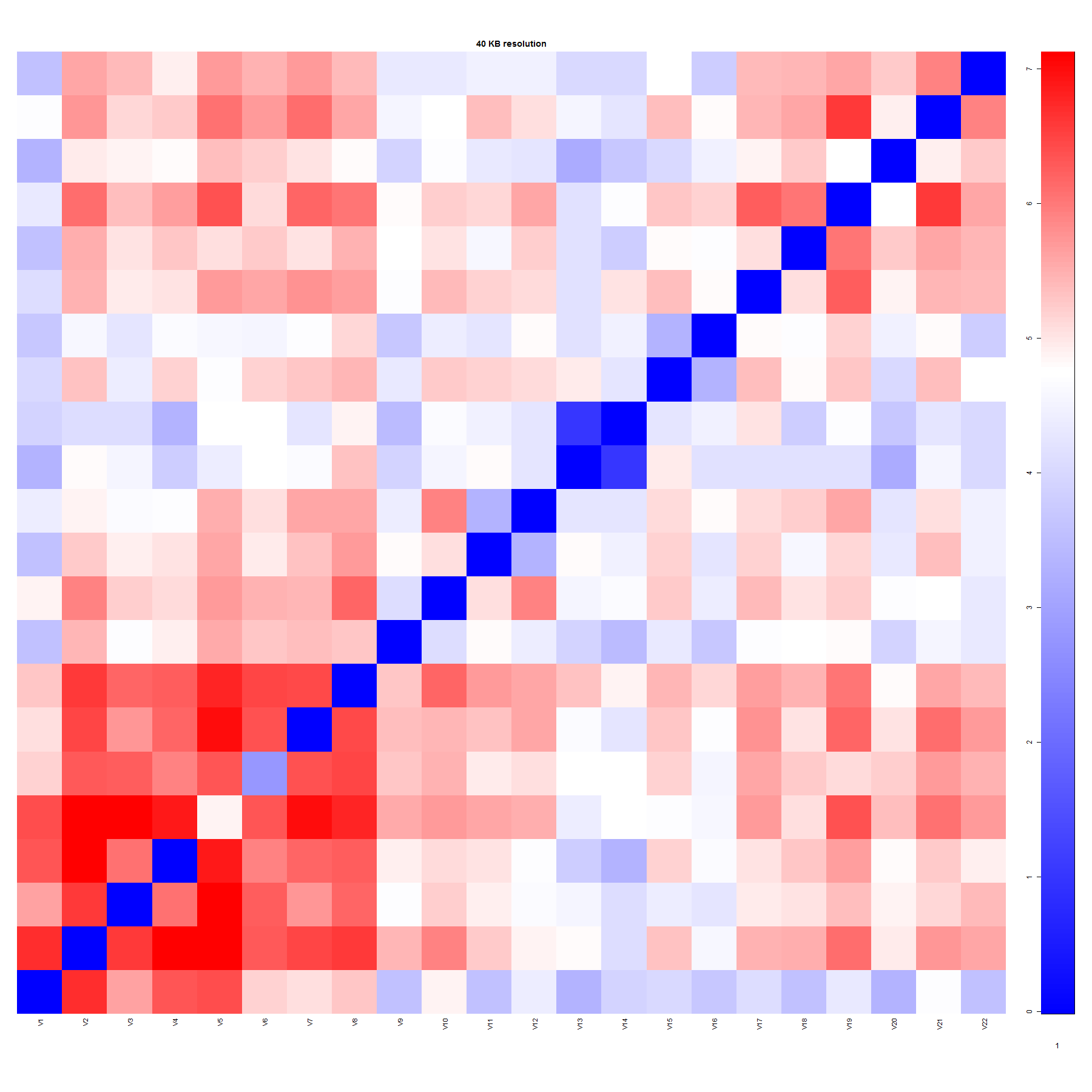 | Domain 3, NcoI  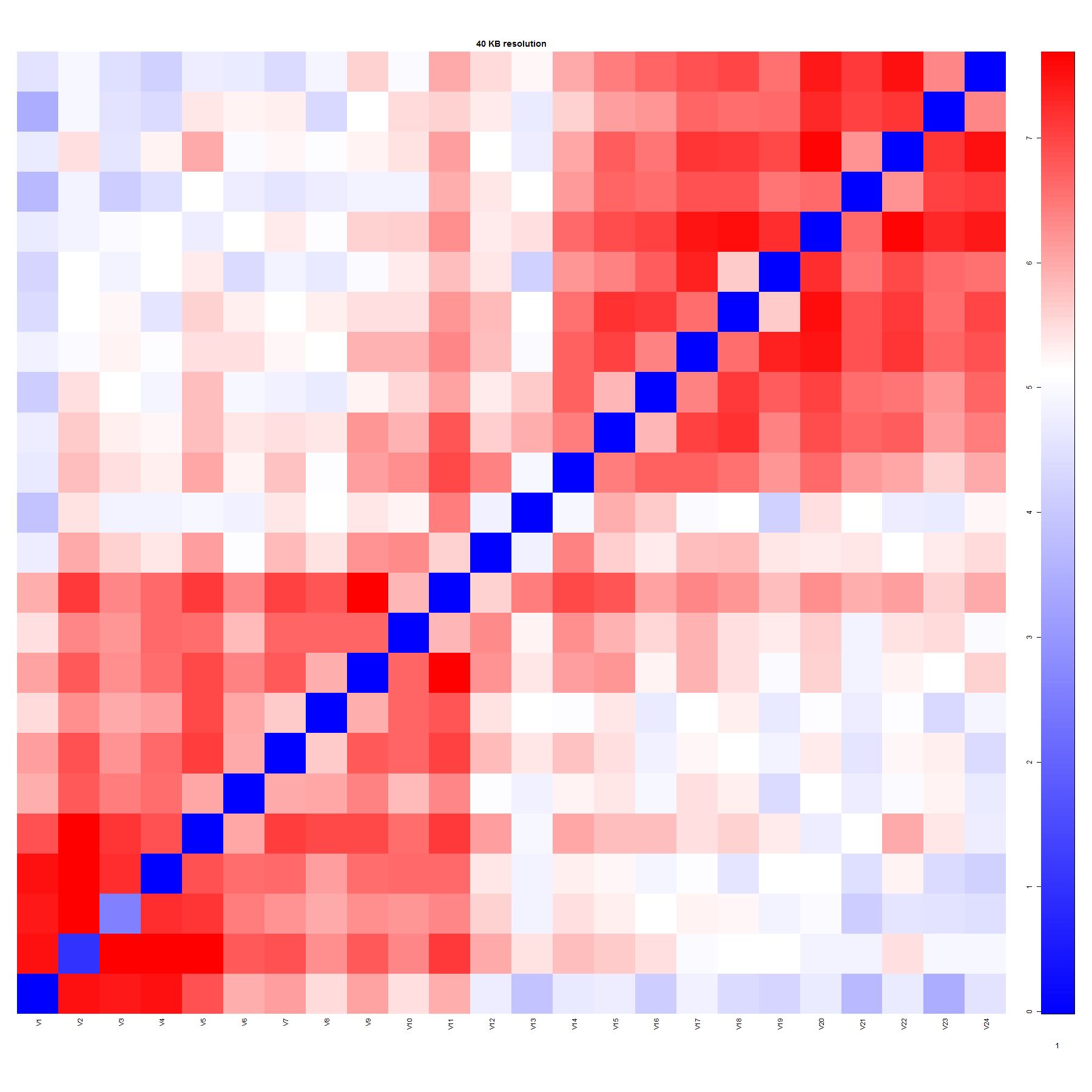 | Domain 4, NcoI  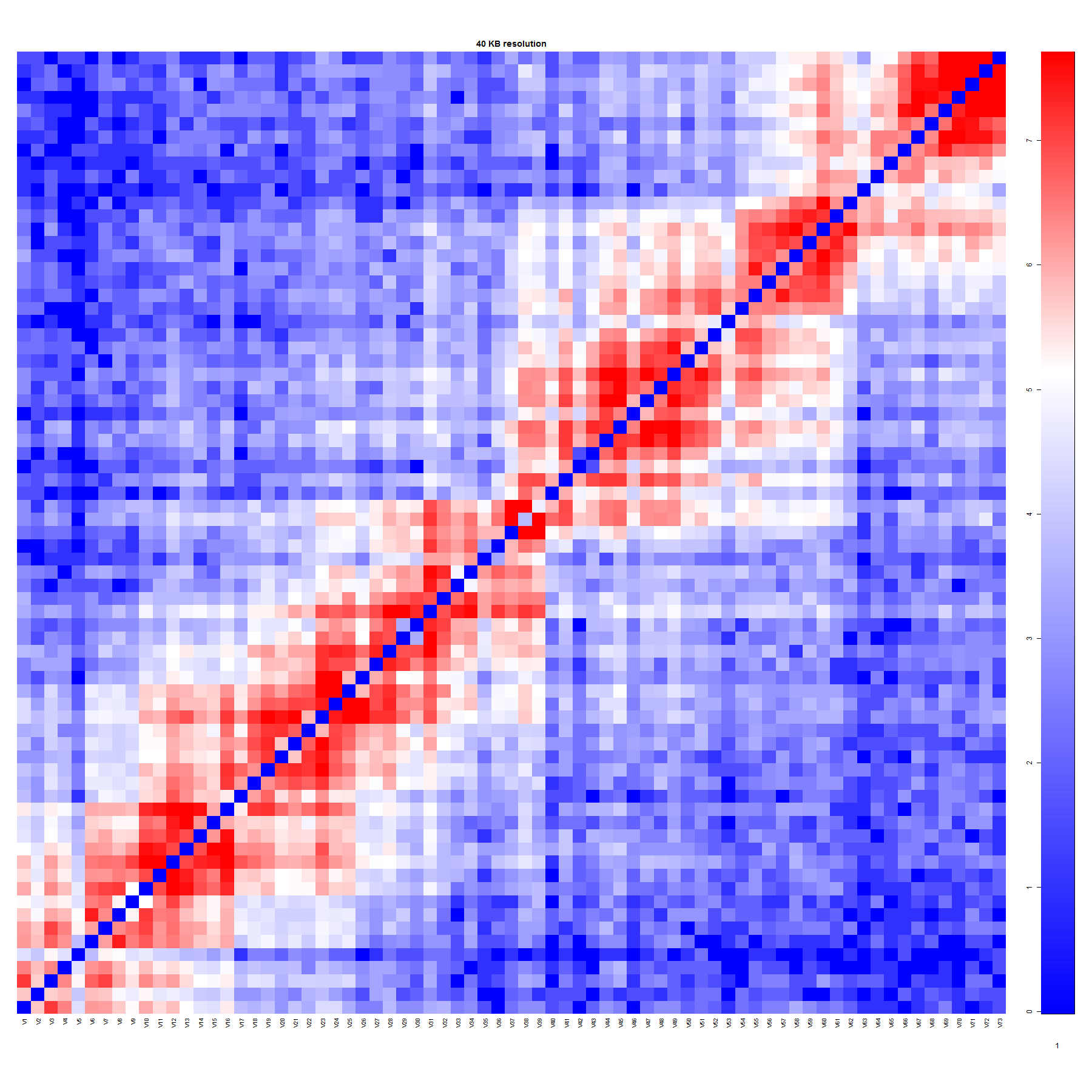 |

**B.**

| Domain 1, HindIII  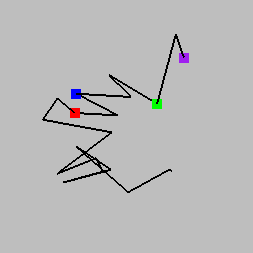 | Domain 2, HindIII  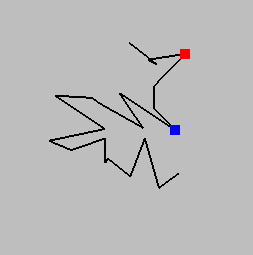 | Domain 3, HindIII  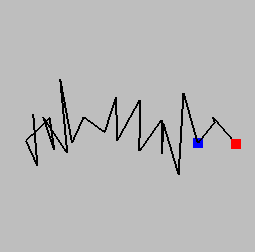 | Domain 4, HindIII  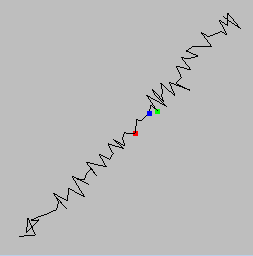 |
| --- | --- | --- | --- |
| Domain 1, NcoI  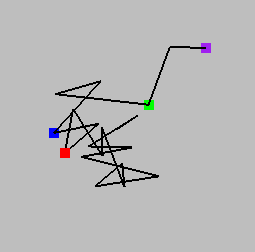 | Domain 2, NcoI  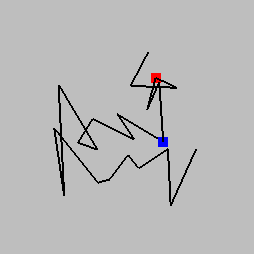 | Domain 3, NcoI  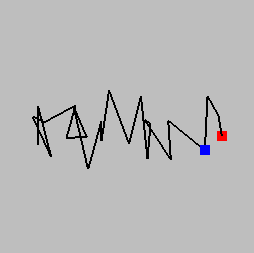 | Domain 4, NcoI  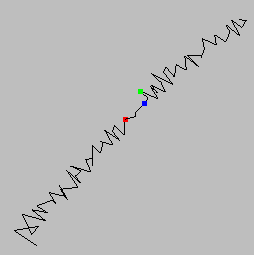 |

**C. D.**

**
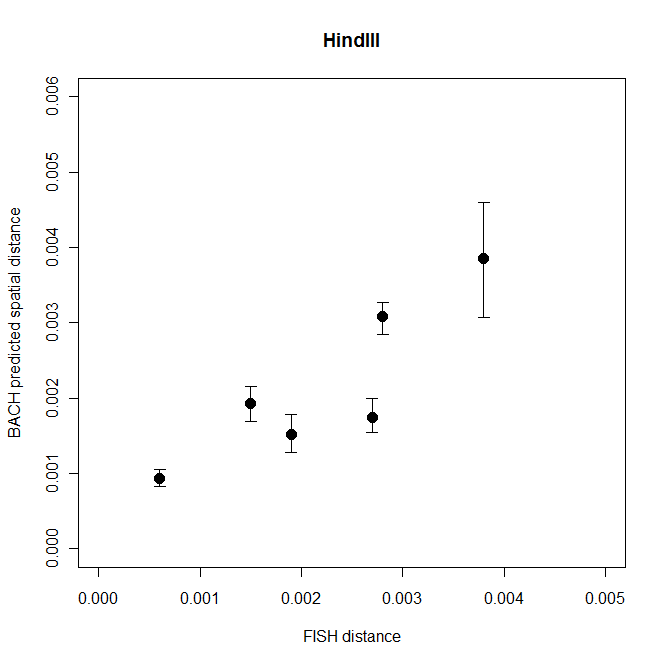

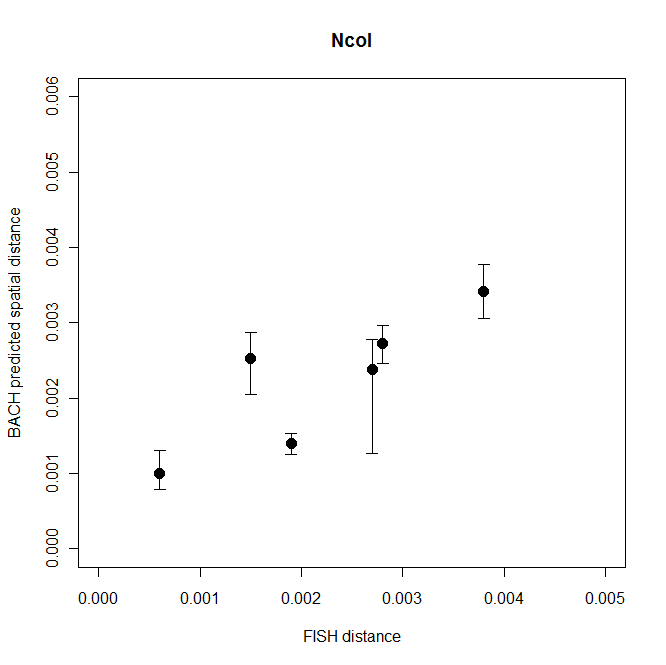
**
