# Supplementary material for: Bayesian Inference of Spatial Organizations of Chromosomes
Source: PLoS Comput Biol. 2013 Jan 31;9(1):e1002893. doi: 10.1371/journal.pcbi.1002893 (PMC3561073; doi:10.1371/journal.pcbi.1002893)
Supplement: Figure S5 — The alignment of two 3D chromosomal structures BACH predicted in the two stages, and , from 20 mouse chromosomes in both HindIII sample and NcoI sample. Red lines represent the first BACH prediction . Blue lines represent the second BACH prediction . (A) The HindIII sample (B) The NcoI sample. (DOCX) [file pcbi.1002893.s005.docx]

**A.**

| Chromosome 1  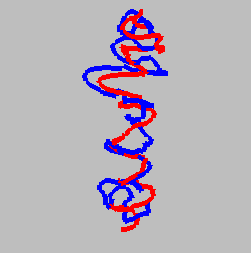 | Chromosome 2  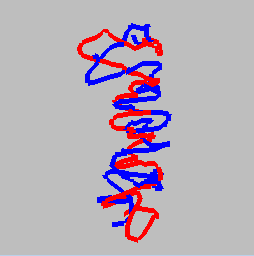 | Chromosome 3  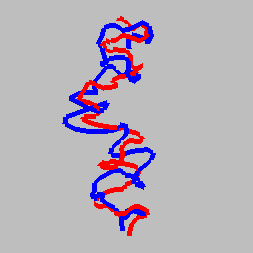 | Chromosome 4  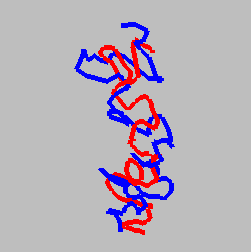 | Chromosome 5  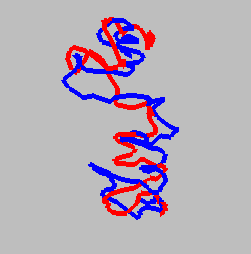 |
| --- | --- | --- | --- | --- |
| Chromosome 6  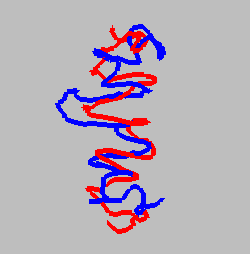 | Chromosome 7  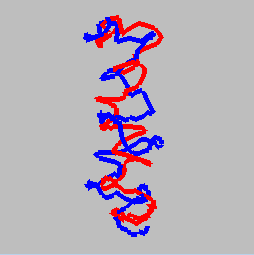 | Chromosome 8  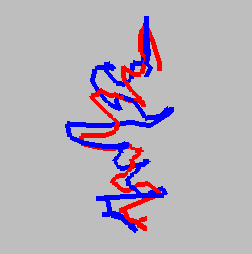 | Chromosome 9  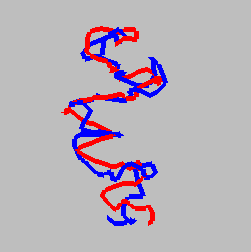 | Chromosome 10  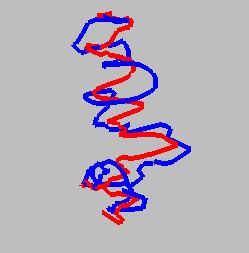 |
| Chromosome 11  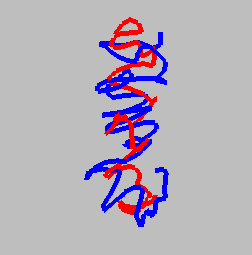 | Chromosome 12  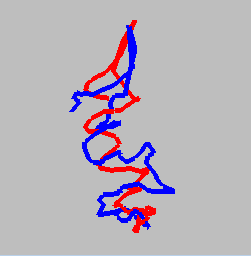 | Chromosome 13  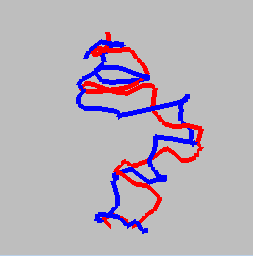 | Chromosome 14  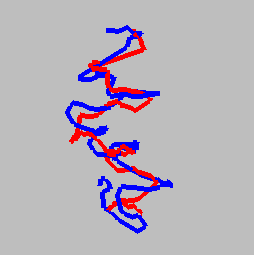 | Chromosome 15  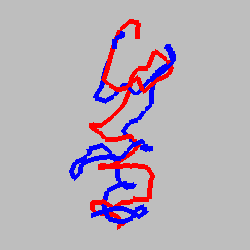 |
| Chromosome 16  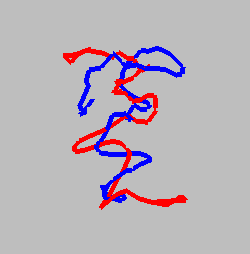 | Chromosome 17  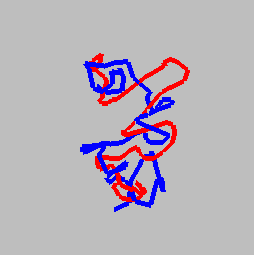 | Chromosome 18  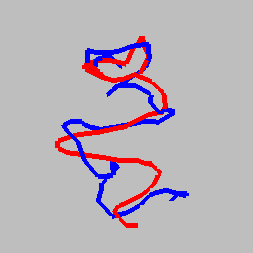 | Chromosome 19  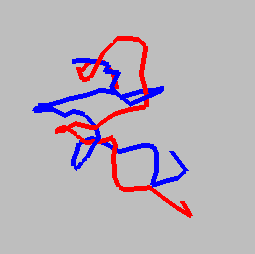 | Chromosome X  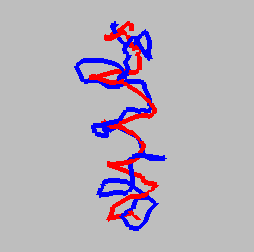 |

**B.**

| Chromosome 1  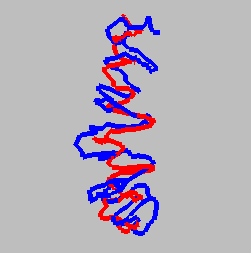 | Chromosome 2  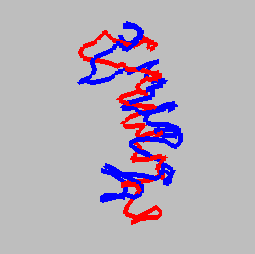 | Chromosome 3  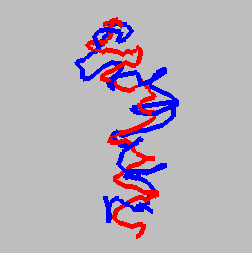 | Chromosome 4  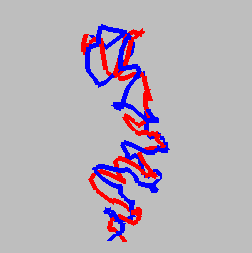 | Chromosome 5  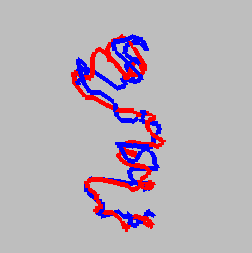 |
| --- | --- | --- | --- | --- |
| Chromosome 6  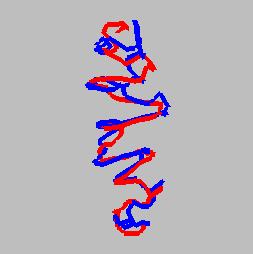 | Chromosome 7  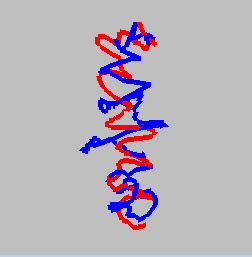 | Chromosome 8  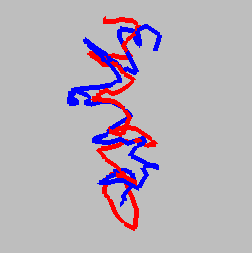 | Chromosome 9  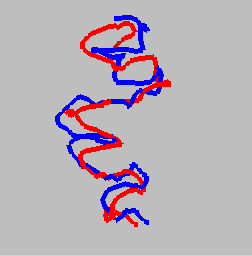 | Chromosome 10  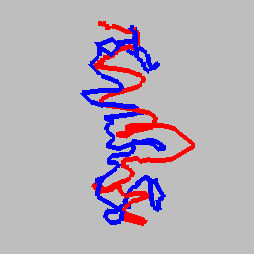 |
| Chromosome 11  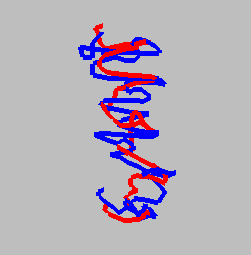 | Chromosome 12  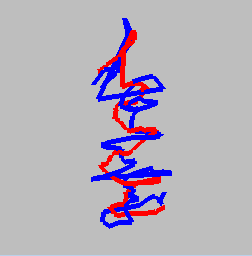 | Chromosome 13  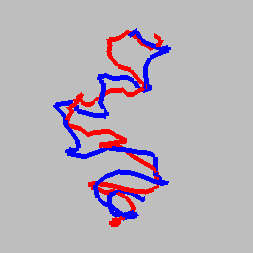 | Chromosome 14  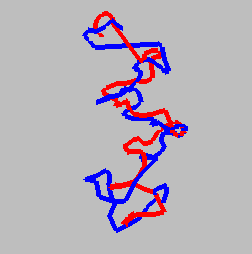 | Chromosome 15  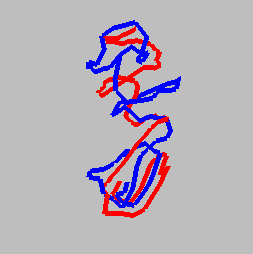 |
| Chromosome 16  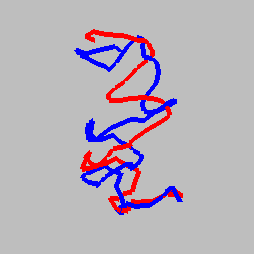 | Chromosome 17  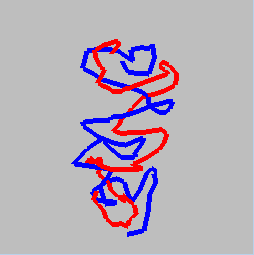 | Chromosome 18  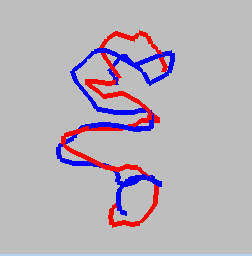 | Chromosome 19  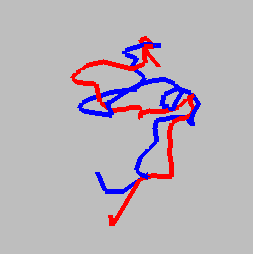 | Chromosome X  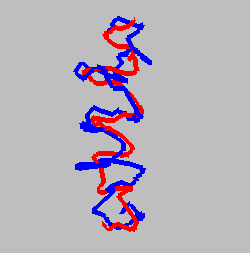 |
