# Supplementary material for: Bayesian Inference of Spatial Organizations of Chromosomes
Source: PLoS Comput Biol. 2013 Jan 31;9(1):e1002893. doi: 10.1371/journal.pcbi.1002893 (PMC3561073; doi:10.1371/journal.pcbi.1002893)
Supplement: Figure S7 — Comparison of reproducibility between BACH, BACH-SUB (a modified BACH algorithm without bias correction) and MCMC5C using the high resolution Hi-C dataset on mouse embryonic stem cells. We focus on long chromosomes (chr 1 to chr 14 and chr X). (A) 3D chromosomal structures predicted by BACH using the mouse Hi-C data. Red lines and blue lines represent the HindIII sample and the NcoI sample, respectively. (B) 3D chromosomal structures predicted by BACH-SUB using the mouse Hi-C data. Red lines and blue lines represent the HindIII sample and the NcoI sample, respectively. (C) 3D chromosomal structures predicted by MCMC5C using the mouse Hi-C data. Red lines and blue lines represent the HindIII sample and the NcoI sample, respectively. (D) The normalized RMSDs of 3D chromosomal structures predicted from the HindIII sample and the NcoI sample, using BACH, BACH-SUB and MCMC5C. BACH achieved significantly higher reproducibility than MCMC5C (paired t-test p-value = 1.4e-7). BACH-SUB also achieved significantly higher reproducibility than MCMC5C (paired t-test p-value = 0.0465). (DOCX) [file pcbi.1002893.s007.docx]

**A.**

| Chromosome 1  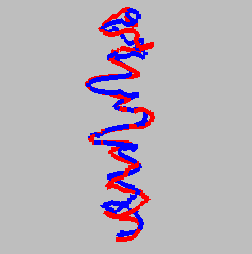 | Chromosome 2  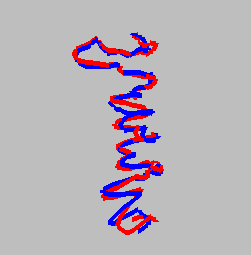 | Chromosome 3  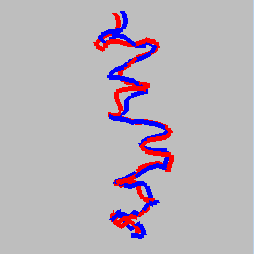 | Chromosome 4  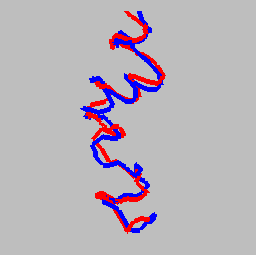 | Chromosome 5  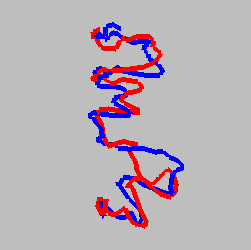 |
| --- | --- | --- | --- | --- |
| Chromosome 6  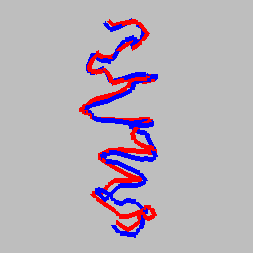 | Chromosome 7  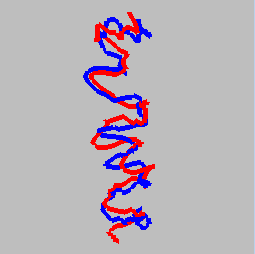 | Chromosome 8  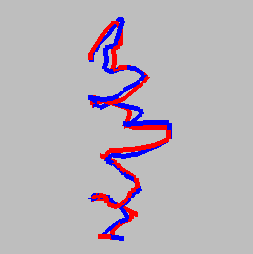 | Chromosome 9  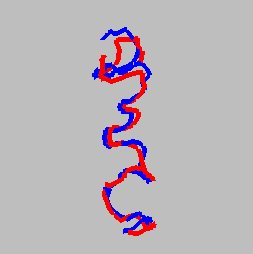 | Chromosome 10  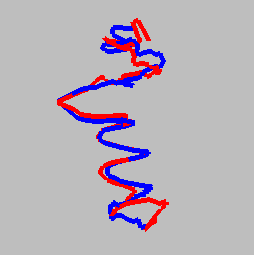 |
| Chromosome 11  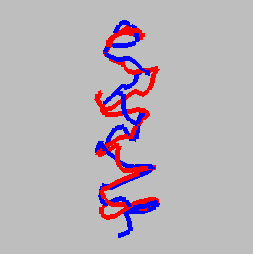 | Chromosome 12  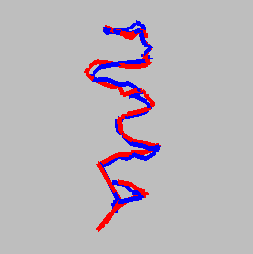 | Chromosome 13  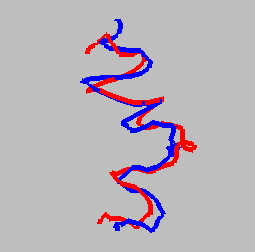 | Chromosome 14  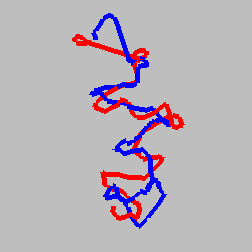 | Chromosome X  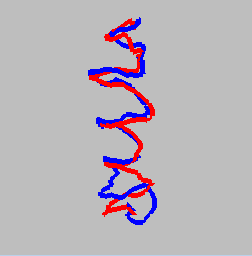 |
|  |  |  |  |  |

**B.**

| Chromosome 1  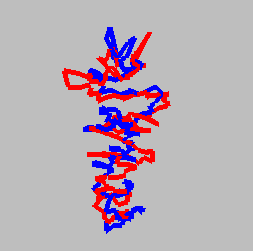 | Chromosome 2  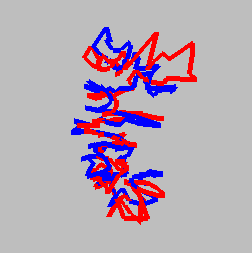 | Chromosome 3  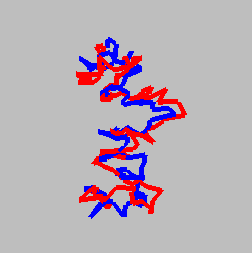 | Chromosome 4  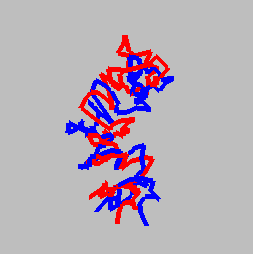 | Chromosome 5  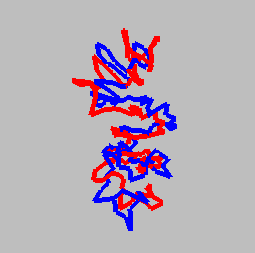 |
| --- | --- | --- | --- | --- |
| Chromosome 6  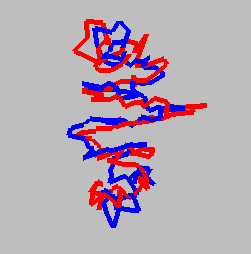 | Chromosome 7  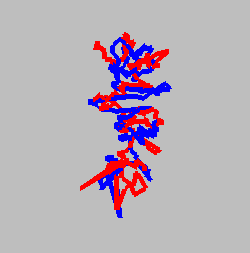 | Chromosome 8  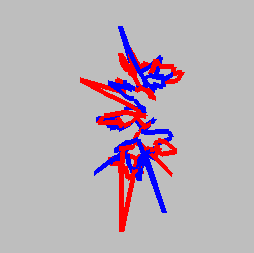 | Chromosome 9  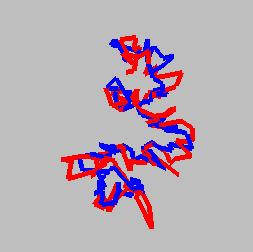 | Chromosome 10  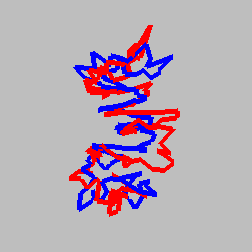 |
| Chromosome 11  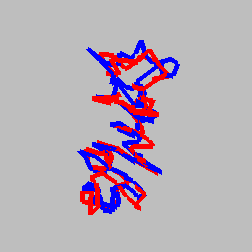 | Chromosome 12  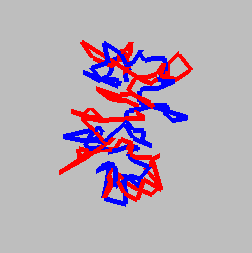 | Chromosome 13  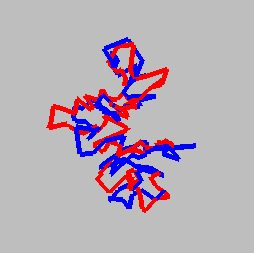 | Chromosome 14  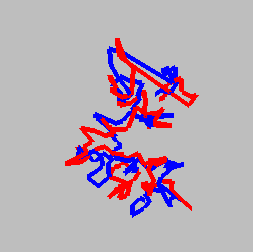 | Chromosome X  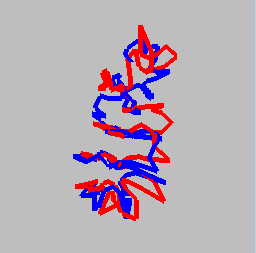 |
|  |  |  |  |  |

**C.**

| Chromosome 1  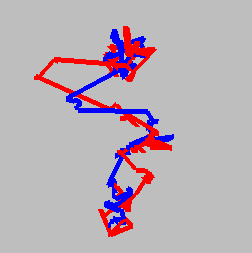 | Chromosome 2  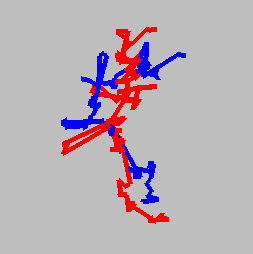 | Chromosome 3  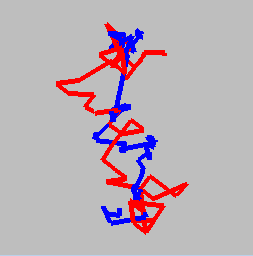 | Chromosome 4  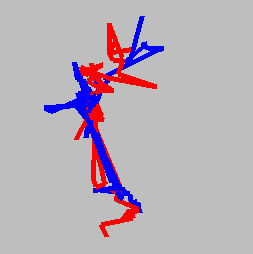 | Chromosome 5  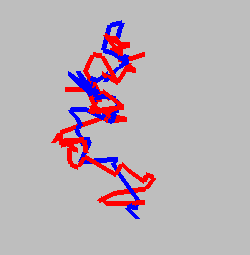 |
| --- | --- | --- | --- | --- |
| Chromosome 6  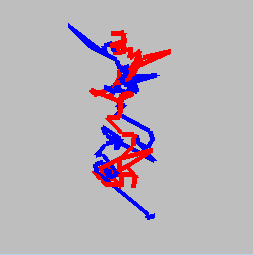 | Chromosome 7  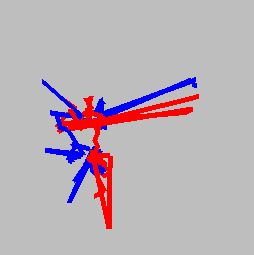 | Chromosome 8  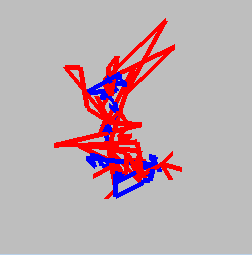 | Chromosome 9  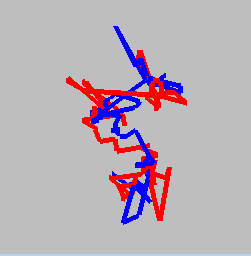 | Chromosome 10  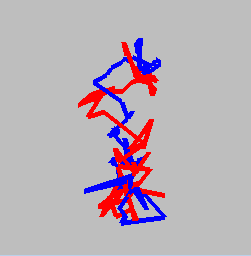 |
| Chromosome 11  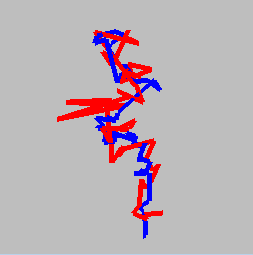 | Chromosome 12  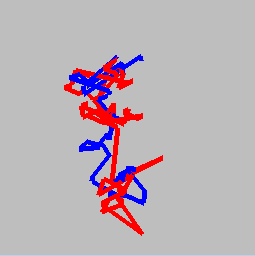 | Chromosome 13  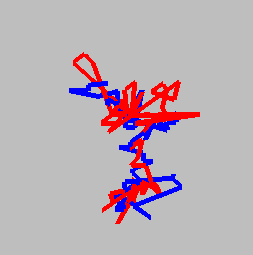 | Chromosome 14  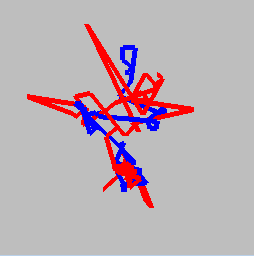 | Chromosome X  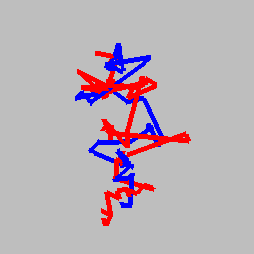 |
|  |  |  |  |  |

**D.**

**
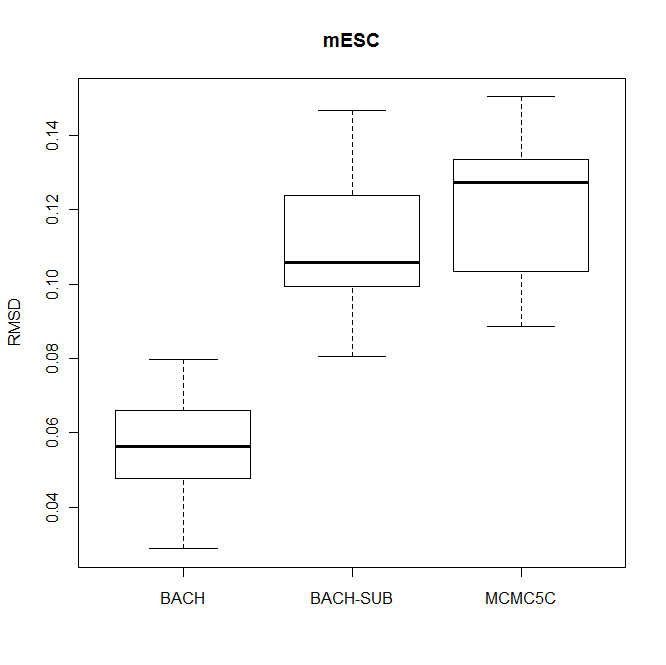
**
