# Supplementary material for: Bayesian Inference of Spatial Organizations of Chromosomes
Source: PLoS Comput Biol. 2013 Jan 31;9(1):e1002893. doi: 10.1371/journal.pcbi.1002893 (PMC3561073; doi:10.1371/journal.pcbi.1002893)
Supplement: Figure S10 — The flow chart of the BACH and BACH-MIX algorithm. (DOCX) [file pcbi.1002893.s010.docx]

BACH input: Hi-C contact matrix, local genomic features: restriction enzyme cutting site, GC content, sequence uniqueness.

BACH update stage 1: use Poisson regression approach to obtain the initial values for the nuisance parameters.

BACH

BACH update stage 2: use sequential importance sampling to obtain the initial 3D chromosomal structure.

BACH-MIX

BACH-MIX output: the posterior distribution of the proportion of the spatial arrangement of two adjacent genomic regions.

BACH-MIX input: the 3D chromosomal structures of two adjacent genomic regions and the nuisance parameters obtained from the BACH output.

BACH-MIX update: use hybrid Monte Carlo to sample the proportion of the spatial arrangement of two adjacent genomic regions.

BACH output: the posterior distributions for the 3D chromosomal structure and the nuisance parameters.

BACH update stage 3: use Gibbs sampler to refine the 3D chromosomal structure and the nuisance parameters.
