# Supplementary material for: Bayesian Inference of Spatial Organizations of Chromosomes
Source: PLoS Comput Biol. 2013 Jan 31;9(1):e1002893. doi: 10.1371/journal.pcbi.1002893 (PMC3561073; doi:10.1371/journal.pcbi.1002893)
Supplement: Table S1 — Pearson correlation coefficients between HD ratios and genomic and epigenetic features. (DOCX) [file pcbi.1002893.s013.docx]

**Table S1. Pearson correlation coefficients between HD ratios and genomic and epigenetic features.**

|  |  |  | |  |  |
| --- | --- | --- | --- | --- | --- |
| Genomic and epigenetic features | The HindIII sample | | | The NcoI sample | |
|  | Cor^*^ | | p-value | Cor^*^ | p-value |
| Gene density | 0.31 | | <2.2e-16 | 0.35 | <2.2e-16 |
| Gene expression | 0.17 | | 2.8e-9 | 0.17 | 1.7e-9 |
| H3K36me3 | 0.33 | | <2.2e-16 | 0.45 | <2.2e-16 |
| H3K27me3 | 0.14 | | 4.9e-7 | 0.23 | 2.2e-15 |
| H3K4me3 | 0.40 | | <2.2e-16 | 0.45 | <2.2e-16 |
| RNA polymerase II | 0.36 | | <2.2e-16 | 0.40 | <2.2e-16 |
| Chromatin accessibility | 0.42 | | <2.2e-16 | 0.54 | <2.2e-16 |
| DNA replication time | 0.38 | | <2.2e-16 | 0.51 | <2.2e-16 |
| H3K9me3 | -0.31 | | <2.2e-16 | -0.44 | <2.2e-16 |
| H4K20me3 | -0.33 | | <2.2e-16 | -0.46 | <2.2e-16 |
| Genome-nuclear lamina interaction | -0.37 | | <2.2e-16 | -0.51 | <2.2e-16 |
|  |  | |  |  |  |

^*^Pearson correlation coefficients between the HD ratio and genomic and epigenetic features.
