# Supplementary material for: Bayesian Inference of Spatial Organizations of Chromosomes
Source: PLoS Comput Biol. 2013 Jan 31;9(1):e1002893. doi: 10.1371/journal.pcbi.1002893 (PMC3561073; doi:10.1371/journal.pcbi.1002893)
Supplement: Table S2 — The value and the rank of genomic and epigenetic features for a more elongated domain (chromosome 18, 33,960,000∼34,960,000, in the HindIII sample) and a less elongated domain (chromosome 7, 62,040,000∼63,040,000, in the HindIII sample). (DOCX) [file pcbi.1002893.s014.docx]

**Table S2. The value and the rank of genomic and epigenetic features for a more elongated domain (chromosome 18, 33,960,000~34,960,000, in the HindIII sample) and a less elongated domain (chromosome 7, 62,040,000~63,040,000, in the HindIII sample).**

| Genomic and epigenetic features | More elongated domain | | Less elongated domain | |
| --- | --- | --- | --- | --- |
|  | Value | Rank* | Value | Rank* |
| Compartment label | A | NA | B | NA |
| Gene density | 16 | 180 | 2 | 923 |
| Gene expression | 4.789 | 89 | 0.003 | 1,049 |
| H3K36me3 | 0.767 | 73 | 0.006 | 1,122 |
| H3K27me3 | 0.151 | 566 | -1.488 | 951 |
| H3K4me3 | 0.044 | 68 | 0.005 | 845 |
| RNA polymerase II | 0.014 | 157 | 0.006 | 643 |
| Chromatin accessibility | 0.106 | 80 | 0.004 | 1,143 |
| DNA replication time | 1.916 | 6 | -0.816 | 961 |
| H3K9me3 | -0.299 | 887 | 0.196 | 172 |
| H4K20me3 | -0.386 | 883 | 0.101 | 299 |
| Genome-nuclear lamina interaction | -0.953 | 1,179 | 0.645 | 147 |
| HD ratio | 2.158 | 146 | 1.140 | 842 |
|  |  |  |  |  |

*The rank in descending order among the selected 1,199 topological domains.
