# Supplementary material for: Bayesian Inference of Spatial Organizations of Chromosomes
Source: PLoS Comput Biol. 2013 Jan 31;9(1):e1002893. doi: 10.1371/journal.pcbi.1002893 (PMC3561073; doi:10.1371/journal.pcbi.1002893)
Supplement: Table S3 — The number of structures with proportion larger than 10% and 1%. (DOCX) [file pcbi.1002893.s015.docx]

**Table S3.** **The number of structures with proportion larger than 10% and 1%.**

|  |  |  |  |  |
| --- | --- | --- | --- | --- |
| Sample | Threshold | Domain center | Domain boundary | P-value |
| HindIII | Proportion > 10% | 1.65 | 2.18 | <2.2e-16 |
|  | Proportion > 1% | 3.77 | 4.22 | 2.1e-7 |
| NcoI | Proportion > 10% | 1.60 | 1.99 | <2.2e-16 |
|  | Proportion > 1% | 3.48 | 3.84 | 7.2e-5 |
|  |  |  |  |  |
