# Supplementary material for: Bayesian Inference of Spatial Organizations of Chromosomes
Source: PLoS Comput Biol. 2013 Jan 31;9(1):e1002893. doi: 10.1371/journal.pcbi.1002893 (PMC3561073; doi:10.1371/journal.pcbi.1002893)
Supplement: Table S4 — The structural variations of chromatin correlate with genetic and epigenetic features. (A) In the HindIII sample, the structural variations correlate with genetic and epigenetic features. (B) In the NcoI sample, the structural variations correlate with genetic and epigenetic features. (DOCX) [file pcbi.1002893.s016.docx]

**Table S4. The structural variations of chromatin correlate with genetic and epigenetic features.**

**(A)** In the HindIII sample, the structural variations correlate with genetic and epigenetic features.

|  |  |  |  |  |
| --- | --- | --- | --- | --- |
| Genomic and epigenetic features | Low^1^ | High^2^ | Diff^3^ | P-value^4^ |
| Gene density | 5.0724 | 7.4052 | 2.3327 | **0.0003** |
| Gene expression | 1.2926 | 1.5806 | 0.2880 | 0.1625 |
| H3K36me3 | -0.6512 | -0.4699 | 0.1813 | **0.0017** |
| H3K27me3 | 0.1328 | 0.1927 | 0.0599 | **0.0500** |
| H3K4me3 | 0.0097 | 0.0140 | 0.0043 | **3.0E-5** |
| RNA polymerase II | 0.0067 | 0.0082 | 0.0016 | **0.0002** |
| Chromatin accessibility | 0.0346 | 0.0462 | 0.0116 | **1.9E-05** |
| DNA replication time | -0.2214 | 0.0392 | 0.2606 | **0.0006** |
| H3K9me3 | -0.0836 | -0.1682 | -0.0846 | **0.0001** |
| H4K20me3 | -0.1531 | -0.2222 | -0.0691 | **0.0199** |
| Genome-nuclear lamina interaction | 0.1098 | -0.0482 | -0.1580 | **0.0011** |
|  |  |  |  |  |

^1^Mean of genetic and epigenetic features in regions with low structural variations.

^2^Mean of genetic and epigenetic features in regions with high structural variations.

^3^Difference of genetic and epigenetic features between regions with high structural variations and regions with low structural variations.

^4^Two sample t-test p-value. Significant p-values (< 0.05) are highlighted in bold font.

**(B)** In the NcoI sample, the structural variations correlate with genetic and epigenetic features.

|  |  |  |  |  |
| --- | --- | --- | --- | --- |
| Genomic and epigenetic features | Low^1^ | High^2^ | Diff^3^ | P-value^4^ |
| Gene density | 5.4071 | 7.4885 | 2.0814 | **0.0011** |
| Gene expression | 1.3047 | 1.6100 | 0.3053 | 0.1418 |
| H3K36me3 | -0.6686 | -0.4350 | 0.2335 | **2.6E-05** |
| H3K27me3 | 0.1196 | 0.2090 | 0.0894 | **0.0010** |
| H3K4me3 | 0.0100 | 0.0144 | 0.0044 | **1.7E-05** |
| RNA polymerase II | 0.0068 | 0.0083 | 0.0015 | **0.0003** |
| Chromatin accessibility | 0.0347 | 0.0476 | 0.0129 | **1.0E-06** |
| DNA replication time | -0.2379 | 0.0837 | 0.3215 | **8.0E-06** |
| H3K9me3 | -0.0926 | -0.1733 | -0.0807 | **4.7E-05** |
| H4K20me3 | -0.1346 | -0.2433 | -0.1086 | **0.0001** |
| Genome-nuclear lamina interaction | 0.1387 | -0.0875 | -0.2262 | **1.0E-06** |
|  |  |  |  |  |

^1^Mean of genetic and epigenetic features in regions with low structural variations.

^2^Mean of genetic and epigenetic features in regions with high structural variations.

^3^Difference of genetic and epigenetic features between regions with high structural variations and regions with low structural variations.

^4^Two sample t-test p-value. Significant p-values (< 0.05) are highlighted in bold font.
