# Supplementary material for: Bayesian Inference of Spatial Organizations of Chromosomes
Source: PLoS Comput Biol. 2013 Jan 31;9(1):e1002893. doi: 10.1371/journal.pcbi.1002893 (PMC3561073; doi:10.1371/journal.pcbi.1002893)
Supplement: Table S5 — The mean of six RMSDs across 100 simulated datasets and the number of RMSDs below 5% quantile with different mixture proportions. The 5% quantile of RMSD is calculated from the empirical distribution of RMSD, which is the empirical distribution of 100 RMSD(A, B). (DOCX) [file pcbi.1002893.s017.docx]

**Table S5. The mean of six RMSDs across 100 simulated datasets and the number of RMSDs below 5% quantile with different mixture proportions.** The 5% quantile of RMSD is calculated from the empirical distribution of RMSD, which is the empirical distribution of 100 RMSD(A, B).

|  |  |  |  |  |  |  |
| --- | --- | --- | --- | --- | --- | --- |
|  |  | A 50% | A 60% | A 70% | A 80% | A 90% |
|  |  | B 50% | B 40% | B 30% | B 20% | B 10% |
|  | A_B | 0.1584 | 0.1594 | 0.1566 | 0.1656 | 0.1589 |
|  | S1_A | 0.1136 | 0.1050 | 0.0876 | 0.0707 | 0.0475 |
| Mean | S1_B | 0.1113 | 0.1239 | 0.1266 | 0.1474 | 0.1512 |
| RMSD | S2_A | 0.1201 | 0.1190 | 0.1011 | 0.0877 | 0.0572 |
|  | S2_B | 0.1225 | 0.1273 | 0.1332 | 0.1511 | 0.1514 |
|  | S1_S2 | 0.0985 | 0.0988 | 0.0889 | 0.0822 | 0.0625 |
|  | S1_A | 55 | 58 | 74 | 87 | 96 |
| # of RMSDs | S1_B | 57 | 31 | 28 | 13 | 11 |
| < 5% quantile | S2_A | 40 | 36 | 61 | 77 | 94 |
|  | S2_B | 42 | 22 | 18 | 9 | 6 |
|  | S1_S2 | 74 | 66 | 81 | 84 | 91 |
|  |  |  |  |  |  |  |
