# Supplementary material for: Bayesian Inference of Spatial Organizations of Chromosomes
Source: PLoS Comput Biol. 2013 Jan 31;9(1):e1002893. doi: 10.1371/journal.pcbi.1002893 (PMC3561073; doi:10.1371/journal.pcbi.1002893)
Supplement: Table S6 — Applying the two-step procedure to the real Hi-C data, treat each topological domain as an individual unit. The RMSD between two 3D chromosomal structures BACH predicted in the two stages, and , from 20 mouse chromosomes in both HindIII sample and NcoI sample. The tail probabilities less than 0.05 are highlighted in bold font. (DOCX) [file pcbi.1002893.s018.docx]

**Table S6. Applying the two-step procedure to the real Hi-C data, treat each topological domain as an individual unit.** The RMSD between two 3D chromosomal structures BACH predicted in the two stages, $S_{1}$ and $S_{2}$, from 20 mouse chromosomes in both HindIII sample and NcoI sample. The tail probabilities less than 0.05 are highlighted in bold font.

|  |  |  |  |  |
| --- | --- | --- | --- | --- |
| Chromosome | The HindIII sample | | The NcoI sample | |
|  | RMSD | Tail probability | RMSD | Tail probability |
| 1 | 0.0812 | **0.000** | 0.0795 | **0.000** |
| 2 | 0.0762 | **0.000** | 0.0891 | **0.002** |
| 3 | 0.0766 | **0.000** | 0.0922 | **0.004** |
| 4 | 0.0941 | **0.010** | 0.0866 | **0.000** |
| 5 | 0.0961 | **0.015** | 0.0772 | **0.003** |
| 6 | 0.0883 | **0.006** | 0.0785 | **0.000** |
| 7 | 0.0936 | **0.011** | 0.1080 | **0.050** |
| 8 | 0.1078 | **0.032** | 0.1062 | **0.030** |
| 9 | 0.0814 | **0.001** | 0.0707 | **0.000** |
| 10 | 0.0953 | **0.010** | 0.1038 | **0.022** |
| 11 | 0.0999 | **0.021** | 0.0886 | **0.005** |
| 12 | 0.1086 | **0.040** | 0.1015 | **0.023** |
| 13 | 0.1075 | **0.028** | 0.1032 | **0.020** |
| 14 | 0.0944 | **0.006** | 0.0974 | **0.007** |
| 15 | 0.1206 | 0.098 | 0.1253 | 0.129 |
| 16 | 0.1397 | 0.232 | 0.1375 | 0.216 |
| 17 | 0.1520 | 0.362 | 0.1454 | 0.285 |
| 18 | 0.1435 | 0.314 | 0.1223 | 0.120 |
| 19 | 0.1523 | 0.358 | 0.1376 | 0.199 |
| X | 0.0906 | **0.009** | 0.0839 | **0.002** |
|  |  |  |  |  |
