# Supplementary material for: Bayesian Inference of Spatial Organizations of Chromosomes
Source: PLoS Comput Biol. 2013 Jan 31;9(1):e1002893. doi: 10.1371/journal.pcbi.1002893 (PMC3561073; doi:10.1371/journal.pcbi.1002893)
Supplement: Table S7 — Fisher's exact test to quantify the magnitude of spatial separations of genomic and epigenetic features. We focus on long chromosomes (chr 1 to chr 14 and chr X). Each number represents the number of chromosome with significant spatial separation pattern. The p-value threshold is 0.05. (DOCX) [file pcbi.1002893.s019.docx]

**Table S7. Fisher’s exact test to quantify the magnitude of spatial separations of genomic and epigenetic features.** We focus on long chromosomes (chr 1 to chr 14 and chr X). Each number represents the number of chromosome with significant spatial separation pattern. The p-value threshold is 0.05.

|  |  |  |
| --- | --- | --- |
| Genomic and epigenetic features | The HindIII sample | The NcoI sample |
| Compartment label | 14 | 14 |
| Gene density | 12 | 13 |
| Gene expression | 6 | 6 |
| H3K36me3 | 12 | 11 |
| H3K27me3 | 10 | 10 |
| H3K4me3 | 14 | 14 |
| RNA polymerase II | 9 | 10 |
| Chromatin accessibility | 15 | 13 |
| DNA replication time | 14 | 12 |
| H3K9me3 | 12 | 12 |
| H4K20me3 | 13 | 13 |
| Genome-nuclear lamina interaction | 15 | 13 |
|  |  |  |
