# Supplementary material for: Bayesian Inference of Spatial Organizations of Chromosomes
Source: PLoS Comput Biol. 2013 Jan 31;9(1):e1002893. doi: 10.1371/journal.pcbi.1002893 (PMC3561073; doi:10.1371/journal.pcbi.1002893)
Supplement: Table S8 — Eleven FISH probes used in a study of the mESC (supplementary reference [10] in Text S1 ). (DOCX) [file pcbi.1002893.s020.docx]

**Table S8. Eleven FISH probes used in a study of the mESC (supplementary reference [10]).**

|  |  |  |  |  |
| --- | --- | --- | --- | --- |
| Name | ID | Chr | Start | End |
| GCR | G135P63331H1 | Chr 2 | 74242329 | 74282329 |
| Lnp | G13561870C5 | Chr 2 | 74331284 | 74371284 |
| Evx2 | G135P67444A12 | Chr 2 | 74473580 | 74513580 |
| Hoxd3 | G135P67844B8 | Chr 2 | 74566210 | 74606210 |
| Rcn1 | G135P601417F11 | Chr 2 | 105220659 | 105260659 |
| 1550J22 | G135P601672D2 | Chr 2 | 105386498 | 105426498 |
| Il9r | G135P60495H4 | Chr 11 | 32058671 | 32098671 |
| Hbq1 | G135P603718B2 | Chr 11 | 32198791 | 32238791 |
| Calcoco2 | G135P605135H4 | Chr 11 | 95935571 | 95975571 |
| Hoxb9 | G135P65905G4 | Chr 11 | 96115220 | 96155220 |
| Hoxb1 | G135P60460C4 | Chr 11 | 96202060 | 96242060 |
|  |  |  |  |  |
