# Supplementary material for: Bayesian Inference of Spatial Organizations of Chromosomes
Source: PLoS Comput Biol. 2013 Jan 31;9(1):e1002893. doi: 10.1371/journal.pcbi.1002893 (PMC3561073; doi:10.1371/journal.pcbi.1002893)
Supplement: Table S9 — The normalized FISH distances between six probe pairs. (DOCX) [file pcbi.1002893.s021.docx]

**Table S9. The normalized FISH distances between six probe pairs.**

|  |  |
| --- | --- |
| Probe pairs | Normalized FISH distances^1^ |
| Lnp-GCR | 0.0027 |
| Hoxd3-Evx2 | 0.0015 |
| Rcn1-1550J22 | 0.0038 |
| Hbq-Il9r | 0.0019 |
| Hoxb1-Calcoco2 | 0.0028 |
| Hoxb1-Hoxb9 | 0.0006 |

^1^The normalized FISH distances are defined as the spatial distances obtained from the FISH experiment normalized by the radius of each cell.
