# Supplementary material for: Bayesian Inference of Spatial Organizations of Chromosomes
Source: PLoS Comput Biol. 2013 Jan 31;9(1):e1002893. doi: 10.1371/journal.pcbi.1002893 (PMC3561073; doi:10.1371/journal.pcbi.1002893)
Supplement: Table S10 — The annotations of four topological domains containing eleven FISH probes. (DOCX) [file pcbi.1002893.s022.docx]

**Table S10.** **The annotations of four topological domains containing eleven FISH probes.**

|  |  |  |  |  |  |
| --- | --- | --- | --- | --- | --- |
| Domain ID | Chr | Start | End | Size | Genes within domain |
| 1 | 2 | 73762329 | 74606210 | 0.84 MB | GCR, Lnp, Evx2, Hoxd3 |
| 2 | 2 | 105100659 | 106026498 | 0.93 MB | Rcn1, 1550J22 |
| 3 | 11 | 32058671 | 33038791 | 0.98 MB | Il9r, Hbq1 |
| 4 | 11 | 94495571 | 97482060 | 2.99 MB | Calcoco2, Hoxb1, Hoxb9 |
|  |  |  |  |  |  |
