# Supplementary material for: Bayesian Inference of Spatial Organizations of Chromosomes
Source: PLoS Comput Biol. 2013 Jan 31;9(1):e1002893. doi: 10.1371/journal.pcbi.1002893 (PMC3561073; doi:10.1371/journal.pcbi.1002893)
Supplement: Table S11 — Posterior mean and 95% credible interval for parameters in the simulation study with single consensus 3D chromosomal structure. We use the posterior samples in chain 3 (after burn-in and thin) for statistical inference. The true values for , , , and are , , , and , respectively. (DOCX) [file pcbi.1002893.s023.docx]

**Table S11. Posterior mean and 95% credible interval for parameters in the simulation study with single consensus 3D chromosomal structure.** We use the posterior samples in chain 3 (after burn-in and thin) for statistical inference. The true values for $\beta_{0}$, $\beta_{1}$, $\beta_{enz}$, $\beta_{gcc}$ and $\beta_{map}$ are $4$, $-1$, $0.1$, $-0.1$ and $0.1$, respectively.

|  |  |  |  |
| --- | --- | --- | --- |
| Variable | Posterior mean | 2.5% quantile | 97.5% quantile |
| $\beta_{0}$ | 4.0045 | 3.8814 | 4.1186 |
| $\beta_{1}$ | -0.9999 | -1.0206 | -0.9824 |
| $\beta_{enz}$ | 0.1020 | 0.0738 | 0.1278 |
| $\beta_{gcc}$ | -0.0854 | -0.1063 | -0.0644 |
| $\beta_{map}$ | 0.0950 | 0.0721 | 0.1097 |
|  |  |  |  |
