# Supplementary material for: Bayesian Inference of Spatial Organizations of Chromosomes
Source: PLoS Comput Biol. 2013 Jan 31;9(1):e1002893. doi: 10.1371/journal.pcbi.1002893 (PMC3561073; doi:10.1371/journal.pcbi.1002893)
Supplement: Table S12 — The true value, posterior mean and 95% credible interval for the 12 dimensional multinomial distribution used in the simulation study with multiple distinct 3D chromosomal structures. (DOCX) [file pcbi.1002893.s024.docx]

**Table S12. The true value, posterior mean and 95% credible interval for the 12 dimensional multinomial distribution** $\boldsymbol{p}_{\boldsymbol{\Theta}}$ **used in the simulation study with multiple distinct 3D chromosomal structures.**

|  |  |  |  |  |  |  |  |  |
| --- | --- | --- | --- | --- | --- | --- | --- | --- |
| Structure | $\phi$ | $\theta$ | $\psi$ | $I$ | $p_{\Theta}$^1^ | Mean^2^ | 2.50% quantile | 97.50% quantile |
| 1 | 0 | $-\pi/2$ | 0 | 0 | 0.5 | 0.48 | 0.43 | 0.52 |
| 2 | 0 | $-\pi/2$ | $\pi$ | 0 | 0 | 0.00 | 0.00 | 0.01 |
| 3 | 0 | 0 | 0 | 0 | 0 | 0.02 | 0.00 | 0.07 |
| 4 | 0 | 0 | $\pi$ | 0 | 0 | 0.00 | 0.00 | 0.00 |
| 5 | $\pi$ | 0 | 0 | 0 | 0 | 0.01 | 0.00 | 0.03 |
| 6 | $\pi$ | 0 | $\pi$ | 0 | 0 | 0.00 | 0.00 | 0.00 |
| 7 | 0 | $-\pi/2$ | 0 | 1 | 0 | 0.00 | 0.00 | 0.00 |
| 8 | 0 | $-\pi/2$ | $\pi$ | 1 | 0.5 | 0.49 | 0.43 | 0.53 |
| 9 | 0 | 0 | 0 | 1 | 0 | 0.00 | 0.00 | 0.01 |
| 10 | 0 | 0 | $\pi$ | 1 | 0 | 0.00 | 0.00 | 0.02 |
| 11 | $\pi$ | 0 | 0 | 1 | 0 | 0.00 | 0.00 | 0.01 |
| 12 | $\pi$ | 0 | $\pi$ | 1 | 0 | 0.00 | 0.00 | 0.01 |
|  |  |  |  |  |  |  |  |  |

^1^The true value for the 12 dimensional multinomial distribution $p_{\Theta}$.

^2^The posterior mean provided by the BACH-MIX algorithm.
