# Supplementary material for: Bayesian Inference of Spatial Organizations of Chromosomes
Source: PLoS Comput Biol. 2013 Jan 31;9(1):e1002893. doi: 10.1371/journal.pcbi.1002893 (PMC3561073; doi:10.1371/journal.pcbi.1002893)
Supplement: Table S13 — Applying the two-step procedure to the zoomed-in real Hi-C data (equally split one topological domain into two sub-domains), treat each sub-domain as an individual unit. The RMSD between two 3D chromosomal structures BACH predicted in the two stages, and , from 20 mouse chromosomes in both HindIII sample and NcoI sample. The tail probabilities < = 0.05 are highlighted in bold font. (DOCX) [file pcbi.1002893.s025.docx]

**Table S13. Applying the two-step procedure to the zoomed-in real Hi-C data (equally split one topological domain into two sub-domains), treat each sub-domain as an individual unit.** The RMSD between two 3D chromosomal structures BACH predicted in the two stages, $S_{1}$ and $S_{2}$, from 20 mouse chromosomes in both HindIII sample and NcoI sample. The tail probabilities <= 0.05 are highlighted in bold font.

|  |  |  |  |  |
| --- | --- | --- | --- | --- |
| Chromosome | The HindIII sample | | The NcoI sample | |
|  | RMSD | Tail probability | RMSD | Tail probability |
| 1 | 0.0718 | **0.000** | 0.0720 | **0.000** |
| 2 | 0.0630 | **0.000** | 0.0520 | **0.000** |
| 3 | 0.0859 | **0.001** | 0.0759 | **0.000** |
| 4 | 0.0640 | **0.000** | 0.0559 | **0.000** |
| 5 | 0.0751 | **0.003** | 0.0937 | **0.010** |
| 6 | 0.0941 | **0.013** | 0.0744 | **0.000** |
| 7 | 0.0855 | **0.003** | 0.0986 | **0.016** |
| 8 | 0.1073 | **0.032** | 0.0934 | **0.007** |
| 9 | 0.0840 | **0.001** | 0.0883 | **0.002** |
| 10 | 0.1006 | **0.017** | 0.0935 | **0.010** |
| 11 | 0.0998 | **0.021** | 0.0826 | **0.000** |
| 12 | 0.1079 | **0.038** | 0.0879 | **0.006** |
| 13 | 0.1005 | **0.017** | 0.1023 | **0.019** |
| 14 | 0.1340 | 0.184 | 0.1117 | **0.043** |
| 15 | 0.1067 | **0.035** | 0.1091 | **0.040** |
| 16 | 0.1088 | **0.029** | 0.1221 | 0.099 |
| 17 | 0.1230 | 0.102 | 0.1280 | 0.134 |
| 18 | 0.1417 | 0.299 | 0.1416 | 0.299 |
| 19 | 0.1482 | 0.298 | 0.1646 | 0.522 |
| X | 0.0915 | **0.011** | 0.0867 | **0.003** |
|  |  |  |  |  |
