# Supplementary material for: Bayesian Inference of Spatial Organizations of Chromosomes
Source: PLoS Comput Biol. 2013 Jan 31;9(1):e1002893. doi: 10.1371/journal.pcbi.1002893 (PMC3561073; doi:10.1371/journal.pcbi.1002893)
Supplement: Table S14 — Applying the two-step procedure to the zoomed-out real Hi-C data (combine two adjacent topological domains into one super-domain), treat each super-domain as an individual unit. The RMSD between two 3D chromosomal structures BACH predicted in the two stages, and , from 20 mouse chromosomes in both HindIII sample and NcoI sample. The tail probabilities < = 0.05 are highlighted in bold font. (DOCX) [file pcbi.1002893.s026.docx]

**Table S14. Applying the two-step procedure to the zoomed-out real Hi-C data (combine two adjacent topological domains into one super-domain), treat each super-domain as an individual unit.** The RMSD between two 3D chromosomal structures BACH predicted in the two stages, $S_{1}$ and $S_{2}$, from 20 mouse chromosomes in both HindIII sample and NcoI sample. The tail probabilities <= 0.05 are highlighted in bold font.

|  |  |  |  |  |
| --- | --- | --- | --- | --- |
| Chromosome | The HindIII sample | | The NcoI sample | |
|  | RMSD | Tail probability | RMSD | Tail probability |
| 1 | 0.0884 | **0.002** | 0.0675 | **0.000** |
| 2 | 0.0780 | **0.000** | 0.0891 | **0.002** |
| 3 | 0.0866 | **0.002** | 0.0964 | **0.007** |
| 4 | 0.0739 | **0.000** | 0.0700 | **0.000** |
| 5 | 0.0831 | **0.004** | 0.0777 | **0.003** |
| 6 | 0.0969 | **0.017** | 0.0829 | **0.004** |
| 7 | 0.0905 | **0.007** | 0.1103 | 0.061 |
| 8 | 0.1042 | **0.023** | 0.0909 | **0.005** |
| 9 | 0.0923 | **0.004** | 0.0840 | **0.001** |
| 10 | 0.1264 | 0.118 | 0.1244 | 0.099 |
| 11 | 0.0904 | **0.006** | 0.1027 | **0.027** |
| 12 | 0.1215 | 0.105 | 0.1291 | 0.165 |
| 13 | 0.1087 | **0.030** | 0.0942 | **0.012** |
| 14 | 0.1006 | **0.014** | 0.1357 | 0.199 |
| 15 | 0.0991 | **0.023** | 0.0995 | **0.023** |
| 16 | 0.1574 | 0.444 | 0.1503 | 0.348 |
| 17 | 0.1454 | 0.284 | 0.1378 | 0.213 |
| 18 | 0.0928 | **0.005** | 0.0859 | **0.002** |
| 19 | 0.1197 | 0.080 | 0.1141 | 0.055 |
| X | 0.0834 | **0.002** | 0.0779 | **0.001** |
|  |  |  |  |  |
