# Supplementary material for: Bayesian Inference of Spatial Organizations of Chromosomes
Source: PLoS Comput Biol. 2013 Jan 31;9(1):e1002893. doi: 10.1371/journal.pcbi.1002893 (PMC3561073; doi:10.1371/journal.pcbi.1002893)
Supplement: Table S15 — The RMSD between the 3D chromosomal structure inferred from the zoomed-in Hi-C contact matrices and the 3D chromosomal structure inferred from the original Hi-C contact matrices. The tail probabilities < = 0.05 are highlighted in bold font. (DOCX) [file pcbi.1002893.s027.docx]

**Table S15. The RMSD between the 3D chromosomal structure inferred from the zoomed-in Hi-C contact matrices and the 3D chromosomal structure inferred from the original Hi-C contact matrices.** The tail probabilities <= 0.05 are highlighted in bold font.

|  |  |  |  |  |
| --- | --- | --- | --- | --- |
|  | The HindIII sample | | The NcoI sample | |
| Chromosome | RMSD | Tail probability | RMSD | Tail probability |
| 1 | 0.0586 | **0.000** | 0.0580 | **0.000** |
| 2 | 0.0690 | **0.000** | 0.0626 | **0.000** |
| 3 | 0.0656 | **0.000** | 0.0657 | **0.000** |
| 4 | 0.0721 | **0.000** | 0.0640 | **0.000** |
| 5 | 0.0772 | **0.003** | 0.0885 | **0.006** |
| 6 | 0.0758 | **0.000** | 0.0648 | **0.000** |
| 7 | 0.0761 | **0.001** | 0.0702 | **0.001** |
| 8 | 0.0627 | **0.000** | 0.0745 | **0.000** |
| 9 | 0.0920 | **0.004** | 0.0811 | **0.001** |
| 10 | 0.0695 | **0.000** | 0.0806 | **0.002** |
| 11 | 0.0735 | **0.000** | 0.0842 | **0.002** |
| 12 | 0.0856 | **0.005** | 0.0895 | **0.007** |
| 13 | 0.1087 | **0.031** | 0.1014 | **0.017** |
| 14 | 0.1084 | **0.032** | 0.0977 | **0.007** |
| 15 | 0.0958 | **0.018** | 0.1035 | **0.026** |
| 16 | 0.1001 | **0.012** | 0.1091 | **0.030** |
| 17 | 0.1257 | 0.118 | 0.1142 | 0.055 |
| 18 | 0.0872 | **0.004** | 0.1076 | **0.044** |
| 19 | 0.0811 | **0.001** | 0.1140 | 0.055 |
| X | 0.0689 | **0.000** | 0.0598 | **0.000** |
|  |  |  |  |  |
