# Supplementary material for: Bayesian Inference of Spatial Organizations of Chromosomes
Source: PLoS Comput Biol. 2013 Jan 31;9(1):e1002893. doi: 10.1371/journal.pcbi.1002893 (PMC3561073; doi:10.1371/journal.pcbi.1002893)
Supplement: Table S16 — The RMSD between the 3D chromosomal structures inferred from the zoomed-out Hi-C contact matrices and the 3D chromosomal structures inferred from the original Hi-C contact matrices. The tail probabilities < = 0.05 are highlighted in bold font. (DOCX) [file pcbi.1002893.s028.docx]

**Table S16. The RMSD between the 3D chromosomal structures inferred from the zoomed-out Hi-C contact matrices and the 3D chromosomal structures inferred from the original Hi-C contact matrices.** The tail probabilities <= 0.05 are highlighted in bold font.

|  |  |  |  |  |
| --- | --- | --- | --- | --- |
|  | The HindIII sample | | The NcoI sample | |
| Chromosome | RMSD | Tail probability | RMSD | Tail probability |
| 1 | 0.0648 | **0.000** | 0.0648 | **0.000** |
| 2 | 0.0561 | **0.000** | 0.0473 | **0.000** |
| 3 | 0.0279 | **0.000** | 0.0386 | **0.000** |
| 4 | 0.0749 | **0.000** | 0.0626 | **0.000** |
| 5 | 0.0598 | **0.000** | 0.0554 | **0.000** |
| 6 | 0.0379 | **0.000** | 0.0449 | **0.000** |
| 7 | 0.0623 | **0.000** | 0.0664 | **0.000** |
| 8 | 0.0884 | **0.001** | 0.0832 | **0.000** |
| 9 | 0.0698 | **0.000** | 0.0567 | **0.000** |
| 10 | 0.0743 | **0.001** | 0.0884 | **0.005** |
| 11 | 0.0617 | **0.000** | 0.0511 | **0.000** |
| 12 | 0.0776 | **0.000** | 0.0740 | **0.000** |
| 13 | 0.0599 | **0.000** | 0.0805 | **0.002** |
| 14 | 0.0495 | **0.000** | 0.0972 | **0.007** |
| 15 | 0.0786 | **0.001** | 0.0648 | **0.000** |
| 16 | 0.0277 | **0.000** | 0.0870 | **0.004** |
| 17 | 0.0904 | **0.007** | 0.0745 | **0.001** |
| 18 | 0.0246 | **0.000** | 0.0529 | **0.000** |
| 19 | 0.0768 | **0.000** | 0.0914 | **0.007** |
| X | 0.0761 | **0.001** | 0.0704 | **0.001** |
|  |  |  |  |  |
