# Supplementary material for: Bayesian Inference of Spatial Organizations of Chromosomes
Source: PLoS Comput Biol. 2013 Jan 31;9(1):e1002893. doi: 10.1371/journal.pcbi.1002893 (PMC3561073; doi:10.1371/journal.pcbi.1002893)
Supplement: Table S17 — Applying the two-step procedure to the subset of real Hi-C data (equally split one chromosome into two halves), treat each topological domain as an individual unit. The RMSD between two 3D chromosomal structures BACH predicted in the two stages, and , from 20 mouse chromosomes in both HindIII sample and NcoI sample. The tail probabilities < = 0.05 are highlighted in bold font. (DOCX) [file pcbi.1002893.s029.docx]

**Table S17. Applying the two-step procedure to the subset of real Hi-C data (equally split one chromosome into two halves), treat each topological domain as an individual unit.** The RMSD between two 3D chromosomal structures BACH predicted in the two stages, $S_{1}$ and $S_{2}$, from 20 mouse chromosomes in both HindIII sample and NcoI sample. The tail probabilities <= 0.05 are highlighted in bold font.

|  |  |  |  |  |  |
| --- | --- | --- | --- | --- | --- |
|  |  | The HindIII sample | | The NcoI sample | |
| Chromosome | Two halves | RMSD | Tail probability | RMSD | Tail probability |
| 1 | Left half | 0.1131 | 0.065 | 0.1007 | **0.015** |
| 1 | Right half | 0.1902 | 0.809 | 0.1576 | 0.463 |
| 2 | Left half | 0.1416 | 0.312 | 0.1240 | 0.134 |
| 2 | Right half | 0.1075 | **0.034** | 0.1167 | 0.083 |
| 3 | Left half | 0.1797 | 0.699 | 0.1844 | 0.745 |
| 3 | Right half | 0.1441 | 0.282 | 0.1421 | 0.258 |
| 4 | Left half | 0.1645 | 0.544 | 0.1299 | 0.160 |
| 4 | Right half | 0.1187 | 0.094 | 0.1112 | 0.051 |
| 5 | Left half | 0.1596 | 0.480 | 0.1447 | 0.306 |
| 5 | Right half | 0.1213 | 0.105 | 0.1233 | 0.122 |
| 6 | Left half | 0.1551 | 0.449 | 0.1310 | 0.185 |
| 6 | Right half | 0.1366 | 0.236 | 0.1543 | 0.435 |
| 7 | Left half | 0.1468 | 0.342 | 0.1641 | 0.545 |
| 7 | Right half | 0.1587 | 0.478 | 0.1558 | 0.447 |
| 8 | Left half | 0.1739 | 0.641 | 0.1736 | 0.636 |
| 8 | Right half | 0.1441 | 0.283 | 0.1643 | 0.533 |
| 9 | Left half | 0.1171 | 0.082 | 0.1204 | 0.098 |
| 9 | Right half | 0.1195 | 0.095 | 0.1422 | 0.296 |
| 10 | Left half | 0.1892 | 0.800 | 0.1843 | 0.745 |
| 10 | Right half | 0.1475 | 0.295 | 0.1510 | 0.324 |
| 11 | Left half | 0.1665 | 0.575 | 0.1446 | 0.305 |
| 11 | Right half | 0.1556 | 0.435 | 0.1281 | 0.145 |
| 12 | Left half | 0.1321 | 0.188 | 0.1501 | 0.365 |
| 12 | Right half | 0.1454 | 0.317 | 0.1791 | 0.717 |
| 13 | Left half | 0.1113 | **0.039** | 0.1374 | 0.212 |
| 13 | Right half | 0.1784 | 0.703 | 0.1801 | 0.721 |
| 14 | Left half | 0.1359 | 0.199 | 0.1223 | 0.099 |
| 14 | Right half | 0.1613 | 0.478 | 0.1894 | 0.804 |
| 15 | Left half | 0.1694 | 0.579 | 0.1083 | **0.039** |
| 15 | Right half | 0.1444 | 0.282 | 0.1616 | 0.495 |
| 16 | Left half | 0.1915 | 0.818 | 0.1708 | 0.616 |
| 16 | Right half | 0.1554 | 0.414 | 0.2006 | 0.878 |
| 17 | Left half | 0.1162 | 0.065 | 0.1572 | 0.432 |
| 17 | Right half | 0.1319 | 0.159 | 0.1589 | 0.458 |
| 18 | Left half | 0.1778 | 0.686 | 0.1837 | 0.749 |
| 18 | Right half | 0.1633 | 0.526 | 0.1819 | 0.732 |
| 19 | Left half | 0.1255 | 0.105 | 0.1335 | 0.167 |
| 19 | Right half | 0.1439 | 0.258 | 0.1409 | 0.231 |
| X | Left half | 0.1450 | 0.283 | 0.1462 | 0.294 |
| X | Right half | 0.1280 | 0.143 | 0.1577 | 0.450 |
|  |  |  |  |  |  |
