# Supplementary material for: Bayesian Inference of Spatial Organizations of Chromosomes
Source: PLoS Comput Biol. 2013 Jan 31;9(1):e1002893. doi: 10.1371/journal.pcbi.1002893 (PMC3561073; doi:10.1371/journal.pcbi.1002893)
Supplement: Table S18 — The RMSD between the 3D chromosomal structures inferred from the subset of Hi-C contact matrices (equally split one chromosome into two halves) and the 3D chromosomal structures inferred from the original Hi-C contact matrices. The tail probabilities < = 0.05 are highlighted in bold font. (DOCX) [file pcbi.1002893.s030.docx]

**Table S18. The RMSD between the 3D chromosomal structures inferred from the subset of Hi-C contact matrices (equally split one chromosome into two halves) and the 3D chromosomal structures inferred from the original Hi-C contact matrices.** The tail probabilities <= 0.05 are highlighted in bold font.

|  |  |  |  |  |
| --- | --- | --- | --- | --- |
| Left half | The HindIII sample | | The NcoI sample | |
| Chromosome | RMSD | Tail probability | RMSD | Tail probability |
| 1 | 0.0788 | **0.000** | 0.0834 | **0.001** |
| 2 | 0.0737 | **0.000** | 0.0746 | **0.000** |
| 3 | 0.0452 | **0.000** | 0.0870 | **0.002** |
| 4 | 0.0258 | **0.000** | 0.1007 | **0.021** |
| 5 | 0.0853 | **0.006** | 0.0899 | **0.007** |
| 6 | 0.0497 | **0.000** | 0.1057 | **0.034** |
| 7 | 0.1301 | 0.183 | 0.1251 | 0.139 |
| 8 | 0.1107 | **0.049** | 0.1091 | **0.037** |
| 9 | 0.0854 | **0.001** | 0.0165 | **0.000** |
| 10 | 0.0857 | **0.003** | 0.0618 | **0.000** |
| 11 | 0.0869 | **0.004** | 0.1169 | 0.070 |
| 12 | 0.0344 | **0.000** | 0.0915 | **0.007** |
| 13 | 0.0953 | **0.012** | 0.0577 | **0.000** |
| 14 | 0.1059 | **0.023** | 0.0574 | **0.000** |
| 15 | 0.0438 | **0.000** | 0.1029 | **0.026** |
| 16 | 0.1465 | 0.303 | 0.0420 | **0.000** |
| 17 | 0.1000 | **0.022** | 0.0999 | **0.022** |
| 18 | 0.0660 | **0.000** | 0.0907 | **0.005** |
| 19 | 0.1029 | **0.031** | 0.0986 | **0.020** |
| X | 0.0361 | **0.000** | 0.0872 | **0.004** |
|  |  |  |  |  |

|  |  |  |  |  |
| --- | --- | --- | --- | --- |
| Right half | The HindIII sample | | The NcoI sample | |
| Chromosome | RMSD | Tail probability | RMSD | Tail probability |
| 1 | 0.0729 | **0.000** | 0.1092 | **0.038** |
| 2 | 0.0888 | **0.002** | 0.0743 | **0.000** |
| 3 | 0.1044 | **0.021** | 0.0899 | **0.003** |
| 4 | 0.0437 | **0.000** | 0.0754 | **0.000** |
| 5 | 0.0411 | **0.000** | 0.1043 | **0.030** |
| 6 | 0.1094 | **0.048** | 0.0774 | **0.000** |
| 7 | 0.0912 | **0.009** | 0.1203 | 0.111 |
| 8 | 0.0846 | **0.000** | 0.0402 | **0.000** |
| 9 | 0.0630 | **0.000** | 0.1089 | **0.042** |
| 10 | 0.1098 | **0.038** | 0.1095 | **0.036** |
| 11 | 0.1085 | **0.034** | 0.0882 | **0.004** |
| 12 | 0.1445 | 0.304 | 0.0549 | **0.000** |
| 13 | 0.0569 | **0.000** | 0.0583 | **0.000** |
| 14 | 0.1073 | **0.028** | 0.1124 | **0.045** |
| 15 | 0.1272 | 0.136 | 0.1408 | 0.251 |
| 16 | 0.0674 | **0.000** | 0.1616 | 0.492 |
| 17 | 0.0712 | **0.000** | 0.0755 | **0.001** |
| 18 | 0.1398 | 0.283 | 0.1591 | 0.481 |
| 19 | 0.0982 | **0.019** | 0.1369 | 0.193 |
| X | 0.0659 | **0.000** | 0.0962 | **0.021** |
|  |  |  |  |  |
