# Supplementary material for: Bayesian Inference of Spatial Organizations of Chromosomes
Source: PLoS Comput Biol. 2013 Jan 31;9(1):e1002893. doi: 10.1371/journal.pcbi.1002893 (PMC3561073; doi:10.1371/journal.pcbi.1002893)
Supplement: Text S1 — Description of the computational protocol [10]. (DOCX) [file pcbi.1002893.s031.docx]

**Supplementary Text S1**

**Bayesian Inference of Spatial Organizations of Chromosomes**

**Ming Hu, Ke Deng, Zhaohui Qin, Jesse Dixon, Siddarth Selvaraj, Jennifer Fang, Bing Ren**

**& Jun S. Liu***

*corresponding author

**1. Three-stage statistical inference procedure used in the BACH algorithm**

We develop a three-stage procedure to solve the statistical inference problem in the BACH algorithm (Figure S10).

**1.1 Stage 1: Poisson regression**

In the first stage, we aim to assign initial values for the nuisance parameters $\boldsymbol{\beta}=(\beta_{0}, \beta_{1},\beta_{enz},\beta_{gcc},\beta_{map})$. We first set the initial value for $\beta_{1}$ to be $\beta_{1}^{initial}=-1$ since the number of paired-end read spanning two loci $u_{ij}$ and the corresponding spatial distance $d_{ij}$ are negatively correlated [1]. We then fit a Poisson regression model [2] without spatial distances $d_{ij}$ to obtain the initial values for $\beta_{0}$, $\beta_{enz}$, $\beta_{gcc}$ and $\beta_{map}$. The details of the performance and interpretation of this Poisson regression model can be found in an independent technical report [3].

$$u_{ij}\sim Poisson\left( \theta_{ij} \right), 1\leq i<j\leq n ,$$

$$\log\left( \theta_{ij} \right)=\beta_{0}+\beta_{enz}log\left( e_{i}e_{j} \right)+\beta_{gcc}log\left( g_{i}g_{j} \right)+\beta_{map}\log\left( m_{i}m_{j} \right).$$

Let $\beta_{0}^{initial}$, $\beta_{enz}^{initial}$, $\beta_{gcc}^{initial}$ and $\beta_{map}^{initial}$ be the estimated coefficients for $\beta_{0}$, $\beta_{enz}$, $\beta_{gcc}$ and $\beta_{map}$, respectively. We use $\boldsymbol{\beta}^{\boldsymbol{initial}}=(\beta_{0}^{initial}, \beta_{1}^{initial},\beta_{enz}^{initial},\beta_{gcc}^{initial},\beta_{map}^{initial})$ to represent the initial values for all nuisance parameters.

**1.2 Stage 2: sequential importance sampling**

**1.2.1 General framework**

In the second stage, the goal is to obtain an initial 3D chromosomal structure $\boldsymbol{P}^{\boldsymbol{initial}}$ with fixed nuisance parameters $\boldsymbol{\beta}^{\boldsymbol{initial}}$, i.e., sampling from target distribution $P(\boldsymbol{P}|\boldsymbol{U})\propto\prod_{1\leq i<j\leq n} e^{-\theta_{ij}}\theta_{ij}^{u_{ij}}$. However, the target distribution $P(\boldsymbol{P}|\boldsymbol{U)}$ is challenging to directly sample from due to its high dimensionality (number of data points: $n(n-1)/2$, number of parameters: $(3n-6)$). To achieve this goal, we apply sequential importance sampling (SIS) [4] to draw samplers from this high dimensional distribution.

Without loss of generality, we add several constraints on the Cartesian coordinates of the first four loci. We set $x_{1}=y_{1}=z_{1}=0$, $x_{2}\geq0$, $y_{2}=z_{2}=0$, $x_{3}\geq0$, $y_{3}\geq0$, $z_{3}=0$ and $z_{4}\geq0$. Let $\boldsymbol{P}_{\boldsymbol{t}}=\left( P_{1},\ldots,P_{t} \right)^{T}$ represent the collection of Cartesian coordinates for the first $t$ loci, and let $\boldsymbol{U}_{\boldsymbol{t}}={\{u_{ij}\}}_{1\leq i<j\leq t}$ represent the first $t$ rows and $t$ columns of the upper-triangular count matrix $\boldsymbol{U}$. We define a series of bridging distributions $\pi_{t}=\pi_{t}\left( \boldsymbol{P}_{\boldsymbol{t}} | \boldsymbol{U}_{\boldsymbol{t}} \right)$,$t=1,\ldots,n$, in which $\pi_{n}$ is the target distribution $P(\boldsymbol{P}|\boldsymbol{U)}$. Let $\left\{ \boldsymbol{P}_{\boldsymbol{t-1}}^{\boldsymbol{k}}\boldsymbol{,}w_{t-1}^{k} \right\}_{1\leq k\leq K}$ be $K$ weighted samples from $\pi_{t-1}$ ($w_{1}^{k}=1, k=1,\ldots,K$). For each sample $\boldsymbol{P}_{\boldsymbol{t-1}}^{\boldsymbol{k}}$, we draw $P_{t}$ from a proposal distribution $f_{t}=f_{t}(P_{t}|\boldsymbol{P}_{\boldsymbol{t-1}}^{\boldsymbol{k}})$, and then $\boldsymbol{P}_{\boldsymbol{t}}^{\boldsymbol{k}}=(\boldsymbol{P}_{\boldsymbol{t-1}}^{\boldsymbol{k}},P_{t}^{k})$ forms a new sample from $\pi_{t}$ with weight $w_{t}^{k}$ as following:

$$w_{t}^{k}=\frac{\pi_{t}^{k}}{f_{t}f_{t-1}\ldots f_{2}f_{1}}=w_{t-1}^{k}\frac{\pi_{t}^{k}}{\pi_{t-1}^{k}f_{t}} , k=1,\ldots,K.$$

At the end of sequential importance sampling, we are able to obtain $K$ weighted 3D chromosomal structures $\left\{ \boldsymbol{P}_{\boldsymbol{n}}^{\boldsymbol{k}}\boldsymbol{,}w_{n}^{k} \right\}_{1\leq k\leq K}$ with respect to the target distribution $P(\boldsymbol{P}|\boldsymbol{U)}$, in which the structure with the highest likelihood is selected as the initial 3D chromosomal structure $\boldsymbol{P}^{\boldsymbol{initial}}$.

**1.2.2 Design of proposal distribution**

In sequential importance sampling procedure, the proposal distribution $f_{t}$ is directly related to the sampling efficiency. We define the proposal distribution as $f_{t}=f_{t}(P_{t}|P_{t-i},1\leq i\leq4)$, i.e., the Cartesian coordinates of any locus in 3D space are uniquely determined by its spatial distance to any other four loci. Furthermore, the bridging distribution $\pi_{t}$ is a product of multiple Poisson densities which only depends on the corresponding spatial distances. Therefore, we define the proposal distribution for $P_{t}$ in spherical coordinate system with origin at $P_{t-1}=\left( x_{t-1},y_{t-1},z_{t-1} \right)^{T}$, and then draw radius $d_{t,t-1}$ and two angles $(\psi_{t},\phi_{t})$ independently. To be specific, the transformation between two coordinate systems is of the form:

$$x_{t}=x_{t-1}+d_{t,t-1}sin\left( \psi_{t} \right)cos\left( \phi_{t} \right) ,$$

$$y_{t}=y_{t-1}+d_{t,t-1}sin\left( \psi_{t} \right)sin\left( \phi_{t} \right) ,$$

$$z_{t}=z_{t-1}+d_{t,t-1}cos\left( \psi_{t} \right) ,$$

$$d_{t,t-1}\in\left[ 0,+\infty\right) , \psi_{t}\in\left[ 0,\pi\right) , \phi_{t}\in\left[ 0, 2\pi\right) .$$

We first sample $\theta_{t,t-1}$ from $\Gamma(1,u_{t,t-1}+1)$, and then calculate $d_{t,t-1}$by:

$$d_{t,t-1}=\exp\left\{ \left( \log\theta_{t,t-1}-\beta_{0}-\beta_{enz}log\left( e_{i}e_{j} \right)-\beta_{gcc}log\left( g_{i}g_{j} \right)-\beta_{map}\log\left( m_{i}m_{j} \right) \right)/\beta_{1} \right\} .$$

Therefore,

$$f_{t}\left( d_{t,t-1} | P_{t-i},1\leq i\leq4 \right)=\frac{e^{-\theta_{t,t-1}}\theta_{t,t-1}^{u_{t,t-1}}}{u_{t,t-1}!}\frac{\theta_{t,t-1}|\beta_{1}|}{d_{t,t-1}} .$$

Next we equally divide $[0, \pi)$ and $[0, 2\pi)$ into ten consecutive and disjoint intervals with equal size, and then use a 100-dimensional multinomial distribution $\hat{f}_{t}(\psi_{t},\phi_{t}|d_{t,t-1},P_{t-i},1\leq i\leq4)$ to approximate:

$$f_{t}\left( \psi_{t},\phi_{t} | d_{t,t-1},P_{t-i},1\leq i\leq4 \right)\propto\prod_{i=1}^{4} e^{-\theta_{t,t-i}}\theta_{t,t-i}^{u_{t,t-i}} .$$

Combining the proposal distribution of $d_{t,t-1}$ and $(\psi_{t},\phi_{t})$ with the corresponding Jacobian $1/d_{t,t-1}^{2}\sin(\psi_{t})$, we obtain the following joint proposal distribution in the Cartesian coordinate system:

$$f_{t}=f_{t}\left( P_{t} | P_{t-i},1\leq i\leq4 \right)=\frac{e^{-\theta_{t,t-1}}\theta_{t,t-1}^{u_{t,t-1}}}{u_{t,t-1}!}\frac{\theta_{t,t-1}\left| \beta_{1} \right|}{d_{t,t-1}^{3}sin(\psi_{t})}\hat{f}_{t}\left( \psi_{t},\phi_{t} | d_{t,t-1},P_{t-i},1\leq i\leq4 \right) .$$

The increment of weight in the Cartesian coordinate system is defined as following:

$$\frac{\pi_{t}}{\pi_{t-1}f_{t}}=\frac{\prod_{i=1}^{t-2} e^{-\theta_{t,i}}\theta_{t,i}^{u_{t,i}}}{\hat{f}_{t}(\psi_{t},\phi_{t}|d_{t,t-1},P_{t-i},1\leq i\leq4)}\frac{d_{t,t-1}^{3}sin(\psi_{t})}{\theta_{t,t-1}|\beta_{1}|} .$$

**1.2.3 Rejection control technique**

To reduce the variance of the weight and improve the efficiency of sequential importance sampling, we use the rejection control technique [5,6]. Suppose we have $K$ weighted structures $\left\{ \boldsymbol{P}_{\boldsymbol{t-1}}^{\boldsymbol{k}}\boldsymbol{,}w_{t-1}^{k} \right\}_{1\leq k\leq K}$ for loci $L_{1}$,…, $L_{t-1}$ at the $t-1$ th step in sequential importance sampling. For each structure $\boldsymbol{P}_{\boldsymbol{t-1}}^{\boldsymbol{k}}$, we draw $S$ new locations $\left\{ P_{t}^{ks} \right\}_{1\leq s\leq S}$ for locus $L_{t}$, and define $\boldsymbol{P}_{\boldsymbol{t}}^{\boldsymbol{ks}}=(\boldsymbol{P}_{\boldsymbol{t-1}}^{\boldsymbol{k}},P_{t}^{ks})$.$\left\{ \boldsymbol{P}_{\boldsymbol{t}}^{\boldsymbol{ks}}\boldsymbol{,}w_{t}^{ks} \right\}_{1\leq k\leq K,1\leq s\leq S}$ represent $KS$ weighted structures for loci $L_{1}$,…, $L_{t}$ at the $t$ th step in sequential importance sampling. Next we solve the following equation to obtain a cutoff value $c$:

$$\sum_{k=1}^{K} \sum_{s=1}^{S} min\left( \frac{w_{t}^{ks}}{c},1 \right)=K .$$

We keep each weighted structure $\{\boldsymbol{P}_{\boldsymbol{t}}^{\boldsymbol{ks}}\boldsymbol{,}w_{t}^{ks}\}$ with probability $min\left( {w_{t}^{ks}}/c,1 \right)$, and the expected number of weighted structures is $K$ after this filter.

**1.3 Stage 3: Gibbs sampler**

**1.3.1 General framework**

In the third stage, we use Gibbs sampler on the joint posterior distribution $P\left( \boldsymbol{P},\boldsymbol{\beta} | \boldsymbol{U} \right)$ to iteratively refine the initial values of the nuisance parameters $\boldsymbol{\beta}^{\boldsymbol{initial}}$ and the initial 3D chromosomal structure $\boldsymbol{P}^{\boldsymbol{initial}}$ obtained from the first and the second stages. The conditional distributions for the nuisance parameters are all log concave; therefore we use adaptive rejection sampling (ARS) [7] to iteratively sample them from the corresponding conditional distribution. However, it is challenging to refine the 3D chromosomal structure since the standard Gibbs sampler, which only updates the Cartesian coordinates of one locus at each time, can easily be trapped into local modes. To achieve this goal, we use hybrid Monte Carlo [8,9] to update the Cartesian coordinates of all loci jointly.

**1.3.2 Updating nuisance parameters using adaptive rejection sampling**

The log likelihood of the conditional distributions forthe nuisance parameters $\boldsymbol{\beta}$ are of the form:

$$\log P\left( \beta_{0} | \boldsymbol{P},\beta_{1},\beta_{enz},\beta_{gcc},\beta_{map},\boldsymbol{U} \right)=\sum_{1\leq i<j\leq n} -\exp\left\{ \beta_{0}+\beta_{1}\log\left( d_{ij} \right)+\beta_{enz}log\left( e_{i}e_{j} \right)+\beta_{gcc}log\left( g_{i}g_{j} \right)+\beta_{map}\log\left( m_{i}m_{j} \right) \right\}+\beta_{0}\sum_{1\leq i<j\leq n} u_{ij} ,$$

$$\log P(\beta_{1}|\boldsymbol{P},\beta_{0},\beta_{enz},\beta_{gcc},\beta_{map},\boldsymbol{U})=\sum_{1\leq i<j\leq n} -\exp\left\{ \beta_{0}+\beta_{1}\log\left( d_{ij} \right)+\beta_{enz}log\left( e_{i}e_{j} \right)+\beta_{gcc}log\left( g_{i}g_{j} \right)+\beta_{map}\log\left( m_{i}m_{j} \right) \right\}+\beta_{1}\sum_{1\leq i<j\leq n} u_{ij}log(d_{ij}) ,$$

$$\log P(\beta_{enz}|\boldsymbol{P},\beta_{0},\beta_{1},\beta_{gcc},\beta_{map},\boldsymbol{U})=\sum_{1\leq i<j\leq n} -\exp\left\{ \beta_{0}+\beta_{1}\log\left( d_{ij} \right)+\beta_{enz}log\left( e_{i}e_{j} \right)+\beta_{gcc}log\left( g_{i}g_{j} \right)+\beta_{map}\log\left( m_{i}m_{j} \right) \right\}+\beta_{enz}\sum_{1\leq i<j\leq n} u_{ij}log\left( e_{i}e_{j} \right) ,$$

$$\log P(\beta_{gcc}|\boldsymbol{P},\beta_{0},\beta_{1},\beta_{enz},\beta_{map},\boldsymbol{U})=\sum_{1\leq i<j\leq n} -\exp\left\{ \beta_{0}+\beta_{1}\log\left( d_{ij} \right)+\beta_{enz}log\left( e_{i}e_{j} \right)+\beta_{gcc}log\left( g_{i}g_{j} \right)+\beta_{map}\log\left( m_{i}m_{j} \right) \right\}+\beta_{gcc}\sum_{1\leq i<j\leq n} u_{ij}log\left( g_{i}g_{j} \right) ,$$

$$\log P(\beta_{map}|\boldsymbol{P},\beta_{0},\beta_{1},\beta_{enz},\beta_{gcc},\boldsymbol{U})=\sum_{1\leq i<j\leq t} -\exp\left\{ \beta_{0}+\beta_{1}\log\left( d_{ij} \right)+\beta_{enz}log\left( e_{i}e_{j} \right)+\beta_{gcc}log\left( g_{i}g_{j} \right)+\beta_{map}\log\left( m_{i}m_{j} \right) \right\}+\beta_{map}\sum_{1\leq i<j\leq t} u_{ij}\log\left( m_{i}m_{j} \right) .$$

**1.3.3 Updating 3D chromosomal structure using hybrid Monte Carlo**

Hybrid Monte Carlo integrates a random walk type Metropolis Monte Carlo move with semi-deterministic proposals through Hamiltonian dynamics of a many-body system in which the potential function is the target density. It enables the sampler to move across the sample space in larger steps and therefore the MCMC chain converges and mixes more rapidly. Computational overheads of hybrid Monte Carlo include the computation of the first order partial derivatives with respect to $P_{i}=\left( x_{i},y_{i},z_{i} \right)^{T}:$

$$\frac{\partial logP(\boldsymbol{P}|\boldsymbol{\beta},\boldsymbol{U})}{\partial x_{i}}=\sum_{1\leq j\leq n,j\neq i} \left( u_{ij}-\theta_{ij} \right)\beta_{1}\frac{x_{i}-x_{j}}{d_{ij}^{2}} ,$$

$$\frac{\partial logP(\boldsymbol{P}|\boldsymbol{\beta},\boldsymbol{U})}{\partial y_{i}}=\sum_{1\leq j\leq n,j\neq i} \left( u_{ij}-\theta_{ij} \right)\beta_{1}\frac{y_{i}-y_{j}}{d_{ij}^{2}} ,$$

$$\frac{\partial logP(\boldsymbol{P}|\boldsymbol{\beta},\boldsymbol{U})}{\partial z_{i}}=\sum_{1\leq j\leq n,j\neq i} \left( u_{ij}-\theta_{ij} \right)\beta_{1}\frac{z_{i}-z_{j}}{d_{ij}^{2}} .$$

The tuning parameters controlling step sizes in random walk type Metropolis Monte Carlo are directly related to the efficiency of hybrid Monte Carlo. We adaptively update the tuning parameters to control the acceptance rate of the Metropolis sampler in a reasonable range (70%~90%).

**1.4 Normalization of the scale**

The 3D chromosomal structure BACH predicted is scale free, i.e., the scale parameter $\beta_{0}$ is not identifiable with the 3D chromosomal structure $\boldsymbol{P}$ under any similarity transformation. To make $\beta_{0}$ identifiable, we impose the constraint ($d_{1n}=1$) on the spatial distance between the first locus $L_{1}$ and the last locus $L_{n}$.

**2.** **Validation of inferred spatial distances by FISH experiment**

In a recent study on the mESC [10], eleven 40 KB FISH probes (Table S8) were designed for eleven genes of interest, and the spatial distances between six probe pairs were measured in the FISH experiment (Table S9). According to the known topological domain annotations [11], we found that these six probe pairs belong to four topological domains (Figure S2A and Table S10). In both HindIII sample and NcoI sample, we applied BACH to these four topological domains jointly to infer the corresponding 3D chromosomal structures (Figure S2**)**.

**3. A modified BACH algorithm without bias correction**

**3.1 Comparison with the FISH distances**

Since MCMC5C does not remove systematic biases (restriction enzyme cutting frequencies, GC content and sequence uniqueness), we also applied a modified BACH algorithm without bias correction (denoted as BACH-SUB) and obtained the corresponding predictions of spatial distances (referred to as the BACH-SUB distances). The Pearson correlation coefficients between the BACH-SUB distances and the FISH distance are 0.87 (95% credible interval is [0.81, 0.92]) and 0.18 (95% credible interval is [0.02, 0.30]) in the HindIII sample and the NcoI sample, respectively. BACH-SUB significantly outperforms MCMC5C in the HindIII sample (MCMC5C: 0.79, z-test p-value = 0.0004), and is comparable with MCMC5C in the NcoI sample (MCMC5C: 0.11, z-test p-value = 0.1669). These results suggest that the Poisson model used in the BACH algorithm fits the count data of the Hi-C experiment better than the Gaussian model used in MCMC5C.

**3.2 Whole chromosome modeling**

We used BACH-SUB to generate spatial models of each long chromosome (chr 1 to chr 14 and chr X) by treating each topological domain as an individual unit (Figure S7). BACH-SUB achieved a significantly higher reproducibility (measured by the normalized root mean square deviations, i.e., RMSD, Methods) than those predicted by MCMC5C (paired t-test p-value = 0.0465). Since both BACH-SUB and MCMC5C do not remove systematic biases and the major difference between these two methods is the different distribution (Poisson distribution vs. Gaussian distribution), the improvement of BACH-SUB over MCMC5C is likely due to that the Poisson model used in BACH fits the count data of the Hi-C experiment better than the Gaussian model used in MCMC5C.

**4. Simulation studies**

**4.1 Simulation study for the BACH algorithm**

We conducted a simulation study to access the effectiveness of the BACH algorithm. In literature, FISH data supports the random walk backbone model for 3D chromosomal structures [12,13], therefore, we used a random walk scheme to generate a hypothetical 3D chromosomal structure (red dots and red lines in Figure S11A) with 33 loci (each locus represents a 1 MB genomic region). The differences of Cartesian coordinates between any two adjacent loci $t-1$ and $t$, $(x_{t}-x_{t-1},y_{t}-y_{t-1},z_{t}-z_{t-1})$, were sampled independently from normal distribution $N(0,1)$, i.e., $P_{t}=P_{t-1}+\varepsilon_{t}$, $t=1,\ldots,33$ where $\varepsilon_{t} iid\sim Normal(0,I_{3})$. To make the 3D chromosomal structure identifiable up to the scaling parameter, we set the spatial distance between the first locus and the last locus to be one. The local genomic features $e_{i}$, $g_{i}$ and $m_{i}$ were obtained from the human chromosome 22 with restriction enzyme HindIII at the 1 MB resolution. We further set the nuisance parameter $\beta_{0}$, $\beta_{1}$, $\beta_{enz}$, $\beta_{gcc}$ and $\beta_{map}$ to be $4$, $-1$, $0.1$, $-0.1$ and $0.1$, respectively. The contact matrix $\boldsymbol{U}=\left\{ u_{ij} \right\}_{1\leq i,j\leq33}$ was simulated from the posited model. We implemented the BACH algorithm with the default settings. The Gelman-Rubin statistic of three parallel chains was 1.0050, which indicates all chains converge to the same posterior distribution (Figure S11B). Among three parallel chains, we selected the posterior samples (after burn-in and thin) from the chain that achieved the highest log likelihood (Figure S11B and Figure S11C) for posterior inference. The 3D chromosomal structures BACH predicted (white dots and white lines in Figure S11A) resembled closely to the original simulated 3D chromosomal structure with the normalized RMSD 0.0104. The posterior mean and 95% credible interval for parameters were reported in Table S11. The 95% credible intervals of $\beta_{0}$, $\beta_{1}$, $\beta_{enz}$, $\beta_{gcc}$ and $\beta_{map}$ all covered the corresponding true values. These results demonstrate that BACH is able to provide accurate spatial distance estimates when applying to the data simulated from the posited model with single consensus 3D chromosomal structure.

**4.2. Simulation study for the BACH-MIX algorithm**

We then conducted a simulation study to test the performance the BACH-MIX algorithm. We first applied BACH to the 1 MB resolution level Hi-C contact matrix of the human chromosome 22 (33 loci) in a human lymphoblastic cell line with restriction enzyme HindIII [1]. The 3D chromosomal structure BACH predicted was listed in Figure S12A. We then equally divided the chromosome 22 into two adjacent genomic regions: genomic region $A$ and genomic region $B$ (Figure S12A). A 12 dimensional multinomial distribution $p_{\Theta}$ was used to approximate $\pi\left( \Theta\right)$ (Table S12). The contact matrix $\boldsymbol{U}^{\boldsymbol{mix}}=\left\{ u_{ij} \right\}_{1\leq i\leq16,2\leq j\leq17}$ was simulated from the posited model. We implemented the BACH-MIX algorithm with the default settings. Since there are only 11 unknown parameters in this simulation study, we did not apply the two-step procedure to avoid over-fitting problem. The Gelman-Rubin statistic of three parallel chains was 1.0011, which indicates all chains converge to the same posterior distribution (Figure S12B). Among three parallel chains, we selected the posterior samples (after burn-in and thin) from the chain that achieved the highest log likelihood (Figure S12B and Figure S12C) for posterior inference. The posterior mean and 95% credible interval for parameters were reported in Table S12 and Figure S12D. The 95% credible intervals of 12 parameters all covered the corresponding true values. These results demonstrate that BACH-MIX is able to accurately characterize the structure variations of chromatin when applying to the data simulated from the posited model with multiple distinct 3D chromosomal structures.

**4.3 Simulation study for the BACH algorithm when the input Hi-C contact matrix is simulated from a mixture population**

We conduct a series of simulation studies to evaluate the performance of the BACH algorithm when the input Hi-C contact matrix is simulated from a mixture population. Similar to the previous simulation study, we used a random walk scheme to generate two hypothetical 3D chromosomal structures $A$ and $B$, each with 33 loci (each locus represents a 1 MB genomic region). The differences of Cartesian coordinates between any two adjacent loci $t-1$ and $t$, $(x_{t}-x_{t-1},y_{t}-y_{t-1},z_{t}-z_{t-1})$, were sampled independently from normal distribution $N(0,1)$, i.e., $P_{t}=P_{t-1}+\varepsilon_{t}$, $t=1,\ldots,33$ where $\varepsilon_{t} iid\sim Normal(0,I_{3})$. To make the 3D chromosomal structure identifiable up to the scaling parameter, we set the spatial distance between the first locus and the last locus to be one. The local genomic features $e_{i}$, $g_{i}$ and $m_{i}$ were obtained from the human chromosome 22 with restriction enzyme HindIII at the 1 MB resolution. We further set the nuisance parameter $\beta_{0}$, $\beta_{1}$, $\beta_{enz}$, $\beta_{gcc}$ and $\beta_{map}$ to be $4$, $-1$, $0.1$, $-0.1$ and $0.1$, respectively.

For two simulated 3D chromosomal structures $A$ and $B$, we converted the pairwise spatial distances $d_{ij}^{A}$ and $d_{ij}^{B}$ between any two loci $i$ and $j$ to the Poisson rates $\theta_{ij}^{A}$ and $\theta_{ij}^{B}$ using the posited Poisson model. We define $\pi$ ($\pi\geq0.5$) as the mixture proportion of the dominant 3D chromosomal structure $A$, and simulate the contact matrix $\boldsymbol{U}=\left\{ u_{ij} \right\}_{1\leq i,j\leq33}$ from the Poisson distribution, where the Poisson rate $\theta_{ij}$ is defined as $\pi\theta_{ij}^{A}+(1-\pi)\theta_{ij}^{B}$. In this simulation setting, the contact matrix is simulated from a mixture population of two components $A$ and $B$.

We applied the following two-step inference procedure for each simulated contact matrix $\boldsymbol{U}$. In the first step, we applied BACH to the simulated contact matrix $\boldsymbol{U}$ and obtained the first BACH predicted 3D chromosomal structure $S_{1}$ and the expected Hi-C contact matrix $\hat{\theta}_{ij}$. We defined the residual matrix$\boldsymbol{RES}=\left\{ {res}_{ij} \right\}_{1\leq i,j\leq33}$ as $res_{ij}=max\left( \left[ u_{ij}-0.5*\hat{\theta}_{ij} \right],0 \right)$. In the second step, we applied BACH to the residual matrix $\boldsymbol{RES}$ and obtained the second BACH predicted 3D chromosomal structure $S_{2}$.

For five different values of mixture proportion $\pi$ ($\pi=0.5, 0.6,0.7,0.8, 0.9$), we repeated the simulation procedure the two-step inference procedure 100 times. In each simulation, we calculated the similarities between two BACH predictions and two simulated structures, which are measured by four RMSDs: RMSD($S_{1}$, $A$), RMSD($S_{1}$, $B$), RMSD($S_{2}$, $A$), RMSD($S_{2}$, $B$). We also calculated the similarity between two BACH predictions RMSD($S_{1}$,$S_{2}$), and between two simulated structures RMSD($A$,$B$). Since $A$ and $B$ are both simulated from the random walk scheme, RMSD($A$,$B$)s obtained from 100 simulations are the empirical distribution of RMSDs between two random structures.

Figure S4 lists the distribution of six RMSDs obtained from 100 simulations with different mixture proportion $\pi$. Table S5 lists the mean of six RMSDs obtained from 100 simulations with different mixture proportion $\pi$. These results suggest that when the mixture population contains one dominant sub-population (proportion >= 80%), the two 3D chromosomal structures BACH predicted in the two stages, $S_{1}$ and $S_{2}$, show high similarity (low RMSD), and are both close to the 3D chromosomal structure of the dominant sub-population. In contrast, when the mixture population contains two sub-populations with comparable proportions (the dominant sub-population with proportion <= 70%), $S_{1}$ and $S_{2}$ show high discrepancy, and both are different from either of the two underlying simulated 3D chromosomal structures.

**5. Preprocessing procedure of the raw Hi-C data**

**5.1. Mapping reads to the genome**

Hi-C raw reads were downloaded from NCBI (GSE35156), where the restriction enzyme HindIII was used in two replicates with 205,463,135 reads and 270,706,438 reads, and the restriction enzyme NcoI was used in a single replicate with 237,328,485 reads. The reads were 36 bases and paired-end. BWA sample option was used to align paired end reads using default parameters. After alignment each end was checked for unique mapping (read mapping score > 10) and only unique mapped read pairs were kept. Furthermore, PCR duplicates were removed using PICARD default parameters. We only used intra-chromosomal reads, and further removed reads with insert size less than 20 KB, as performed in original Hi-C paper [1].

**5.2. Mappability score**

Each restriction enzyme cutting site had two fragments and the location and size of each fragment were found by scanning the reference genome (mm9) for HindIII (AAGCTT) and NcoI (CCATGG), respectively. To compute the mappability score, we made 55 artificial reads around every fragment (over a 500 bp region) with each read overlapping 9 bps. And we mapped these reads using BWA default parameters and the fraction of reads that mapped uniquely (read mapping score > 10) was defined as the mappability score. We discarded reads that aligned to fragments < 0.5 mappability score.

**5.3. Identification of nonspecific ligation products**

For every paired end read pairs, we checked how far they align to the corresponding fragments starting base (restriction enzyme cutting site) and removed reads where the sum of the distances among read start site and restriction cutting site > 500bp. Yaffe and Tanay [14] defined these as non-specific ligations and we considered only reads with specific ligation for downstream analysis. These data cleaning steps left us with 41,525,121 and 75,903,467 reads for the two libraries with HindIII, and 70,303,160 reads for the library with NcoI. We then pooled the two libraries with HindIII together (referred to as the HindIII sample), and further removed reads with insert size less than 40 KB, finally we obtained 112,207,808 reads for the HindIII sample, and 66,964,713 reads for the NcoI sample.

**6. Details of genomic and epigenetic features**

The enrichment of histone modifications and chromatin binding factors over each domain was calculated as follows. For data from ChIP-sequencing experiments, we first differentiated factors that bind in a “block” like pattern (H3K27me3, H3K9me3, H3K36me3, H4K20me3) and factors that bind as “peaks” (H3K4me3, RNA Polymerase II, DNaseI HS). For “block like” factors, enrichment was calculated as the log base 2 of the ChIP RPKM divided by the input RPKM over the domain. For peak like factors, enrichment was calculated as the frequency of peaks or binding sites over the domain (number of peaks/domain size). For data from ChIP-Chip experiments (Lamin-B1 DamID, replication timing), enrichment was calculated as the average normalized probe signal over each domain. The gene density was calculated by assessing the frequency of unique mouse mm9/NCBI37 RefSeq transcription start sites across each domain (number of TSS/domain size). RNA-seq signal over each topological domain was calculated as the median RNA-Seq RPKM of each gene in a given topological domain.

**7.** **3D chromosomal structure model for the whole chromosome at different resolution scales**

To control for the chromosome size, we conducted the following analyses. We first zoomed in the Hi-C contact matrix of each chromosome by splitting one topological domain into two sub-domains with equal sizes, and then treated each sub-domain as an individual unit. In addition, we zoomed out the Hi-C contact matrix of each chromosome by merging two adjacent topological domains into one super-domain, and then treated each super-domain as an individual unit. We applied the previous two-step procedure to the zoomed-in and zoomed-out Hi-C contact matrices, and reported the RMSD($S_{1}$, $S_{2}$) and corresponding tail probability in Table S13 and Table S14. We found consistent results in zoomed-in and zoomed-out Hi-C contact matrices: RMSD($S_{1}$, $S_{2}$) is small (tail probability <= 0.05) in most of long chromosomes, and large (tail probability > 0.05) in most of short chromosomes. We also aligned the 3D chromosomal structure inferred from the zoomed-in and zoomed-out Hi-C contact matrices to the 3D chromosomal structure inferred from the original Hi-C contact matrices, and found that the 3D chromosomal structures inferred at different resolution scales show high level of similarity (Table S15 and Table S16). Furthermore, we split each chromosome into two halves with equal sizes, and applied the previous two-step procedure to each half chromosome, treating each topological domain as an individual unit. We found that RMSD($S_{1}$, $S_{2}$) is large (tail probability > 0.05) in most of chromosomes (Table S17). In most of chromosomes, the 3D chromosomal structure inferred from the half chromosome aligned well with the 3D chromosomal structure inferred from the whole chromosome (Table S18). To be more conservative, we also implemented a similar two-step procedure, in which the residual matrix is defined as the difference between the original Hi-C contact matrix and one third of the expected Hi-C contact matrix, and obtained consistent results (data not shown). All these results suggest that long chromosomes may exhibit a dominant sub-population in a cell population, and short chromosomes may exhibit multiple distinct sub-populations with comparable mixture proportions in a cell population. These conclusions are consistent at different resolution scales, and are not affected by the resolution of original Hi-C contact matrix.

**8. Visualization of predicted 3D chromosomal structure**

While the predicted 3D chromosomal structure may be visualized using standard statistical packages, these tools do not allow for easy manipulation or annotation of structures. Therefore, we modified the BACH predicted 3D chromosomal structure for visualization in Jmol [15], a free, platform-independent Java-based tool for protein structure visualization. From the BACH predicted 3D chromosomal structure, imitation protein and coordinate connectivity data were generated in Tinker XYZ [16] format and then converted to PDB format, the format accepted by any protein viewer. Using bin information and genomic feature binaries, we generated Jmol scripts to label and color annotations. As a web applet, Jmol allows users to manipulate the 3D chromosomal models real time, with all the same features as the desktop program.

**9. Movies**

Movies of the BACH predicted 3D chromosomal structure of chromosome 2 in the HindIII sample (same as **Figure 3A**). Each sphere represents a topological domain. The volume of the sphere is proportional to the genomic size of the corresponding topological domain. The red, white and blue colors represent topological domains belonging to compartment A, straddle region and compartment B, respectively. Topological domains with the same compartment label tend to locate on the same side of the structure.

**9.1 Movie of the BACH predicted 3D chromosomal structure of chromosome 2 in the HindIII sample. The 3D chromosomal structure spins around the z-axis.**

The movie can be downloaded from:

<http://www.people.fas.harvard.edu/~junliu/BACH/chr2_Hind3_movie_spin_z_axis.wmv>

**9.2 Movie of the BACH predicted 3D chromosomal structure of chromosome 2 in the HindIII sample. The 3D chromosomal structure spins around the x-axis.**

The movie can be downloaded from:

<http://www.people.fas.harvard.edu/~junliu/BACH/chr2_Hind3_movie_spin_x_axis.wmv>

**10. Using 104 spatial arrangements of two adjacent sub-regions to approximate the collection of distinct 3D chromosomal structures in a cell population.**

To simplify the computation in the BACH-MIX algorithm, we discretize the range of each Euler angle into four bins of equal sizes. Specifically, we use $\phi\in\{0,\pi/2,\pi,3\pi/2\}$, $\theta\in\{-\pi/2,-\pi/4,0, \pi/4\}$ and $\psi\in\{0,\pi/2,\pi,3\pi/2\}$. We also take into account mirror symmetry structures that cannot be explained by rotations. The rotation matrix $R(\Theta)$ is defined as:

$$R\left( \Theta\right)=\left[ \begin{matrix} \cos\theta\cos\psi& -\cos\phi\sin\psi+\sin\phi\sin\theta\cos\psi& \sin\phi\sin\psi+\cos\phi\sin\theta\cos\psi\\ \cos\theta\sin\psi& \cos\phi\cos\psi+\sin\phi\sin\theta\sin\psi& -\sin\phi\cos\psi+\cos\phi\sin\theta\sin\psi\\ -\sin\theta& \sin\phi\cos\theta& \cos\phi\cos\theta\end{matrix} \right].$$

Let $I\in\{0, 1\}$ be the mirror symmetry index. The mirror symmetry matrix $M\left( \Theta\right)$ is defined as:

$$M\left( \Theta\right)=\left[ \begin{matrix} 1 & 0 & 0 \\ 0 & 1 & 0 \\ 0 & 0 & \left( -1 \right)^{I} \end{matrix} \right].$$

When $\theta\neq-\pi/2$, it is easy to verify that the combination of three Euler angles $\phi$, $\theta$, $\psi$ and the mirror symmetry index $I$ results in 96 ($4\times3\times4\times2=96)$ distinct rotation matrices $R\left( \Theta\right)M(\Theta)$. These 96 rotation matrices correspond to 96 distinct spatial arrangements of two adjacent sub-regions.

When $\theta=-\pi/2$, the rotation matrix $R\left( \Theta\right)$ is degenerated to :

$$R\left( \Theta\right)=\left[ \begin{matrix} 0 & -\cos\phi\sin\psi-\sin\phi\cos\psi& \sin\phi\sin\psi-\cos\phi\cos\psi\\ 0 & \cos\phi\cos\psi-\sin\phi\sin\psi& -\sin\phi\cos\psi-\cos\phi\sin\psi\\ -1 & 0 & 0 \end{matrix} \right]$$

$$=\left[ \begin{matrix} 0 & -\sin(\phi+\psi) & -\cos(\phi+\psi) \\ 0 & \cos(\phi+\psi) & -\sin(\phi+\psi) \\ -1 & 0 & 0 \end{matrix} \right].$$

In this scenario, $R(\Theta)$ only depends on $\phi+\psi$. Since we use $\phi\in\{0,\pi/2,\pi,3\pi/2\}$ and $\psi\in\{0,\pi/2,\pi,3\pi/2\}$, $\phi+\psi$ takes four distinct values: $0$, $\pi/2$, $\pi$ and $3\pi/2$. Considering the mirror symmetry index $I$, there are 8 ($4\times2=8$) distinct rotation matrices $R\left( \Theta\right)M(\Theta)$. These 8 rotation matrices correspond to 8 distinct spatial arrangements of two adjacent sub-regions.

Combing these two scenarios, we use 104 ($96+8=104$) spatial arrangements of two adjacent sub-regions to approximate the collection of distinct 3D chromosomal structures in a cell population.

**Text S1 References**

1. Lieberman-Aiden E, van Berkum NL, Williams L, Imakaev M, Ragoczy T, et al. (2009) Comprehensive mapping of long-range interactions reveals folding principles of the human genome. Science 326: 289-293.

2. McCullagh P, Nelder JA (1989) Generalized linear models: Chapman & Hall/CRC.

3. Hu M, Deng K, Selvaraj S, Qin Z, Ren B, et al. (2012) HiCNorm: removing biases in Hi-C data via Poisson regression. Bioinformatics: In press.

4. Liu JS, Chen R (1998) Sequential Monte-Carlo Methods For Dynamic-Systems. Journal of the American Statistical Association 93: 1032-1044.

5. Liu JS, Chen R, Wong WH (1998) Rejection Control and Sequential Importance Sampling. Journal of the American Statistical Association 93: 1022-1031.

6. Fearnhead P, Clifford P (2003) On-Line Inference for Hidden Markov Models via Particle Filters. Journal of the Royal Statistical Society Series B (Statistical Methodology) 65: 887-899.

7. Gilks WR, Wild P (1992) Adaptive Rejection Sampling For Gibbs Sampling. Applied Statistics-Journal of the Royal Statistical Society Series C 41: 337-348.

8. Duane S, Kennedy AD, Pendleton BJ, Roweth D (1987) Hybrid Monte-Carlo. Physics Letters B 195: 216-222.

9. Liu J (2001) Monte Carlo Strategies in scientific computing. New York: Springer-Verlag.

10. Eskeland R, Leeb M, Grimes GR, Kress C, Boyle S, et al. (2010) Ring1B compacts chromatin structure and represses gene expression independent of histone ubiquitination. Mol Cell 38: 452-464.

11. Dixon J, Selvaraj S, Yue F, Kim A, Li Y, et al. (2012) Topological domains in mammalian genomes identified by analysis of chromatin interactions. Nature 485: 376-380.

12. Sachs RK, van den Engh G, Trask B, Yokota H, Hearst JE (1995) A random-walk/giant-loop model for interphase chromosomes. Proc Natl Acad Sci U S A 92: 2710-2714.

13. Yokota H, van den Engh G, Hearst JE, Sachs RK, Trask BJ (1995) Evidence for the organization of chromatin in megabase pair-sized loops arranged along a random walk path in the human G0/G1 interphase nucleus. J Cell Biol 130: 1239-1249.

14. Yaffe E, Tanay A (2011) Probabilistic modeling of Hi-C contact maps eliminates systematic biases to characterize global chromosomal architecture. Nat Genet 43: 1059-1065.

15. Jmol: an open-source Java viewer for chemical structures in 3D. <http://www.jmol.org/>

16. Ponder JW, Richards FM (1987) An efficient newton-like method for molecular mechanics energy minimization of large molecules. Journal of Computational Chemistry 8: 1016-1024.
